# Metal‐free Hydrogen Generation from Seawater and Silicon Waste: A Circular Approach

**DOI:** 10.1002/anie.9919359

**Published:** 2026-06-04

**Authors:** Mustapha Hamdaoui, Anja Steinmaurer, Philipp Jernej, Daniel Legenstein, Peter Hartmann, Adrian Daniel Boese, Katalin Barta

**Affiliations:** ^1^ Institute of Chemistry University of Graz Graz Austria

**Keywords:** hydrogen, metal‐free catalysis, polymer recycling, seawater, waste silicon

## Abstract

Hydrogen is a primordial energy carrier and industrial raw material that will play a crucial role in future energy transition and decarbonization ambitions. Silane hydrolysis represents an under‐explored approach to hydrogen generation, yet most known systems have thus far focused on noble‐metal catalysis. Herein we report a highly active transition metal‐free method for the production of hydrogen from (sea)water and hydrosilanes, relying on the unique combination of simple bases as catalyst, and dimethyl sulfoxide as promoting solvent, assisted by mechanistic and computational studies. Remarkably, the hydrolysis of phenylsilane achieved a turnover frequency (TOF) of 202 ± 5 min–^1^, far superior to existing metal‐free systems (TOF ≤ 8 min–^1^), and even surpassing noble‐metal catalysts (TOF ≤ 170 min–^1^). The developed method rapidly generates hydrogen directly from seawater, applying polymethylhydrosiloxane (PMHS), a silicon industry waste, with rates up to 70.2 mol_H2_/g_cat_/h. Notably, we have demonstrated the recyclability of the solvent, and re‐use of the by‐products formed during PMHS hydrolysis. Thus, the polysiloxane by‐products were efficiently depolymerized into functional chlorosilane monomers, which are suitable for the design of new high‐value silicon polymers. These findings highlight the broader applicability and circularity of our integrated approach.

## Graphical abstract

Hydrogen was rapidly generated directly from untreated seawater and polymeric Si waste without any transition metal catalyst. The in situ generated polymeric cross‐linked Si resin was completely depolymerized under mild conditions into chloro‐silane monomers. The latter compounds are of high‐value to the silicone industry for the preparation of novel polysiloxanes, thus closing the silicon cycle.

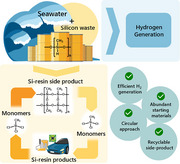

## 1 Introduction

Hydrogen (H_2_) is a centrally important chemical energy carrier and industrial raw material [1, 2]. While the promise of green H_2_ struggles in realizing its foreseen global implementation [1], even the most pessimistic scenarios anticipate that H_2_ will still play a growing role in future high‐value industrial processes [1]. In the net‐zero scenario, the global demand of H_2_ is estimated to be nearly 400 Mt by 2050 (Figure S1) [3]. Currently, the vast majority of H_2_ originates from fossil resources through coal gasification (22%), natural gas reforming (60%) and industry by‐product (e.g., refineries; >15%), overall resulting in about 920 Mt/annum CO_2_ emissions [4]. Thus, it is critically important to develop an array of novel methods for clean hydrogen production (Figure S2) [5]. Beside the most mature technology, namely water electrolysis, it is also interesting to consider catalytic methods for sustainable hydrogen generation [6, 7, 8].

A robust method for H_2_ generation from water, which is not electricity‐driven, is through hydrolysis of hydrosilanes. Previously, silane hydrolysis was predominantly achieved using (noble) transition metal catalysts [9, 10, 11, 12, 13, 14, 15, 16, 17, 18, 19, 20, 21, 22]. The industrial scalability of siloxane‐based liquid hydrogen carriers is currently being pioneered by HSL Technologies (formerly HySiLabs) [23]. This reaction could be rendered much more sustainable and cost‐effective by switching to metal‐free systems. Indeed, the activation of the Si–H bond in the absence of a transition metal was achieved by Grubbs, Stoltz, Zare and coworkers, showing that simple bases could be utilized as catalysts in the silylation of C–H [24, 26] and O–H [27] bonds. Albeit seldom studied, pioneering reports showed feasibility of silane hydrolysis under metal‐free conditions, but the performance remained still far inferior to the transition metal systems [28, 29, 30, 31, 32]. Moreover, the use of potentially hazardous organic solvents and/or additives [33] [e.g., *N*,*N’*‐dimethylpropylene urea (DMPU) [28], hexamethylphosphoramide (HMPA) [30], tetrahydrofuran (THF) [31]; Table S1], the predominant reliance on non‐waste hydrosilanes [34], and the lack of information about key catalytic intermediates as well as experimental demonstration of the recyclability of the studied systems are major weaknesses of previous systems.

More specifically, in their elegant study, Yang and coworkers [28] have found an accelerating effect of DMPU, a reprotoxic chemical, on the hydrolysis of hydrosilanes without the need for additional base. Other similar aprotic dipolar solvents such as dimethyl sulfoxide (DMSO) and dimethylformamide (DMF) did not show the same accelerating effect under these conditions (Table S1). The Fan group [31] reported the hydrolysis of various hydrosilanes in the presence of KOH in deionized (DI) water, in THF as co‐solvent, at room temperature (RT), albeit with moderate efficiency. Brunel has demonstrated that 85% yield H_2_ could be generated from DI‐H_2_O and hydrosilanes using a combination of 30% NaOH and 1.5 mol% HMPA [30]. Similarly to DMPU, HMPA is also known for its high toxicity, and it would be necessary to find suitable alternatives [35, 36, 37]

**FIGURE 1:**
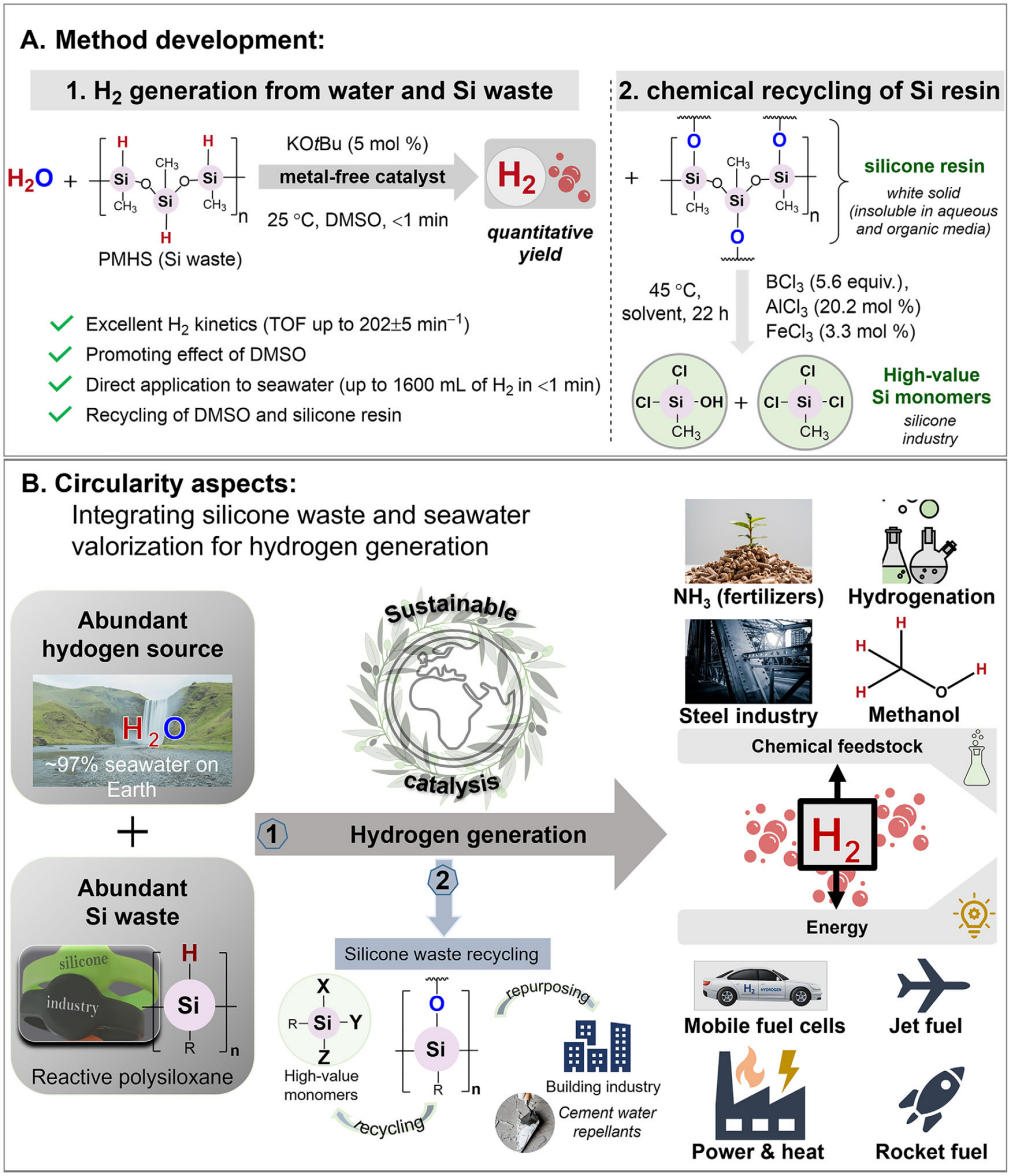
The integrated approach developed in this paper for the metal‐free hydrogen generation from water and waste silicon.

Here we describe the surprising accelerating effect of DMSO, an innocuous and biodegradable solvent [33],[35], in combination with simple bases as catalysts, on silane hydrolysis (Figure 1B). Remarkably, the here‐developed method led to unprecedented hydrolysis rates applicable to a range of hydrosilanes. For instance, the hydrolysis of phenylsilane achieved a turnover frequency (TOF) of 202 ± 5 min^−^^1^ (Table S2), far superior to existing metal‐free systems (TOF ≤ 8 min^−1^) and even outperformed the best transition metal‐based catalysts (TOF ≤ 170 min^−1^). Mechanistic details were scrutinized through a combined synthetic, spectroscopic and computational approach, and an ionic mechanism [25] was proposed based on the isolation and spectroscopic characterization of a key pentavalent ionic silane intermediate K[HSi(OEt)_3_(O*t*Bu)].

Having a powerful method for H_2_ generation in hand, we next addressed broader sustainability and circularity challenges related to feedstock availability, and recycling / re‐use of solvents and byproducts. Conceptually, a highly attractive approach would be to integrate hydrogen generation with silicon‐waste valorization using affordable feedstocks: seawater and readily available industrial polymeric waste. Of particular interest is polymethylhydrosiloxane (PMHS), which contains many reactive hydride units (ca. 25–35 [38] per polymer unit). Thus, we envisioned an integrated approach where the waste hydrosilane PMHS is valorized into H_2_ using seawater while the silicone resin co‐generated in the process is repurposed or recycled (Figure 1B).

In this context, we have shown that, using non‐treated seawater and PMHS, an impressive 1600 mL of H_2_ can be produced in under one minute using only 6.5 g of PMHS waste and 1 mL of untreated seawater (TOF = 43.8 min^−1^). Subsequently, the solvent was recycled and reused in catalysis while the generated, water insoluble polysiloxane (abbreviated as Si resin) was removed by simple filtration and efficiently depolymerized under mild conditions. The here‐developed powerful depolymerization method yields MeSiCl_3_ and MeSiCl_2_OH, valuable building blocks for the synthesis of various silicone materials [39] (Figure 1A).

## 2 Results and Discussion

As earlier stated, our ultimate goal was to identify a novel metal‐free catalytic method for the rapid generation of H_2_ from seawater and waste PMHS as abundantly available and benign feedstock, and devise a viable strategy for the recycling and/or repurposing of the silicone resin co‐generated in the process. First, we describe the set of experiments aimed at identifying a highly active metal‐free catalytic system for hydrogen generation from water and a series of hydrosilanes bearing alkyl or phenyl substituents. Next, we evaluated the mechanistic aspects of the catalytic system by a combined experimental, spectroscopic and computational approach.

In the last section, we discuss the results and implications of metal‐free hydrogen generation from seawater integrated with Si waste recycling.

### 2.1 The Development of a Novel Method for Hydrogen Generation From Silanes

#### 2.1.1 Impact of DMSO on the Hydrogen Generation Rate

Based on prior work, we first studied the impact of several solvent systems on the H_2_ generation from water and hydrosilanes, using catalytic amounts of inexpensive, innocuous bases. HSiEt_3_ was chosen as it reacts sluggishly with water under basic conditions, thus allowing for more accurate and reliable comparison between the different measurements. The reaction efficiency was evaluated by directly measuring the released H_2_ volume V_(H2)_ as function of time and comparing the initial reaction rates derived from the time‐dependent V_(H2)_ data (Figure S3).

**FIGURE 2:**
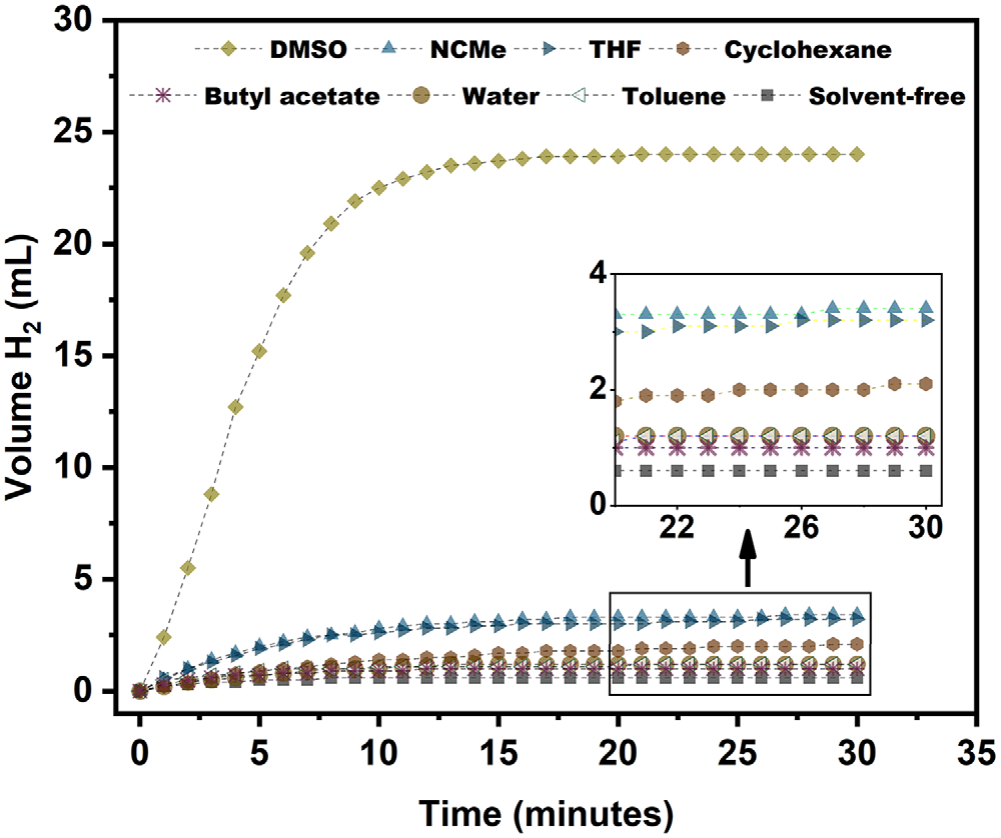
The effect of different solvents + control experiment without any added solvent on the volume of hydrogen generated from the reaction of H_2_O and HSiEt_3_. Conditions: HSiEt_3_ (129 mg, 1.11 mmol), H_2_O (0.02 mL, 1.11 mmol), KO*t*Bu (6.23 mg, 0.055 mmol), DMSO (0.5 mL). The maximum theoretical V_H2_ that can be released from 1.11 mmol H_2_O is ≈ 24.5 mL.

The results of hydrogen generation in different solvent systems, using triethylsilane (HSiEt_3_) and DI‐water as well as 5 mol% of KO*t*Bu are displayed in Figure 2. Interestingly, the silane hydrolysis rate was found dominant in DMSO, compared to all other solvents tested. Specifically, in the presence of DMSO, 98% yield of H_2_ (V_(H2)_ ≈ 24 mL; max. theoretical V_(H2)_ ≈ 24.5 mL) was released in >15 min corresponding to an initial reaction rate of 1.02 × 10^−1^ mM/min (Table S3). While in all other solvents tested, only ≤10% H_2_ was released in 30 min, corresponding to initial reaction rates ranging 1.18 × 10^−2^‐4.10 × 10^−3^ mM/min, which are 10–100 times lower than that obtained using DMSO.

#### 2.1.2 Impact of the Base Catalyst

**FIGURE 3:**
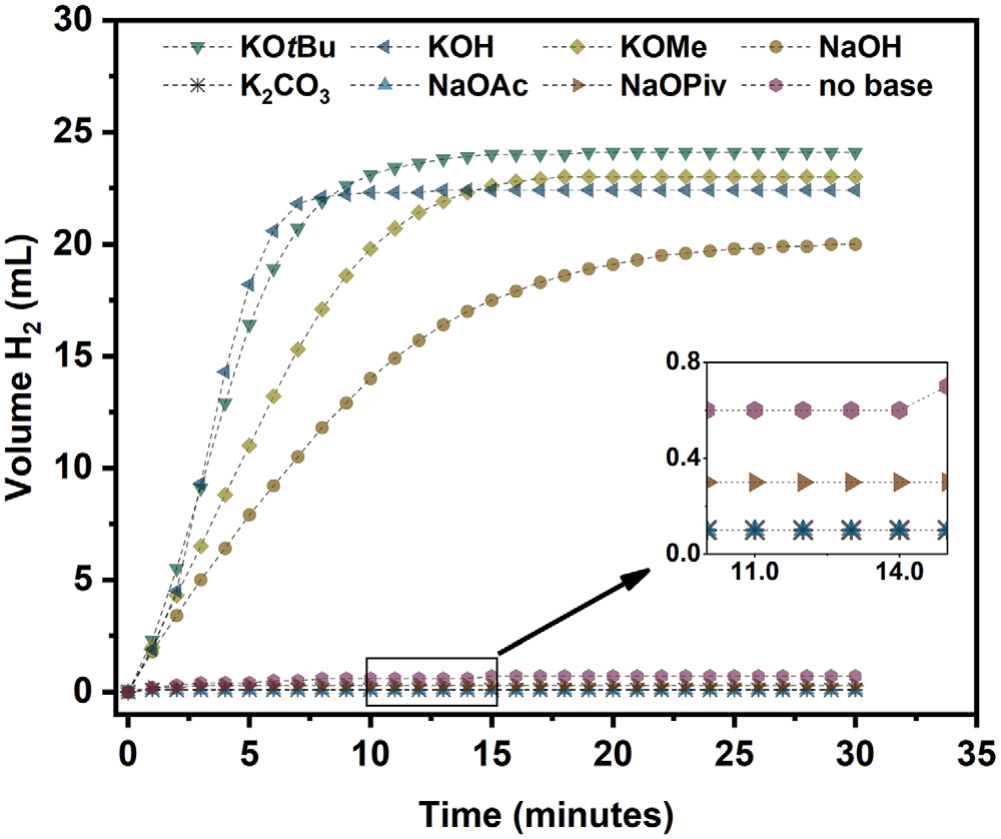
The effect of different catalysts + control experiment without any added base on the volume of hydrogen generated from the reaction of H_2_O and HSiEt_3_. Conditions: HSiEt_3_ (129 mg, 1.11 mmol), H_2_O (0.02 mL, 1.11 mmol), KO*t*Bu (6.23 mg, 0.055 mmol), DMSO (0.5 mL). The maximum theoretical V_H2_ that can be released from 1.11 mmol H_2_O is ≈ 24.5 mL.

Next, a series of organic and inorganic base catalysts of varying p*K*_b_ (Table S4) [40, 41] were evaluated using HSiEt_3_ and DI‐water, using 5 mol% base. The results are shown in Figure 3. Control experiments conducted without base or without HSiEt_3_ showed no hydrogen generation. A general trend was observed, where relatively weak bases (NaOAc, NaOPiv and K_2_CO_3_) showed almost no catalytic activity, while stronger bases (NaOH, KOH, KOMe and KO*t*Bu) afforded much improved hydrogen generation rates. The catalytic activity increased in the order: NaOH < KOMe < KOH ≤ KO*t*Bu. However, although very similar initial reaction rates were observed for both KOH (1.01 × 10^−1^ mM/min) and KO*t*Bu (1.04 × 10^−1^ mM/min), a somewhat higher H_2_ yield was obtained for KO*t*Bu (V_(H2)_ ≈ 24.1 mL; 98% yield) versus KOH (V_(H2)_ ≈ 22.4 mL; 91% yield). For this reason, KO*t*Bu was used for all further studies.

The better performance of KO*t*Bu and KOH in catalyzing the hydrogen generation from water and HSiEt_3_ may be explained by their higher basicity, when compared to the other bases tested (Table S4). Further screening of the base loading showed that 5 mol% KO*t*Bu gave optimal conversion and reaction rate (Figures S4‐5; Table S5).

#### 2.1.3 Impact of the Hydrosilane

**FIGURE 4:**
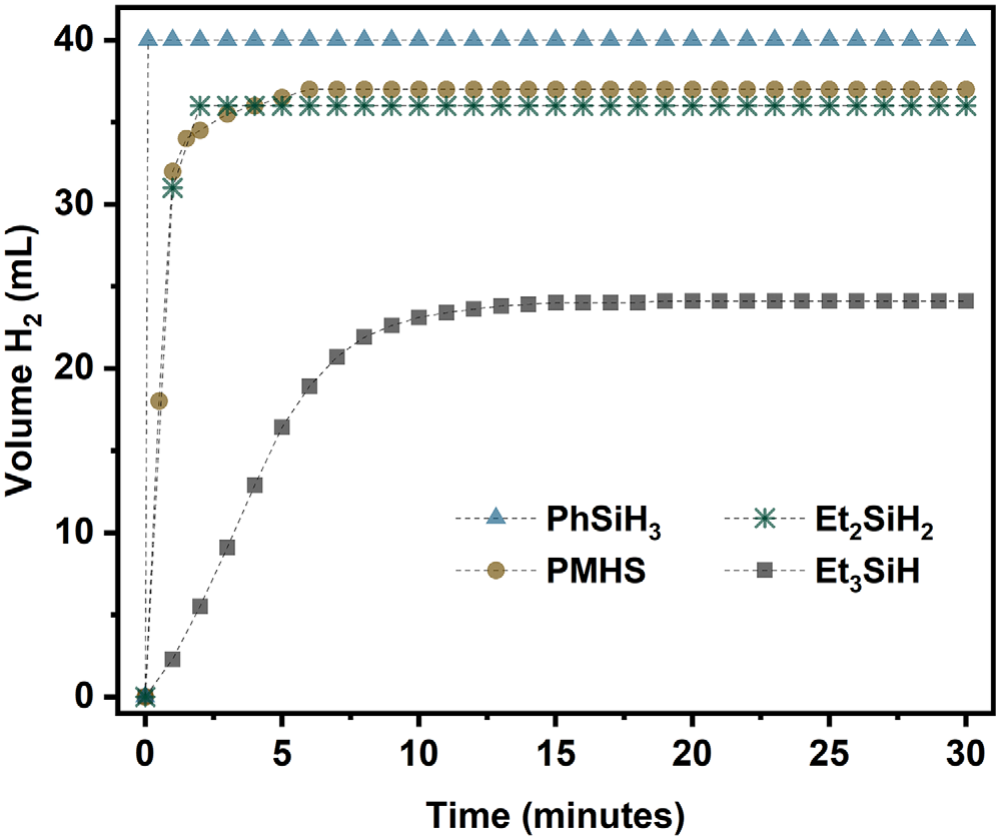
The effect of different hydrosilanes on the volume of hydrogen generated from the reaction of H_2_O and HSiEt_3_, Et_2_SiH_2_, PhSiH_3_ and PMHS. Conditions: hydrosilane (1,11 mmol), H_2_O (0.02 mL, 1.11 mmol), KO*t*Bu (6.23 mg, 0.055 mmol), DMSO (0.5 mL). The maximum theoretical V_(H2)theo._ that can be released from reaction of 1.11 mmol H_2_O with HSiEt_3_ is ≈ 24.5 mL. For other cases, due to the availability of at least of two hydrides that can react with the two protons of H_2_O, the expected maximum is V_(H2)theo._≈ 49 mL.

In order to probe the effect of varying the substituent at silicon in the hydrosilane, HSiEt_3_ was compared with diethylsilane (Et_2_SiH_2_), phenylsilane (PhSiH_3_) and polymethylhydrosiloxane (PMHS). PMHS is particularly interesting because it is an abundant and cheap waste derived from the silicon industry [42], besides being bench stable and non‐toxic liquid with a relatively low boiling point (142°C) [38]. While the PMHS utilized in our standardized laboratory studies was obtained from commercial sources to ensure kinetic reproducibility, PMHS on an industrial scale represents a massive, underutilized waste by‐product of the Müller‐Rochow process. Figure 4 provides a comparison of the hydrogen generation kinetics for all the selected hydrosilanes. PhSiH_3_, Et_2_SiH_2_ and PMHS show outstanding kinetics with respect to hydrogen generation. In the case of PhSiH_3_ 89% H_2_ is generated in <25 s, corresponding to an initial reaction rate of 9.06 × 10^−1^ mM/min and an impressive TOF of 202 ± 5 min^–^^1^ (calculated for the first H_2_ equivalent; see Figure S6 and Movie S1), which is much higher compared to metal‐free or transition metal‐based systems (Table S2).

In the case of PMHS, 65% H_2_ yield was achieved in <60 s corresponding to an initial reaction rate of 7, 25 × 10^−1^ mM/min and an excellent TOF of 43.8 min–^1^. This reaction continued to evolve hydrogen for 5 min yielding a total of 76% H_2_ yield. Et_2_SiH_2_ was also found to be a very potent hydride source for this reaction since 61% H_2_ yield is generated in <60 s corresponding to an initial reaction rate of 7, 02 × 10^−1^ mM/min and a TOF ≈ 40 min^–^^1^. Compared to other silanes, HSiEt_3_ performed the worst with an initial reaction rate of 1.02 × 10^−1^ mM/min.

### 2.2 Mechanistic Considerations

#### 2.2.1 Analysis of the Silicon Byproducts

The reaction between HSiEt_3_ and DI H_2_O (1:1 ratio) in the presence of 5 mol% KO*t*Bu was monitored by in situ NMR analysis in DMSO‐*d*_6_ (Figure S7). The resulting ^1^H spectrum showed a resonance peak at 4.56 ppm identified as H_2_ alongside those attributed to the Si by‐products (Et_3_SiOSiEt_3_ and HOSiEt_3_). This NMR assignment was confirmed by comparison with NMR spectra of the isolated two fractions following a bulb‐to‐bulb vacuum distillation (Figures S7/S10). ^29^Si NMR spectra showed that the resonance peak of HSiEt_3_ at 7 ppm disappeared and a new signal of the disiloxane product Et_3_SiOSiEt_3_ was observed at ca. 9 ppm; the ^29^Si resonance for HOSiEt_3_ expected at ca. 18 ppm [43] could not be detected most likely because of its low concentration. Further analysis of the reaction mixture via gas chromatography (GC‐MS) confirmed the formation of both Si by‐products HOSiEt_3_ and Et_3_SiOSiEt_3_ (Figures S9/S11).

#### 2.2.2 Analysis of the Evolved Gas

To further confirm that H_2_ was indeed generated, the gas evolved from the reaction mixture was bubbled into a solution of toluene‐*d*_8_. Direct ^1^H NMR analysis of the latter solution revealed only one resonance singlet peak at 4.50 ppm that is unambiguously assigned to H_2_ (Figure S12). GC analysis using a thermal‐coupled detector (TCD) also confirmed that H_2_ was indeed the only gas generated (Figure S13). Indirect detection of H_2_ was also employed, using cyclohexene as the substrate of hydrogenation (Figure S14). This reaction was chosen because it was reported to be readily catalyzed using Pd/C at RT and 1 atmosphere of H_2_ [44]. The complete hydrogenation of cyclohexene was observed (99% GC yield), further confirming the generation of H_2_ (Figure S15).

While other gases (e.g., methane, ethane) could potentially evolve from the decomposition of DMSO or HSiEt_3_, none could be detected. Control experiments showed that negligible traces of water were present in either DMSO and HSiEt_3_ (Figure S16, Table S6). In addition, several controls ruled out the potential formation of SMe_2_ from the deoxygenation of DMSO in the presence of HSiEt_3_ (Figures S17–19) [45]. For instance, catalysis experiments using isotopically labelled H_2_^18^O demonstrated that the oxygen incorporated in the Si byproducts originated from water and not from DMSO (Figures S20,21).

#### 2.2.3 On the Potential Contribution from Transition‐metal, Radical or Light Mediated Catalysis

In order to rule out noble metal contamination [46, 47] analysis by ICP‐MS (inductively coupled plasma mass spectrometry) of the DMSO and KO*t*Bu samples used throughout this study revealed a negligible metal content (e.g., Ag, Pt, Pd, Rh, Ir ≤ 50 ppb; more data in Table S7). Next, we probed the change of kinetics of H_2_ generation induced by the addition of 10 ppm of different metal precursors to the standard metal‐free catalysis mixture (Figure S22) with no deviations found. Further orthogonal poisoning experiments using strong aprotic metal chelators (10 mol% 2,2′‐bipyridine or triphenylphosphine) showed no deviation in the hydrogen evolution kinetics (Figure S24), further confirming that the rapid catalysis is most likely a metal‐free process independent of trace transition metal impurities.

No abnormal deviation in catalysis rates were determined in controls verifying the possible involvement of trace metals or other impurities that might originate either from different chemical suppliers or material (e.g., stir bar, borosilicate glass) used for the reaction (Figures S23 & 25–27). Further controls of the H_2_O/Et_3_SiH/KO*t*Bu/DMSO system indicated no contribution from radical‐ [48] or photo‐initiation [49, 50] to the reaction mechanism (Figure S28).

#### 2.2.4 Isolation of a Catalytic Intermediate and Its Reactivity

**FIGURE 5:**
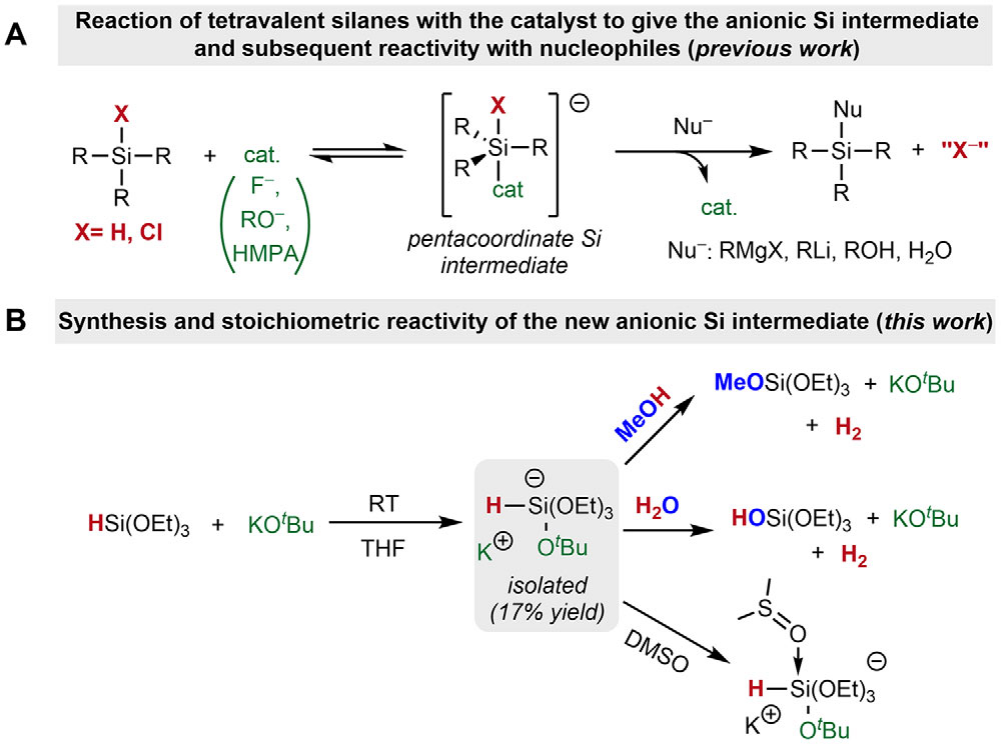
(A) Reported work on the generation of the anionic pentacoordinate Si intermediate from tetravalent silanes and nucleophilic catalyst, and its subsequent reactivity with stoichiometric nucleophiles [51, 52]. (B) Isolation of the new anionic pentacoordinate Si intermediate K[HSi(OEt)_3_(O*t*Bu)] and its reactivity towards H_2_O, MeOH and DMSO (*this work*).

Next, we aimed at delineating the roles of KO*t*Bu and DMSO in our catalysis system. Previous work [51] has shown that reactions of tetravalent silanes with nucleophilic catalysts (e.g., F^−^, RO^−^, HMPA) give an anionic pentacoordinate Si intermediate which subsequently reacts with nucleophiles at the silicon atom to give new silicon products, releasing leaving group X^–^ (Figure 5A). For instance, Corriu and coworkers [52] have observed instantaneous hydrogen evolution when the pentacoordinate species K[HSi(O‐*i*Pr)_4_] was mixed with H_2_O. No reaction occurred between HSi(O‐*i*Pr)_3_ and H_2_O alone after 5 days.

Using the synthetic procedure developed by Corriu [52], we have isolated a novel anionic pentacoordinate Si intermediate K[HSi (OEt)_3_(O*t*Bu)] in 17% yield by reaction of HSi(OEt)_3_ with 1 equiv. of KO*t*Bu in THF (RT, 4 h) (Figures 5B; SI, section 9a). Such a hydrosilicate ion bearing a *tert*‐butoxy moiety is the subject of a growing debate in the literature, especially from the perspective of mechanistic investigation of the Stoltz–Grubbs KO*t*Bu‐Et_3_SiH system [24, 25, 48, 53]. Many organosilicates were previously characterized by NMR spectroscopy and/or X‐ray diffraction [54, 55] To our best knowledge, the new silicate adduct K[HSi(OEt)_3_(O*t*Bu)] represents the first *tert*‐butoxy‐based silicate species from alkoxide/hydrosilane systems to be isolated and analytically characterized. The similar species Na[H_2_Si(Ph)_2_(O*t*Bu)] was observed by in situ NMR studies only and could not be isolated due to its high fluxional behavior and reactivity [53].

Next, the potential role of K[HSi(OEt)_3_(O*t*Bu)] during catalysis was investigated by conducting a series of stoichiometric reactions with DI‐H_2_O, MeOH and DMSO (NMR in toluene‐*d*_8_) (SI, section 9b). First, reaction of K[HSi(OEt)_3_(O*t*Bu)] with MeOH revealed the formation of the expected products MeOSiEt_3_, KO*t*Bu and H_2_, whereas the same reaction with H_2_O led to the exhaustive hydrolysis of K[HSi(OEt)_3_(O*t*Bu)] into mainly Si(OH)_4_ and EtOH, accompanied by H_2_ and KO*t*Bu. However, when K[HSi(OEt)_3_(O*t*Bu)] was mixed with DMSO (53 equiv.), ca. 50% of K[HSi(OEt)_3_(O*t*Bu)] was converted to the presumed adduct K[HSi(DMSO)(OEt)_3_(O*t*Bu)] whereby DMSO coordinates to Si. The latter observation could explain in part the specific accelerating effect induced by DMSO during catalysis, due to its role in stabilizing anionic intermediates such as K[HSiR_3_(O*t*Bu)] [56, 57, 58]

**FIGURE 6:**
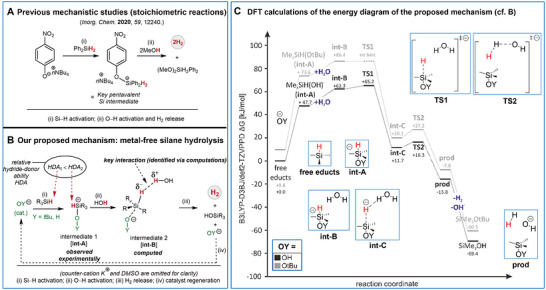
(A) Previous mechanistic studies by Shubina et al. [59]. (B) Our proposed mechanism for the silane hydrolysis developed in this study. (C) DFT calculations of the energy diagram of the proposed mechanism of the reaction of Me_3_SiH with H_2_O, assuming OH^–^ and O*t*Bu^–^ to be the catalyst (abbreviated OY^–^), respectively and employing the COSMO implicit solvation model. An extended version that also accounts for possible subsequent reaction steps can be found in the SI. To be able to compare both OH^–^ and O*t*Bu^–^ on the same energetic scale, OH^–^ was chosen as the energetic reference and the reaction energy of HOtBu with OH^–^ was used to determine the energy of the free educts in the O*t*Bu^–^ case.

Finally, when K[HSi(OEt)_3_(O*t*Bu)] was used as catalyst (5 mol%) in the reaction of HSi(OEt)_3_ with DI‐H_2_O in the presence of DMSO, the hydrogen evolution kinetics were very similar to those observed for the reaction involving KO*t*Bu as catalyst (Figure S44; SI, section 9c). Thus, along with the above observations, this experiment strongly suggests the involvement of the adduct of general formula K[HSiR_3_(OY)] (OY = O*t*Bu, OH; **int‐A** in Figure 6) as a plausible catalytic intermediate in the silane hydrolysis reaction, which is also supported by our computations (see below).

#### 2.2.5 An Ionic Reaction Mechanism Is Proposed

Based on our experimental observations and the prior work of Voronova et al. [59], who studied the *stoichiometric* methanolysis of diphenylsilane (Ph_2_SiH_2_), we propose a mechanism for our metal‐free catalyzed silane hydrolysis (Figure 6B) and set out on a preliminary computational study to further validate its plausibility. To account for the formation of the disiloxane Et_3_SiOEt_3_ as the major Si byproduct, a mechanism is proposed in Figure S45.

DFT calculations of the energy profile were conducted using the simplified silane model HSiMe_3_ to eliminate any potential influence of the conformational space (the results were subsequently verified with calculations on the HSiEt_3_ system revealing that the qualitative picture is retained; see SI). While the O*t*Bu‐bound intermediate provides a structurally isolated baseline, the rapid equilibrium with water suggests that hydroxide (OH^–^) is also a highly likely active catalytic species under standard reaction conditions. Thus, due to the complex nature of the water‐DMSO system and largely comparable results between KOH and KO*t*Bu, we opted to carry out our computational investigations with both OH^–^ and O*t*Bu^–^ as potential catalysts (denoted collectively OY^–^). At this stage of the study, we speculate that the prompting effect of DMSO is due to the great solvation of K^+^, yielding the highly reactive ‘naked’ pentavalent silicate nucleophiles.

In the first step of the mechanism, the base anion OY^–^ adds to the electrophilic silicon atom of HSiR_3_ to yield the pentavalent intermediate [HSiR_3_(OY)]^–^ (**int‐A**). Whereas this addition could, *a priori*, lead to either an axial or equatorial arrangement of the OY moiety in **int‐A**, our calculations indicate the axial species to lead to the overall lower energy pathway, which is in accordance with the work of Voronova and coworkers [59] (a detailed comparison of the axial and equatorial reaction pathways is given in the SI, section 9e). Formation of **int‐A** is highly endergonic for both investigated catalysts (ΔG_OH‐_ = 47.7 kJ/mol and ΔG_O_ *_t_*_Bu‐_ = 63.8 kJ/mol). Next, adduct complex **int‐B** is generated by H_2_O forming a dihydride H^δ–^•••H^δ+^ interaction [60] with the silicon‐bound hydrogen, which is conceptually analogous to ammonia borane systems [61]. Additionally, we propose that the coordination of the base catalyst to the silane leads to an increased hydride character in the involved hypervalent pentacoordinate species. Indeed, NMR data showed a shielded ^1^H resonance for the hydride in isolated K[HSi(OEt)_3_(O*t*Bu)] compared to free HSi(OEt)_3_ (SI, section 9a). Together, these factors manifest in a very low additional activation energy for the subsequent **TS1**, where the H‐Si bond breaks to form a hydride (**int‐C**). Nevertheless, **TS1** constitutes the overall highest energy barrier in the reaction mechanism and is, hence, rate determining. Overall, the OH^−^ pathway is energetically favored (ΔΔ*G* = 24.1 kJ/mol). Subsequently, one hydrogen atom from water binds with the hydride from HSiR_3_ (**TS2**), thereby generating an agglomerate of the reaction products Me_3_SiOY, H_2_ and OH^–^ (**prod**). Subsequent escape of H_2_ and dissolution of the agglomerate reveals the overall reaction to be highly exergonic irrespective of the investigated catalyst. In sum, the reaction of **int‐A** with H_2_O generates one equivalent of H_2_ alongside one equivalent of HOSiR_3_, thereby regenerating the base catalyst.

Overall, the computational data predict OH^–^ to be a much more effective catalyst than O*t*Bu^–^. Judging from the p*K*_A_ values of HO*t*Bu and H_2_O in DMSO, which are 32.2 and 31.4, respectively, the equilibrium can also be slightly expected to favor OH^–^. Together with the similarity of the respective V_H2_ vs time curves in Figure 2, and the generation of hydroxide during the reaction, this suggest the main catalytically active species to be OH^–^ for both KO*t*Bu and KOH catalytic systems. The postulated mechanism is also in line with the experimental isolation and catalytic activity of K[HSi(OEt)_3_(O*t*Bu)]. Contrary to its congeners HSiMe_3_(OY) and HSiEt_3_(OY) (see Figure 6C and the SI/section 9e, respectively), which display very high Gibbs free energies, the formation of HSi(OEt)_3_(OY) is computationally predicted to be energetically stable enough to be isolable [ΔG(O*t*Bu) = +9.3 kJ/mol; see the SI for details]. When subjected to water, HSi(OEt)_3_(O*t*Bu) can be expected to readily undergo reaction, releasing OH^–^ and H_2_ in the process, with the former potentially playing an active role throughout the subsequent reaction cycles.

### 2.3 Towards an integrated and circular strategy to derive hydrogen from silicon waste and seawater

#### 2.3.1 Hydrogen Generation From Seawater and Waste PMHS

**FIGURE 7:**
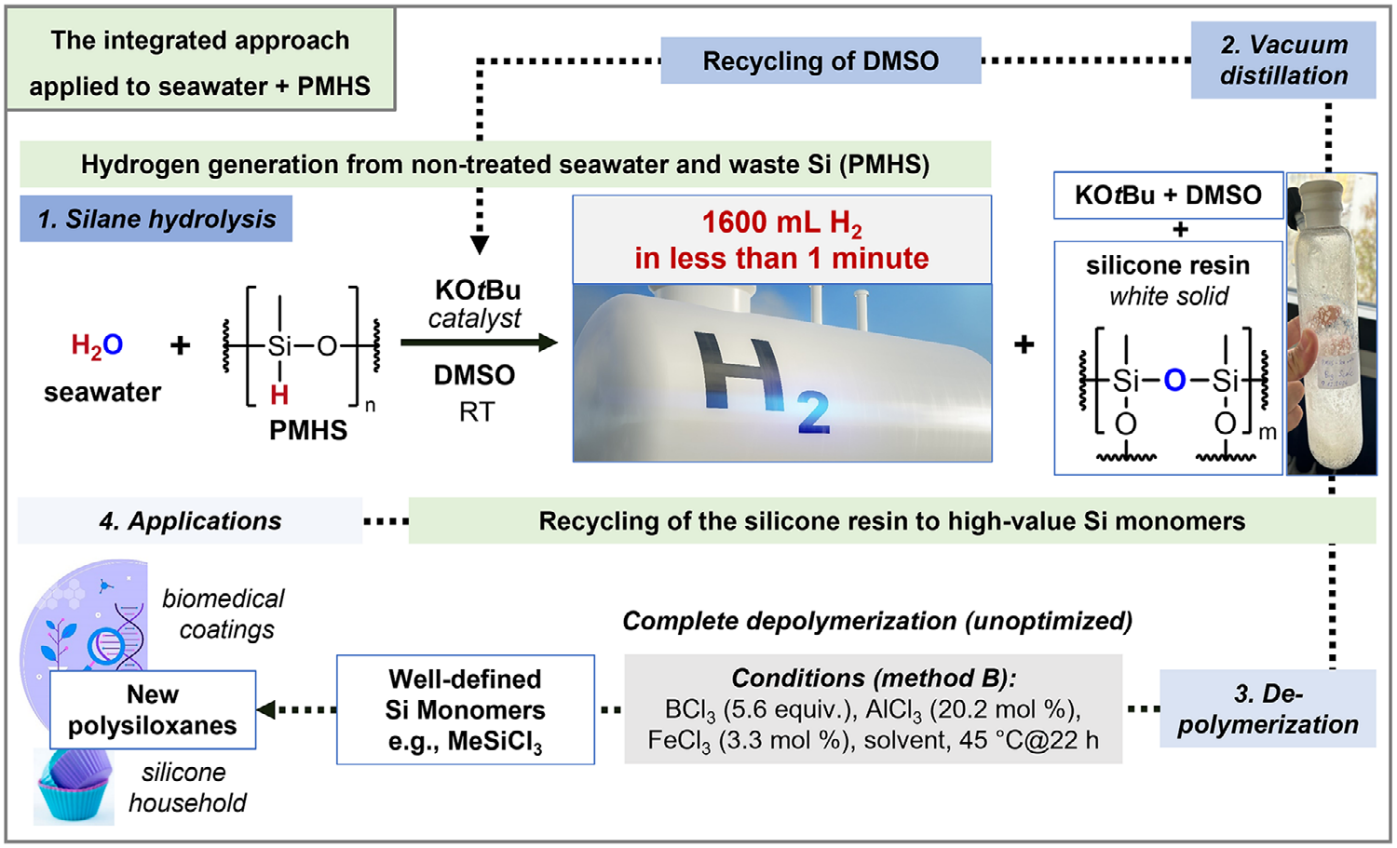
Demonstration of the integrated approach described in this study: Hydrogen generated from the reaction of sea H_2_O and PMHS in combination with the proposed recycling strategy. Conditions: PMHS (6.5 g, 111 mmol), H_2_O (1 mL, 55.5 mmol), KO*t*Bu (62 mg, 27.5 mmol), DMSO (2 mL). The maximum theoretical V_(H2)theo._ that can be released from the reaction of 55.5 mmol sea H_2_O with 111 mmol PMHS is ≈ 2486 mL (if the two H atoms of H_2_O are consumed).

Planet Earth is 97% covered with seawater, yet conversion of this abundant natural source into H_2_ is still at its infancy. Electrolysis of seawater to H_2_ is the most promising approach [62, 63, 64, 65]. Much research is currently directed to increasing the performance of electrolyzers against the deleterious effect of chloride and other anions originating from the various salts present in seawater. Additional concerns include elimination of environmental hazards and reducing contributions to climate change from the release of chlorine and other gases [62]. The approach presented in this paper represents a possible alternative for H_2_ generation, by directly using non‐treated seawater through silane hydrolysis. Thus, gratifyingly we observed that H_2_ can indeed be readily produced from Mediterranean seawater instead of DI water, without any significant changes to the hydrogen evolution kinetics. As a proof‐of‐concept, 1600 mL H_2_ could be generated in less one minute using 1 mL of seawater and 6.5 g of PMHS [38] (Figure 7). While controlled‐addition experiments (15°C) successfully suppressed initial H_2_ bursts, late‐stage acceleration due to resin viscosity indicates that achieving steady‐state flow requires dedicated reactor engineering (Figure S65). Likewise, high efficiencies were observed when using other seawater sources (Figure S66). These results showcase promise for future medium to large scale applications where on‐site and on‐demand hydrogen is rapidly needed using non‐treated seawater, while alleviating some of the drawbacks associated with the electrolysis method (e.g., chlorine release). As detailed in the Supporting Information (SI‐section 3), calculations based on global silicone production metrics demonstrate that valorizing the unavoidable PMHS waste stream could sustainably generate kiloton‐scale volumes (e.g., >1,600 metric tons [66] of high‐purity hydrogen annually from a single, underutilized source. Control experiments confirmed that seawater ions neither inhibit the catalytic kinetics (Figure S67) nor contaminate the final product, as residual salts are readily removed via a simple water/methanol wash prior to resin recycling (see SI section 12).

#### 2.3.2 Recovery, Reuse and Recyclability

The reaction of PMHS with water, under specific catalytic conditions, yields a cross‐linked silicone resin structurally assignable to a polysilsesquioxane of T‐type bonding motif (formally as ′RSiO_3_^′^ monomeric formula) [67]. This T‐type structure results from the condensation of the in situ generated silanol units Me*_n_* Si(OH)*_m_* O*_y_*. Because of its low miscibility in aqueous or organic media, the silicone resin precipitates from the reaction medium as a white solid, allowing for recovery and repurposing into either new silicone materials [68], silicone adhesives (e.g., in skin adhesion [69]) or as siloxane‐based water repellents for the building industry [70] (e.g., Wacker Chemie AG is actively developing sustainable silicone materials for the cement durability).

Thus, in the reactions involving PMHS‐based H_2_ generation, we have observed the formation of a white silicone resin (Si Resin; Figure 7), and envisioned a recycling strategy where DMSO could be recovered and reused, while the Si resin could be selectively degraded into usable Si monomers (Figure 7).

For the recovery of the solvent, the resin contaminated with both DMSO and KO*t*Bu was subjected to vacuum distillation, with 96% DMSO recovery in high purity (Figure S68), while recovery of KO*t*Bu was not attempted due to its catalytic use. To confirm reusability in practical applications, the recovered DMSO was utilized across three consecutive hydrogen generation cycles; remarkably, no significant change in catalytic activity or kinetic performance was observed (see SI, Figure S69 for full kinetic profiles).

While very challenging to achieve, selective chemical recycling of this silicone resin into high‐value Si monomeric building blocks is vital for a circular strategy herein, and would be highly promising for the silicone industry overall [39]. Thus, we next dealt with repurposing the dried resin. Its molecular structure was determined by solution ^1^H NMR (Figure S70), solid state ^29^Si NMR (Figure S71) as well as infrared spectroscopy (Figure S78). As expected, all analyses showed that the resin is a result of the oxidation of the Si‐H moieties of PMHS into silanols, which then self‐condense into siloxane bridges (Figure 7). These monomeric units can be compared to T‐resin monomers. The presence of such monomers would suggest the formation of polysilsesquioxanes, which was confirmed by comparison to independently synthesized Si resins with established structures (Figures S72–77) [71].

Döhlert and Enthaler presented the degradation of silicon byproducts, into the volatile monomers MeSiHF_2_ and MeSiF_3_ using BF_3_·OEt_3_ [72]. The main drawbacks of this approach are the use of excess BF_3_·OEt_2_, the elevated temperature and the regeneration of gaseous monomers. Another promising method leverages the catalytic activity of tetrabutylammonium fluoride in THF to depolymerize various silicone polysiloxane materials at room temperature [73, 74] Unfortunately, while we observed extensive degradation of our Si‐resin using this approach, the resulting Si monomers could not be adequately identified.

Hence, we applied the two alternative methods A and B (Figure 7) [39]. In method A, pioneered by Montail, Raynaud and coworkers, BCl_3_ was combined with catalytic FeCl_3_ in dichloromethane (DCM) at 45°C for the depolymerization of our Si resin, yet the process was incomplete (SI, section 12). Therefore, we have developed a dual catalytic approach, by applying both AlCl_3_ and FeCl_3_ in the presence of an excess BCl_3_ in DCM at 45°C (method B). Gratifyingly, this led to exhaustive depolymerization within 22 h yielding mainly MeSiCl_2_OH and MeSiCl_3_ (1:0.4 molar ratio) (SI, section 12). These functional methylchloro(hydrido)‐monosilanes are important Si building blocks used for the preparation of polysiloxanes by the silicone industry for application in, e.g., household consumables, biomedical coatings, or as mouldings, adhesives, composites, sealants and resins by key industry sectors (building, automotive or electronics). Likewise, when a T‐type Si resin sample (independently prepared from the hydrolysis of MeSiCl_3_) was subjected to method B (SI, section 12), the same depolymerization mixture was obtained, further confirming the robustness of our new approach in degrading highly cross‐linked Si resins [39]. Although a stoichiometric excess of BCl_3_ was employed to ensure complete depolymerization on a laboratory scale, the high volatility of the reagents and the selective formation of monomeric chlorosilanes (e.g., MeSiCl_3_) make this process highly amenable to standard industrial recycling and fractional distillation (see SI‐section 12, for a detailed discussion on scalability and atom economy).

## 3 Conclusion

To conclude, we have developed a novel, transition metal free method for hydrogen generation through silane hydrolysis, which significantly outperforms known metal‐free, and even transition metal mediated systems (Table S2). These outstanding kinetics stem from the accelerating effect induced by the combined use of DMSO and KO*t*Bu/KOH. By a combined experimental, mechanistic and computational approach, an ionic mechanism involving a key pentavalent anionic Si intermediate is proposed. However, given the apparent complexity of such a system, in‐depth computations are underway to delineate the possible synergistic effect of DMSO, silane and KO*t*Bu/KOH [48].

We have described an innovative approach integrating hydrogen generation with silicon‐waste valorization using two affordable feedstocks: seawater and PMHS as polysiloxane industry waste. The method is highly efficient, cost‐effective, eco‐friendly and potentially scalable, since only a simple base catalyst (e.g., KOH) and DMSO as co‐solvent are required for generating as much as 1600 mL hydrogen within one minute, using untreated seawater. Recycling of the silicone resin that was co‐generated in the process was readily achieved by depolymerization to afford MeSiCl_2_OH and MeSiCl_3_, two monomers of high‐value for the silicone industry. These achievements showcase the potential for our process to be applied in rapid on‐demand hydrogen generation for energy or chemical feedstock purposes integrated with silicone waste valorization at silicone production factories that are located nearby seashore sites. Moreover, ongoing work addresses involvement of other proton sources derived from waste biomass, with the vision to boost the circular and bioeconomy.

In addition to these advantages, the silane/seawater system drastically limits safety hazards and environmental impact associated with hydrogen generation (e.g., low temperatures, no CO_2_ release). As such, our findings should be of particular interest to those seeking to rapidly generate H_2_ on‐demand and on‐site in an eco‐friendly manner. Besides, given the location of a large part of industrial silicone production in close proximity of seawater, our process would provide an attractive alternative to current waste disposal strategies.

## Author Contributions

**Katalin Barta**: supervision, conceptualization, funding acquisition, project administration, resources, writing ‐ original draft, writing ‐ review and editing. **Daniel Legenstein**: investigation, software, validation, writing ‐ review and editing. **Anja Steinmaurer**: investigation, methodology, validation, writing ‐ review and editing. **Philipp Jernej**: investigation, validation, writing ‐ review and editing. **Mustapha Hamdaoui**: conceptualization, methodology, data curation, investigation, validation, formal analysis, supervision, visualization, writing ‐ review and editing, writing ‐ original draft. **Peter Hartmann**: investigation, methodology, software, validation, writing ‐ review and editing. **Adrian Daniel Boese**: supervision, conceptualization, resources, writing ‐ review and editing.

## Conflicts of Interest

The authors declare no competing financial interests.

## Supporting information




**Supporting File 1**: anie73011‐sup‐0001‐SuppMat.pdf.

**Supporting File 2**: anie73011‐sup‐0002‐Movie S1.mp4.

## Data Availability

The data that supports the findings of this study are available in the supplementary material of this article
