# Supplementary material for: Metal‐free Hydrogen Generation from Seawater and Silicon Waste: A Circular Approach
Source: Angew Chem Int Ed Engl. 2026 Jun 4;65(32):e9919359. doi: 10.1002/anie.9919359 (PMC13427148; doi:10.1002/anie.9919359)
Supplement: Supplementary file 1 — Supporting File 1: anie73011‐sup‐0001‐SuppMat.pdf. [file ANIE-65-e9919359-s001.pdf]

Supporting Information for:

Metal-free hydrogen generation from seawater and silicon waste: a circular approach

Mustapha Hamdaoui,*[a] Anja Steinmaurer,[a] Philipp Jernej,[a] Daniel Legenstein, Peter Hartmann,[a] Adrian Daniel Boese,[a] and Katalin Barta*[a]

[a] Dr. M. Hamdaoui, Mag. A. Steinmaurer, P. Jernej, D. Legenstein, Dr. P. Hartmann, Prof. Dr. A. Daniel Boese, Prof. Dr. K. Barta
Institute for Chemistry
University of Graz
Heinrichstraße 28/II, 8010 Graz, Austria
E-mail: mustapha.hamdaoui@uni-graz.at, katalin.barta@uni-graz.at

Table of contents

1. Supporting data for the Introduction section 4
2. General considerations 9
3. Representative procedure for hydrogen generation 10
4. Catalysis development 11
5. NMR and GC analyses of the catalysis mixture 14
6. Hydrogen detection and controls 17
7. Control experiments to exclude metal impurities 24
8. Control experiments to exclude radical pathway 28
9. Mechanistic aspects 30
10. *Hydrogen evolution profile for the controlled-addition experiment* 51
11. Control using different sources of seawater 52
12. Recovery, reuse and recyclability 54
13. References 72

Selected List of Tables

Table S 1. Dipolar Aprotic Solvents Arranged by Current Understanding of Reprotoxicity. Reprinted from J. Sherwood, T. J. Farmer, J. H. Clark. Catalyst: Possible Consequences of the N-Methyl Pyrrolidone REACH Restriction. Chem **2018,** 4, 2010-2012, Copyright (2025), with permission from Elsevier.

Table S 2. Comparison of the efficiency of our approach with previous metal-free and metal-based catalytic systems for the hydrolysis of Et3SiH, Et2SiH2, PhSiH3 and PMHS at 298 K.

Table S 3. Initial rates obtained for the silane hydrolysis reaction in different solvents.

Table S 4. Measured and estimated pKb values of the conjugate acids for various bases in DMSO.

Table S 5. Initial rates obtained for the silane hydrolysis reaction using different concentrations of the catalyst KOtBu.[a]

Selected List of Figures

FIGURE S 1 4
FIGURE S 2 5
FIGURE S 3 10
FIGURE S 4 11
FIGURE S 5 12
FIGURE S 6 14
FIGURE S 7 15
FIGURE S 8 15
FIGURE S 9 16
FIGURE S 10 16
FIGURE S 11 17
FIGURE S 12 18
FIGURE S 13 18
FIGURE S 14 19
FIGURE S 15 20
FIGURE S 16 21
FIGURE S 17 21
FIGURE S 18 22
FIGURE S 19 23
FIGURE S 20 23
FIGURE S 21 24
FIGURE S 22 26
FIGURE S 23 27
FIGURE S 24 27
FIGURE S 25 28
FIGURE S 26 29
FIGURE S 27 31
FIGURE S 28 32
FIGURE S 42 41
FIGURE S 43 42
FIGURE S 62 52
FIGURE S 63 53
FIGURE S 65 55
FIGURE S 66 57
FIGURE S 67 58
FIGURE S 68 59
FIGURE S 69 59
FIGURE S 70 60
FIGURE S 71 60
FIGURE S 72 61
FIGURE S 73 61
FIGURE S 74 62
FIGURE S 75 64
FIGURE S 76 64
FIGURE S 77 66
FIGURE S 78 66
FIGURE S 79 69
FIGURE S 80 69

1. Supporting data for the Introduction section

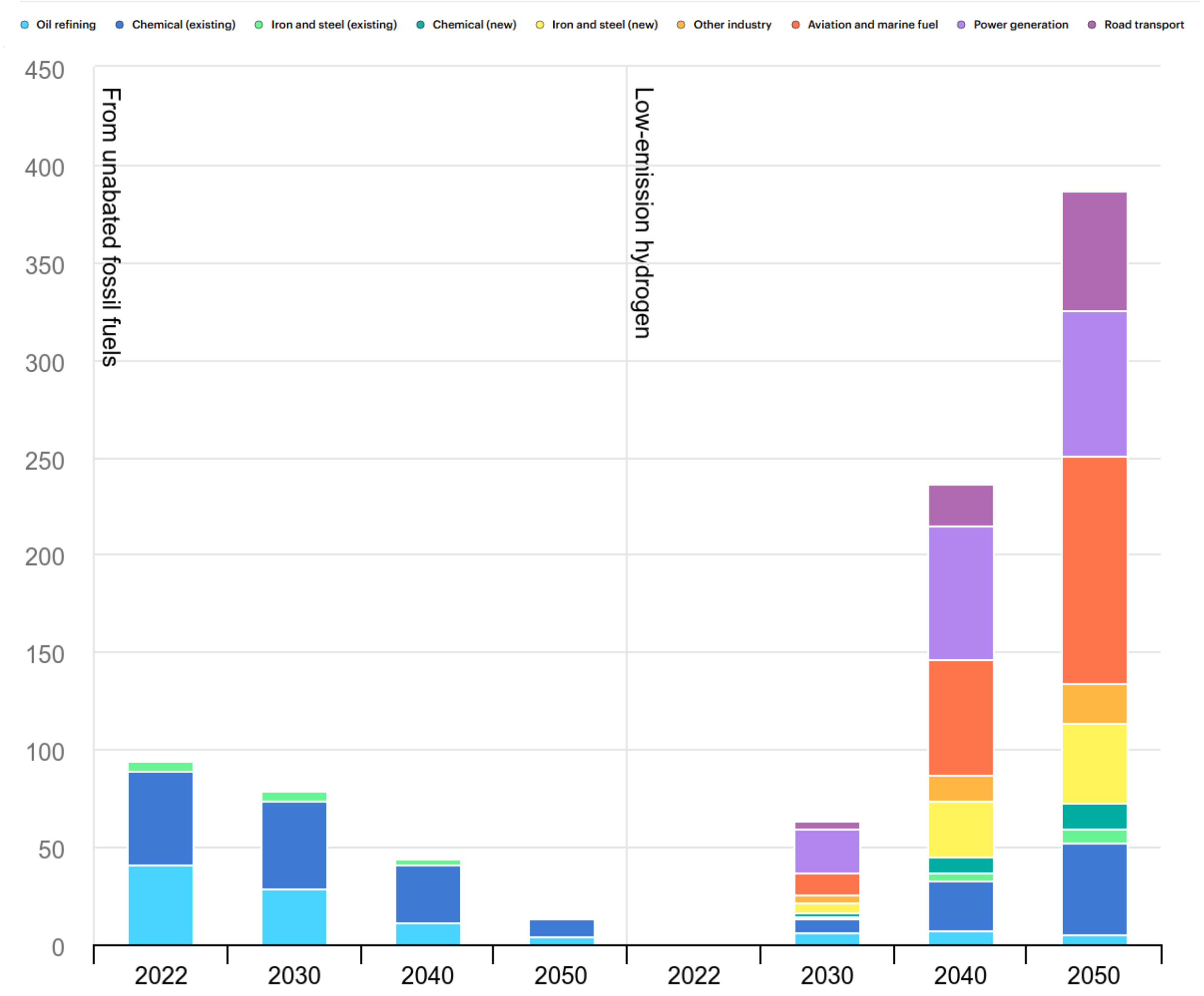

Notes. Mt H_2 = million tons of hydrogen. Unabated fossil fuels include hydrogen produced with CO_2 capture for utilization without storage, such as in urea synthesis. Demand for aviation and marine fuel and power generation includes hydrogen that is converted to make low-emissions hydrogen-based fuels.

Figure S 1. Source: IEA (2023), Global hydrogen demand in the Net Zero Scenario, 2022-2050, IEA, Paris https://www.iea.org/data-and-statistics/charts/global-hydrogen-demand-in-the-net-zero-scenario-2022-2050, Licence: CC BY 4.0.

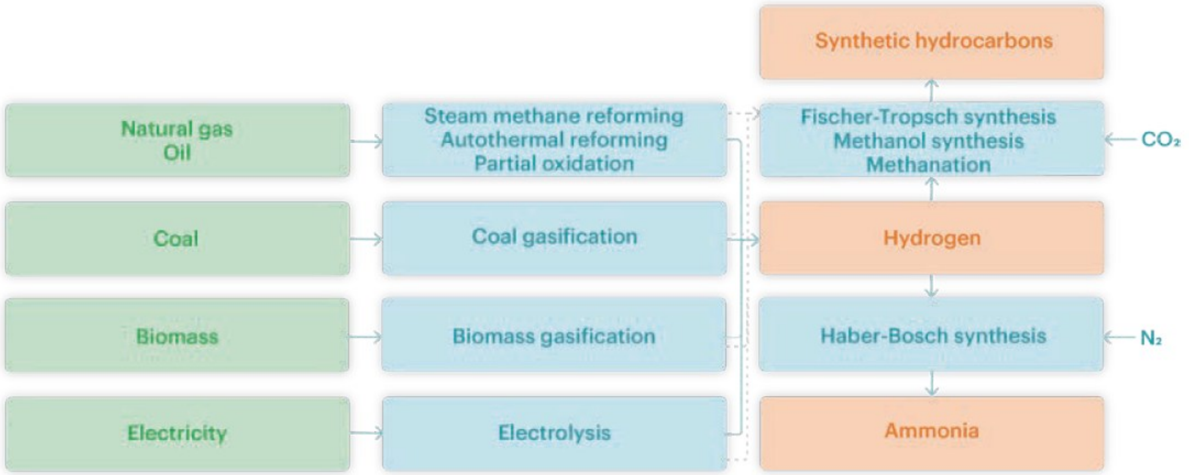

Notes: N_2 = nitrogen. The dotted lines represent the flow of hydrogen-containing synthesis gas (mixture of hydrogen and carbon monoxide) from hydrocarbon fuels for further conversion into other synthetic hydrocarbons, such as coal-to-liquids or gas-to-liquids. Though not discussed in this chapter, this direct conversion route of hydrocarbons via synthesis gas into other synthetic hydrocarbons is likely more favourable in terms of emissions (especially when coupled with CCUS) or costs compared with producing pure hydrogen from hydrocarbons first and then combining this hydrogen again with CO_2 for the production of synthetic hydrocarbons, particularly if the CO_2 input is of fossil origin.

Source: IEA 2019. All rights reserved.

Figure S 2. IEA (2019), The Future of Hydrogen, IEA, Paris https://www.iea.org/reports/the-future-of-hydrogen, Licence: CC BY 4.0.

Table S 1. Dipolar Aprotic Solvents Arranged by Current Understanding of Reprotoxicity. Reprinted from J. Sherwood, T. J. Farmer, J. H. Clark. Catalyst: Possible Consequences of the N-Methyl Pyrrolidone REACH Restriction. Chem **2018**, 4, 2010-2012, Copyright (2025), with permission from Elsevier.

Table 2. Dipolar Aprotic Solvents Arranged by Current Understanding of Reprotoxicity

Current Understanding of Reprotoxicity[a]	Dipolar Aprotic Solvents
No evidence of reprotoxicity (OEDC test method)	acetonitrile (OECD 422),[b] dimethyl sulphoxide (OECD 421), NBP (OECD 422), triethyl phosphate (OECD 407–408), and tributyl phosphate (OECD 416)[c]
No direct test results available	GVL, Cyrene, ethylene carbonate,[d] and propylene carbonate[d]
Suspected of damaging fertility or the unborn child	DMEU and DMPU
Can damage fertility or the unborn child	DMF, DMAc, NMP, NEP, sulpholane, and hexamethyl phosphoramide[e]

[a]From REACH dossiers where available.
[b]Acetonitrile can contain traces of acrylonitrile, which is a carcinogen.
[c]Suspected of causing cancer.
[d]Classification of cyclic carbonates as non-reprotoxic has been done on the basis of their rapidly formed metabolites (ethylene glycol and propylene glycol). In addition, chronic exposure to ethylene carbonate can cause kidney damage.
[e]Can cause cancer.

Table S 2. Comparison of the efficiency of our approach with previous metal-free and metal-based catalytic systems for the hydrolysis of Et_3SiH, Et_2SiH_2, $PhSiH_3$ and PMHS at 298 K.

$HSiR_3$	H_2O	Catalyst	Solvent	H_2 yield	Rate (mM/min)	TON[b]	TOF (min^{-1})	Comments	Source – Reference
**$PhSiH_3$ (1.11 mmol)**	**DI-H_2O (1.11 mmol)**	**KO^{t}Bu (5 mol%)**	**DMSO (0.5 mL)**	**99%[a]**	**1850.0×10^{-1}[c]**	**30.7**	**202 ± 5[c]**	- Minimum H_2O and DMSO (reduced waste)<br>- Outstanding activity (TOF)	**Figure 4 – This work**
PMHS (1.11 mmol)	DI-H_2O	DMSO	DMSO (0.5 mL)	99%[a]	15.6×10^{-1}[c]	30.7	43.8[c]		
Et_2SiH_2 (1.11 mmol)	(1.11 mmol)	(0.5 mL)	DMSO (0.5 mL)	99%[a]	14.8×10^{-1}[c]	30.7	40.9[c]		
Et_3SiH (1.11 mmol)	DI-H_2O	DMSO	DMSO (0.5 mL)	96%[a]	1.07×10^{-1}[c]	28.8	2.88		
**PMHS (1 mmol)**	**DI-H_2O (30 mmol)**	**KOH (5 mol%)**	**THF (4.4 mL)**	**99%**	**5.93×10^{-2}**	**200**	**8[c]**	- Large excess of water and THF (waste)<br>- Low catalytic activity (TOF)<br>- Not compatible with alkyl silanes	Figure 1 in ref. [1]
$PhSiH_3$ (1 mmol)	DI-H_2O (30 mmol)	KOH (5 mol%)	THF (4.4 mL)	99%	5.93×10^{-2}	8.4	0.28[c]		
E_3SiH (1 mmol)	DI-H_2O (30 mmol)	KOH (5 mol%)	THF (4.4 mL)	0%	--	--	--		
**$PhSiH_3$ (3.7 mmol)**	**DI-H_2O (0.2 mL)**	**HMPA (1.5 mol%) + NaOH (30 mol%)**	**H_2O (0.2 mL)**	**99%[d]**	**61.0×10^{-1}**	**3.17**	**5.3[c]**	- Highly toxic HMPA<br>- Large excess of NaOH as co-catalyst (30 mol%)	Table 1 in ref. [2]
PMHS (3.7 mmol)	DI-H_2O (0.2 mL)	HMPA (1.5 mol%)	H_2O (0.2 mL) + NaOH (30% mol)	85%[d]	52.0×10^{-1}	2.7	4.5[c]		
$PhSiH_3$ (1 mmol)	DI-H_2O (11.11 mmol)	**--[e]**	**DMPU (0.8 mL)**	**99%**	**1.49×10^{-1}**	**0.15**	**0.022[c]**	- DMPU = reprotoxic<br>- Large excess of H_2O with respect to hydrosilane<br>- Very low activity (TOF)	Table S7/8 in ref [3]
E_3SiH (1 mmol)	DI-H_2O (5.55 mmol)	--[e]	DMPU (0.8 mL)	82%	9.71×10^{-2}	0.12	0.020		
PMHS (1 mmol)	DI-H_2O (5.55 mmol)	--[e]	DMPU (0.9 mL)	85%	1.13×10^{-1}	0.11	0.012[c]		

[a] Based on the liberation of the first equivalent of H_2 from H_2O. [b] Mol of H_2 per mol of catalyst. [c] Calculated from the liberation of the first equivalent of H_2 from H_2O. [d] Determined after a reaction time of 10 s. [e] The solvent was used as the catalyst.

$HSiR_3$	H_2O	Catalyst	Solvent	H_2 yield	Rate (mM/min)	TON[b]	TOF (min^{-1})	Comments	Reference
**$PhSiH_3$ (0.053 mmol)**	**DI-H_2O (1.59 mmol)**	**Rhenium complex (1 mol%) + trityl-tetra(pentaflourophenyl) borate (1 mol%) [f]**	**MeCN (1.6 mL)**	**66%**	**--**	**15.6**	**15.6[g][c]**	Transition metal + boron additive = expensive<br>- Large excess of H_2O and metal-precatalyst/additive<br>- Moderate activity (TOF)	Table 1 in ref. [4]
E_2SiH_2 (0.053 mmol)	DI-H_2O (1.59 mmol)		MeCN (1.6 mL)	72%	--	17.0	17.0[g][c]		
E_3SiH (0.053 mmol)	DI-H_2O (1.59 mmol)		MeCN (1.25 mL)	97%	--	22.9	22.9[g]		
**E_3SiH (0.25 mmol)**	**DI-H_2O (2.5 mmol)**	**Iridium complex (1 mol%)**	**THF (1 mL)**	**99%**	**4.3×10^{-1}**	**99**	**170.0**	- Transition metal = expensive<br>- Large excess of H_2O<br>- Good to moderate activity (TOF)	Table 1 & Figure S12 in ref. [5]
$PhSiH_3$ (0.25 mmol)	DI-H_2O (2.5 mmol)	Iridium complex (1 mol%)	THF (1 mL)	99%	1.5×10^{-2}	99	5.9[c]		
$PhSiH_3$ (0.22 mmol)	DI-H_2O (2.2 mmol)	Iridium complex (0.2 mol%)	THF (1 mL)	99%	--	500	--[h]	- Transition metal = expensive and non-sustainable<br>- Large excess amount of H_2O with respect to hydrosilane<br>- Moderate activity (TOF)	Table 2 & Table S3 in ref. [6]
E_3SiH (0.22 mmol)	DI-H_2O (2.2 mmol)	Iridium complex (0.2 mol%)	THF (1 mL)	67%	--	335	--[h]		
**Et_2SiH_2 (0.22 mmol)**	**DI-H_2O (2.2 mmol)**	**Iridium complex (0.2 mol%)**	**THF (1 mL)**	**90%**	**8.8×10^{-3}**	**450**	**18[c]**		
**$PhSiH_3$ (5.67 mmol)**	**DI-H_2O (27.75 mmol)**	**Zinc complex (1 mol%)**	**THF (2 mL)**	**99%**	**29.0×10^{-1}**	**33.5**	**69**	- Ligand = complex/expensive<br>- Large excess amount of H_2O with respect to hydrosilane<br>- Good activity (TOF)	Page S3 in ref. [7]
**E_3SiH (2 mmol)**	**DI-H_2O (60.0 mmol)**	**$AgNO_3$ (1 mol%)**	**THF (6mL)**	**99%**	**6.7×10^{-2}**	**100**	**20**	- Transition metal = abundant but other critical demand<br>- Large excess amount of H_2O with respect to hydrosilane<br>- Moderate activity (TOF)	Table 2 in ref. [8]

[f] All catalytic runs were conducted at 293 K. [g] Calculated assuming a time of 1 minute for the liberation of the first equivalent of hydrogen for Et_3SiH or the first two equivalents of hydrogen of Et_2SiH_2 and $PhSiH_3$ (in the original paper, it was only mentioned the following statement about the speed of the reaction: '*Reactions reach completion (100% conversion) in less than 1 h*'). [h] In Table S3[6] of this study, the authors only reported the $TOF_{1/2}$ (h^{-1}) numbers at half-conversion; our comparison of kinetic/catalytic activity data holds only when made between systems describing TOFs at (near) full conversion.

2. General considerations

Chemicals and operational conditions

All solvents and reagents were purchased from Fluka, Aldrich or TCI unless otherwise specified. Hydrosilanes and DMSO were dried by passing over a short column of basic Al_2O_3, degassed by nitrogen bubbling and stored over activated molecular sieves (3 Å) under an inert atmosphere. PMHS was purchased from Sigma-Aldrich (product #176206-250G; average Mn 1700-3200). All other solvents were degassed by nitrogen bubbling and stored over activated molecular sieves (3 Å) inside the nitrogen-filled glovebox. KO*t*Bu was maintained under moisture-free atmosphere inside an evacuated-desiccator. All experiments involving the latter chemicals were conducted under standard conditions of temperature and pressure without strict exclusion of air, unless otherwise stated. The Schlenk tube used for the reaction was thoroughly dried with a heat gun then with then oven (100 °C) prior each measurement. All chemicals were added to the Schlenk tube while maintaining a strict nitrogen atmosphere, except for the hydrosilane which was quickly added while the side arm of the Schlenk tube was open to atmospheric pressure of the inverted burette.

Analytics

NMR spectra were recorded on a Bruker AVANCE III 300 spectrometer (^{1}H NMR 300.13 MHz, ^{13}C NMR 75.47 MHz, ^{29}Si NMR 59.63 MHz). Chemical shifts are given in ppm. ^{1}H NMR and ^{13}C NMR spectra were referenced using the solvent signals (^{1}H: residual $C\mathit{H}Cl_3$ ($CDCl_3$) = 7.26 ppm, residual $(C\mathit{H}D_2)_2SO$ = 2.50 ppm; ^{13}C: $\mathit{C}DCl_3$ = 77.16 ppm, residual $(\mathit{C}HD_2)_2SO$ = 39.52 ppm). When necessary, ^{29}Si NMR spectra were calibrated against the *internal* standard $SiMe_4$ = 0 ppm (added to the mixture prior the measurement). Data are reported as follows: Chemical shift in ppm, multiplicity (b = broad, s = singlet, d = doublet, t = triplet, q = quartet, m = multiplet, dd = doublet of doublets, etc.), coupling constant *J* in Hz, integration, and (where applicable) interpretation. Head-space gas chromatography (GC) using FID, TCD detectors and GC-MS (ISQ761-single quadrupole) measurements were recorded using Thermo-Fischer Trace 1610 instruments. For the acquisition of infrared spectra, we used an Agilent Cary 630 spectrometer equipped with a solid-state probe. EPR spectra were recorded by a Magnettech Miniscope MS benchtop X-band ESR spectrometer, Germany.

Computational details

All density functional theory calculations were performed with TURBOMOLE 7.9.[9-11]

For all geometry optimizations and calculations of vibrational frequencies, the B3LYP hybrid functional (grid size m5) was used together with the def2-SVPD basis set and the D3BJ dispersion correction.[12-20] Additionally, to increase the accuracy of the electronic energies, single points were calculated at the B3LYP-D3BJ/def2-TZVPPD@B3LYP-D3BJ/def2-SVPD level of theory.[16] To account for solvent effects, the COSMO-GCM model (Lebedev grid 7) was employed for all calculations (DMSO, ε_r = 46.7).[21,22] Additionally, the resolution of identity approximation (RIJK) was utilized throughout.[23–28]

Transition states were located by performing reaction path calculations with TURBOMOLE’s woelfling program and subsequent geometry optimization.[29] Analytical normal modes were calculated using TURBOMOLE’s aoforce program both for confirmation of the stationary points and to access thermochemical properties. The latter were obtained *via* the rigid rotor harmonic oscillator (RRHO) approximation at the B3LYP-D3BJ/def2-SVPD level of theory (298.15 K).

Rendered images of the 3D-structures were generated with Jmol.[30]

3. Representative procedure for hydrogen generation

General procedure for hydrogen generation (example is given with $HSiEt_3$)

In a dry Schlenk tube under nitrogen atmosphere was first added 20 µL of deionized (DI) water (1.11 mmol) and KO*t*Bu (6.2 mg; 0.055 mmol) which are both taken from a pre-mixed stocked solution of DI water/KO*t*Bu mixture. Then dry 0.5 mL DMSO was added. The Schlenk tube was sealed under nitrogen atmosphere with a screw-thread cap equipped with a PTFE septum for injection. Finally, 129 mg of dry $HSiEt_3$ (1.11 mmol) was quickly added with a syringe equipped with a long needle that penetrates smoothly through the PTFE septum until it reaches the top of the stirred reaction mixture. Just few seconds before injection of the silane, the side-arm of the Schlenk tube was open to the inverted burette for hydrogen collection and measurement (see Figure S3).

While the PMHS utilized in our standardized laboratory studies was obtained from commercial sources to ensure kinetic reproducibility, PMHS on an industrial scale represents a massive, underutilized waste by-product of the Müller-Rochow process.

Based on documented industrial data, the global production of silanes via the Müller-Rochow direct process is on the order of 5 to 8 million metric tons annually[31] (Rösch, L., John, P. and Reitmeier, R. *Silicones*. In *Ullmann's Encyclopedia of Industrial Chemistry* (Wiley-VCH)). The methyldichlorosilane ($MeHSiCl_2$) byproduct typically constitutes 1% to 5% of the crude monomer stream. This results in approximately 50,000 to 100,000 metric tons of PMHS precursor generated globally per year. Applying our catalytic hydrolysis method, the stoichiometric extraction of hydrogen from the PMHS repeating unit ([CH_3SiHO], MW approx. 60.1 g/mol) yields one mole of H_2 per mole of monomer. Therefore, 1 kilogram of PMHS theoretically yields approximately 16.6 moles of H_2 (a gravimetric capacity of ~3.35 wt%, or roughly 370 liters of H_2 gas at standard temperature and pressure). Extrapolating this to the global scale, and entirely independent of any downstream silicon recycling, our method could theoretically generate 1,600 to 3,300 metric tons of high-purity hydrogen gas annually from this single, underutilized waste stream alone.

Setup for hydrogen evolution measurements

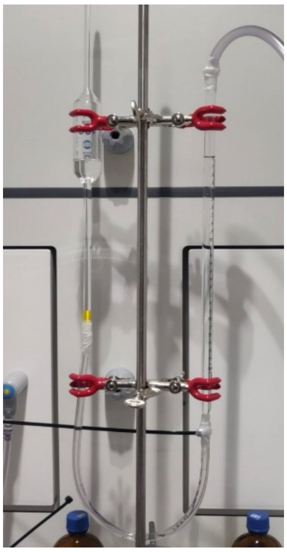

Figure S 3. Description of the inverted water-filled burette system for H_2 measurements.

4. Catalysis development

Solvent effect –initial H_2 formation rates as a function of solvent dielectric constant

Table S 3. Initial rates obtained for the silane hydrolysis reaction in different solvents.

Solvent	Initial reaction rate (mM/min)[a]	Dielectric constant (ε_r^T)[b]	
**H_2O**	5.10×10^{-3}	80.100^{20}	***Increased ε_r***
**Dimethyl sulfoxide**	1.02×10^{-1}	47.24^{20}	
**Acetonitrile**	1.27×10^{-2}	36.64^{20}	
**Tetrahydrofuran**	1.18×10^{-2}	7.52^{22}	
**Butyl acetate**	4.10×10^{-3}	5.07^{20}	
**Toluene**	4.60×10^{-3}	2.379^{23}	
**Cyclohexane**	6.35×10^{-3}	2.0243^{20}	
**No solvent**	2.80×10^{-3}	--	

[a]Derived from the first 10 min. of the reaction. [b]Dielectric constant of the solvent measured at temperature T/°C. From: CRC Handbook of Chemistry and Physics (92th ed.)

Reported pK_b values of the tested base catalysts in DMSO

Table S 4. Measured and estimated pK_b values of the conjugate acids for various bases in DMSO.

Molecule	pK_b (DMSO)	Ref.
DMSO (dimsyl anion)	35.0	32
H_2O (OH^-)	31.4	33
KOMe	29.0	33
KOH	31.4	33
KO*t*Bu	32.2	33
NaOAc	12.6	34
NaOH	31.4	33
K_2CO_3	~20	35
NaOPiv	~12.5	35

Hydrogen evolution profiles for varying catalyst (KOtBu) loading

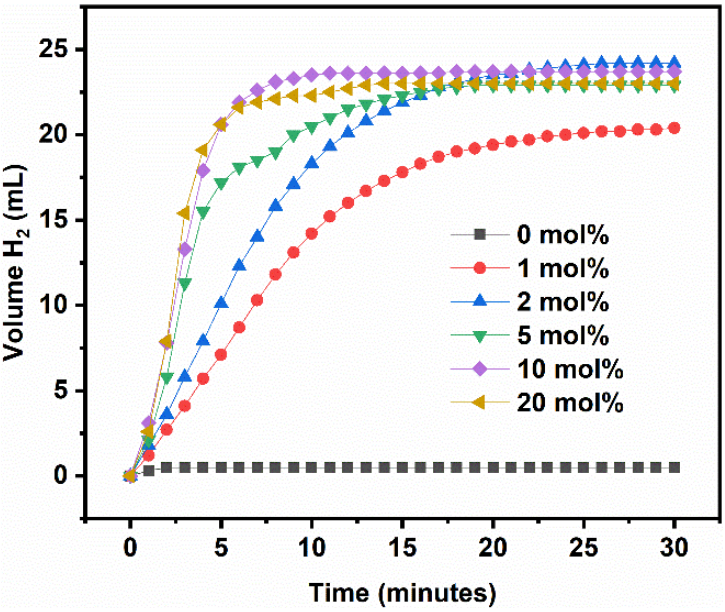

Figure S 4. The effect of different catalysts + control experiment without any added metal-free catalyst on the volume of hydrogen generated from the reaction of H_2O and $HSiEt_3$. Conditions: $HSiEt_3$ (129 mg, 1.11 mmol), H_2O (0.02 mL, 1.11 mmol), KO*t*Bu (6.2 mg, 0.055 mmol), DMSO (0.5 mL). The maximum theoretical V_{H2} that can be released from 1.11 mmol H_2O is ≈ 24.5 mL.

To shed light on the role of KO*t*Bu in this catalytic system, the influence of its concentration on the reaction rate was assessed by measuring the rate of hydrogen evolved over time. The resulting data are compared on the graph depicted in Figure S4. When the model reaction was conducted in the absence of KO^{t}Bu, no hydrogen generation occurred, as expected. Whereas increasing the loading of KO*t*Bu led to an increase of the reaction rate (Figure S4, Table S5 & Figure S5). Specifically, while using 1 mol% catalyst led to only 18% H_2 yield within 30 minutes, 95% H_2 yield was obtained in 15 minutes with 20 mol% loading. As shown in Figure S5, it seems that the rate dependence on catalyst loading does not follow a continuum. Indeed, the steady increase of the rate with catalyst loading suddenly breaks at the 5 mol% loading at which point a stagnation occurs in the higher loading regime. A viable explanation to this phenomenon is the observation of partially *insoluble* KO*t*Bu in the reaction mixture. Thus, it is the decreased solubility of KO*t*Bu in the DMSO/H_2O mixture with increased concentrations, reaching a saturation at ca. 10 mol%/0.5 mL, that limits the amount of active catalyst in solution which consequently results in a saturation kinetics behavior even at higher loading.

Initial H_2 formation rates data for varying catalyst (KOtBu) loading

Table S 5. Initial rates obtained for the silane hydrolysis reaction using different concentrations of the catalyst KOtBu.[a]

Equivalent KO*t*Bu (mol%)[b]	Initial reaction rate (mM/min)[c]
0.0 (0.0)	5.10×10^{-3}
0.01 (1.0)	1.29×10^{-1}
0.02 (2.0)	1.66×10^{-1}
0.05 (5.0)	1.89×10^{-1}
0.1 (10.0)	2.13×10^{-1}
0.2 (20.0)	2.05×10^{-1}

[a]The model reaction $HSiEt_3$ (1.1 mmol, 1 equiv.) + DI H_2O (1.1 mmol, 1 equiv.) was used for this study. [b]Relative to H_2O and $HSiEt_3$. [c]Derived from the first 10 minutes of the reaction.

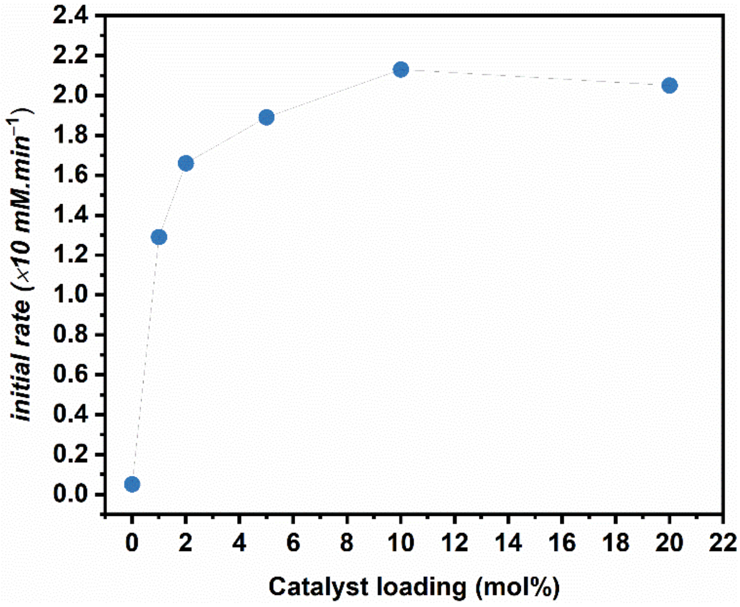

Figure S 5. Initial rates as a function of catalyst (KO*t*Bu) loading.

Kinetic Measurements of $PhSiH_3$ Hydrolysis

For the highly reactive silane $PhSiH_3$ yielding rapid gas evolution (<25 seconds), manual burette readings are insufficiently accurate. In these instances, the inverted burette was recorded using a high-definition camera in slow-motion mode. The exact volume of displaced water was extracted via frame-by-frame video analysis. All such hyper-fast experiments were performed in independent triplicates, with standard deviations calculated to ensure measurement fidelity (Figure S6). A representative video is provided as Movie S1.

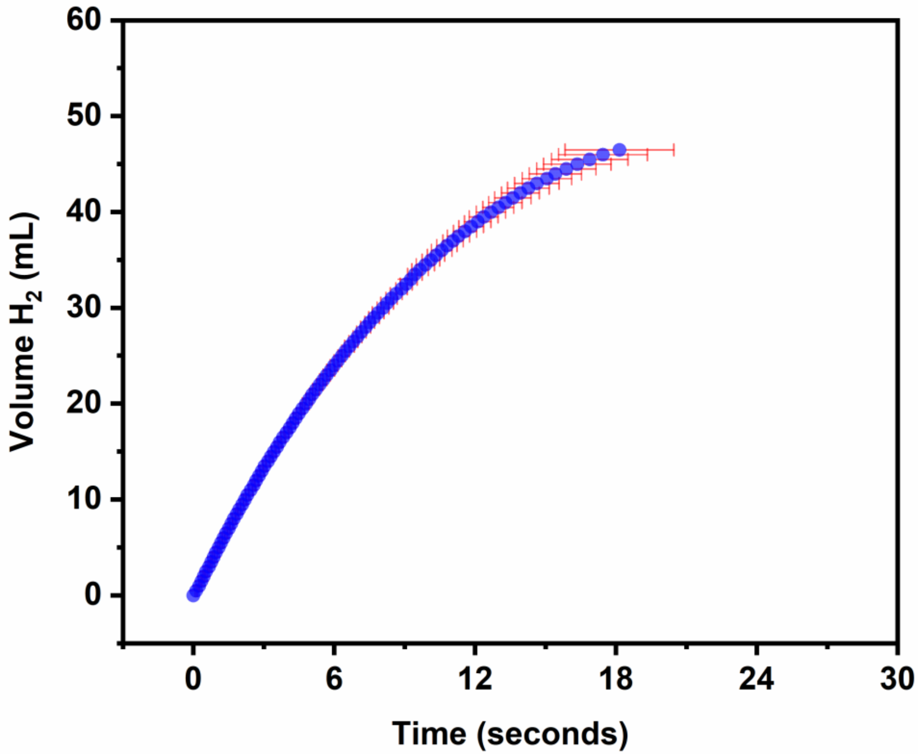

Figure S 6. High-resolution volume-time profile demonstrating the hyper-fast hydrogen evolution from the catalytic hydrolysis of PhSiH3 (1.11 mmol) at room temperature (5 mol% KOtBu, 0.5 mL DMSO). Horizontal error bars indicate the temporal standard deviation across independent triplicate measurements. See video S1.

5. NMR and GC analyses of the catalysis mixture

Experimental Procedure

Product identification

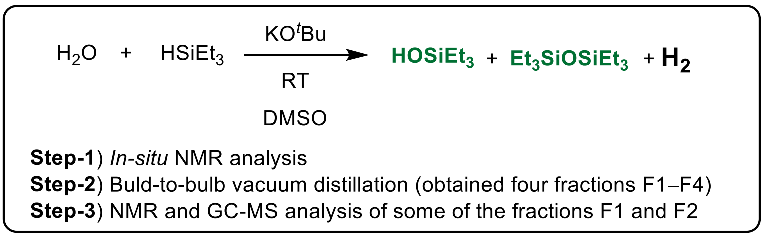

In a dry vial equipped with a screw-thread cap and PTFE septum was first added 20 µL of deionized (DI) water (1.1 mmol) and KO*t*Bu (8 mg; 7.13 µmol) which are both taken from a pre-mixed stocked solution of DI water/ KO*t*Bu mixture. Then dry 0.6 mL DMSO-d_6 was added. Stirring of the reaction mixture was vigorous (700 RPM) and began before the addition of $HSiEt_3$. Finally, 445 mg (3.83 mmol) of dry $HSiEt_3$ was quickly added with a syringe equipped with a long needle that penetrates smoothly through the PTFE septum until it reaches the top of the stirred reaction mixture. After about 30 minutes of stirring at RT, the reaction sample was transferred to an NMR sample tube and submitted for ^{1}H and ^{29}Si NMR analysis (**Step-1** in Figure S7). Then, the latter reaction sample was submitted to bulb-to-bulb distillation under vacuum for the purpose of isolating each of the silicon-based by-products (**Step-2** in Figure S8). The recovered fractions **F1** and **F2** were analyzed by NMR spectroscopy and GC-MS (**Step-3** in Figures S8–S11).

Step-1) In situ monitoring of the reaction mixture

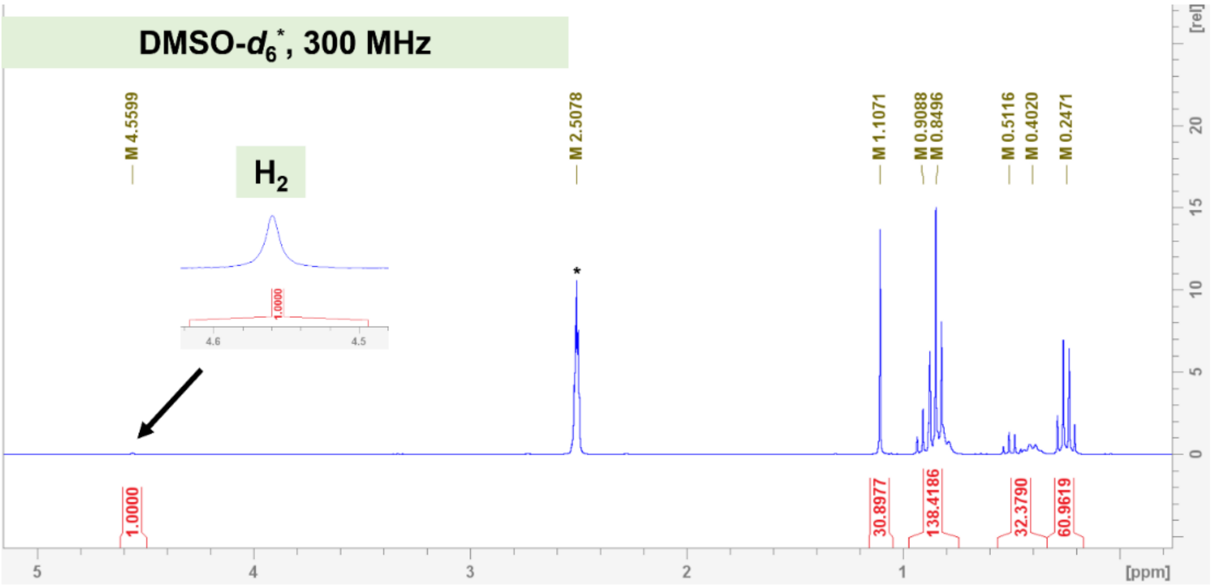

Figure S 7. Step 1: In situ monitoring of the catalysis mixture via ^{1}H NMR spectroscopy (300 MHz, 298 K). **Note**: The singlet peak at 1.1071 ppm is attributed to KO*t*Bu: The high concentration detected by NMR is due to its high solubility in DMSO. The relatively lower concentration of H_2 detected by NMR is due to its very limited solubility in DMSO (mostly in the gas phase).

Step-3) Analysis of the distilled fraction F1

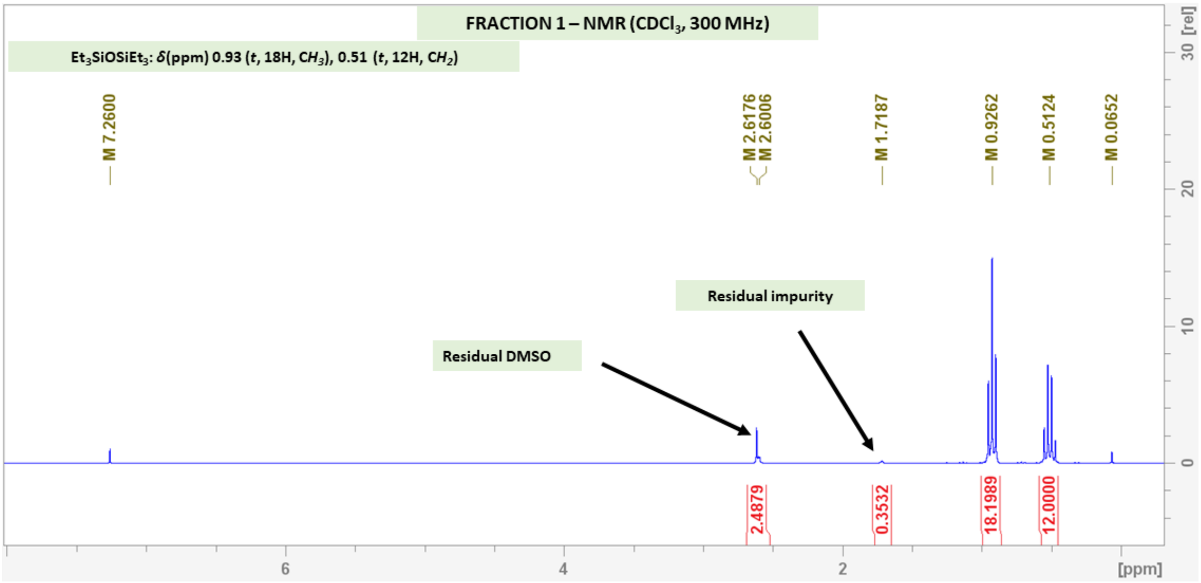

Figure S 8. Steps 2/3: Distillation of the catalysis mixture (top) followed by analysis the first isolated fraction (F1) via ^{1}H NMR spectroscopy (300 MHz, 298 K) (bottom).

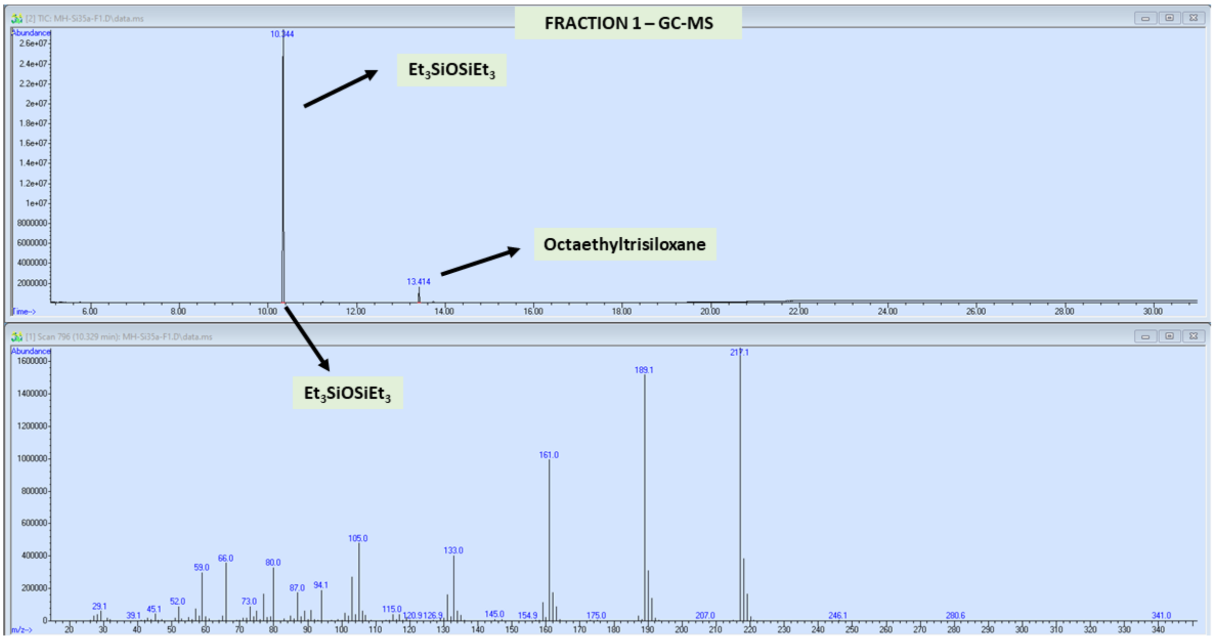

Figure S 9. Steps 2/3: Distillation of the catalysis mixture (top) followed by analysis the first isolated fraction (F1) via GC-MS (bottom).

Step-3) Analysis of the distilled fraction F2

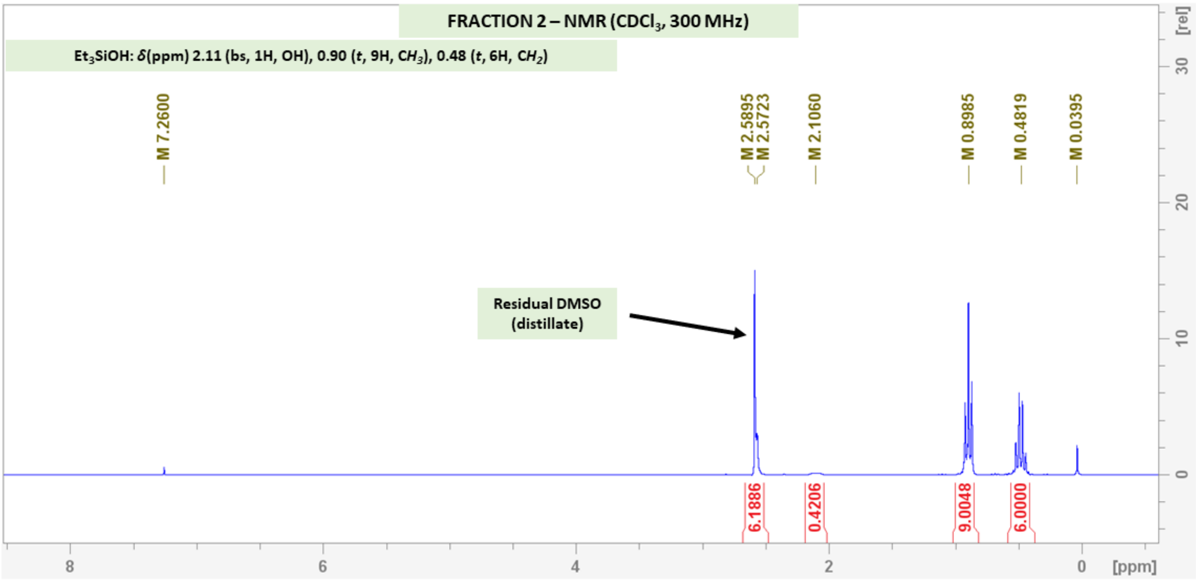

Figure S 10. Steps 2/3: Distillation of the catalysis mixture (top) followed by analysis the second isolated fraction (F2) via ^{1}H NMR spectroscopy (300 MHz, 298 K) (bottom).

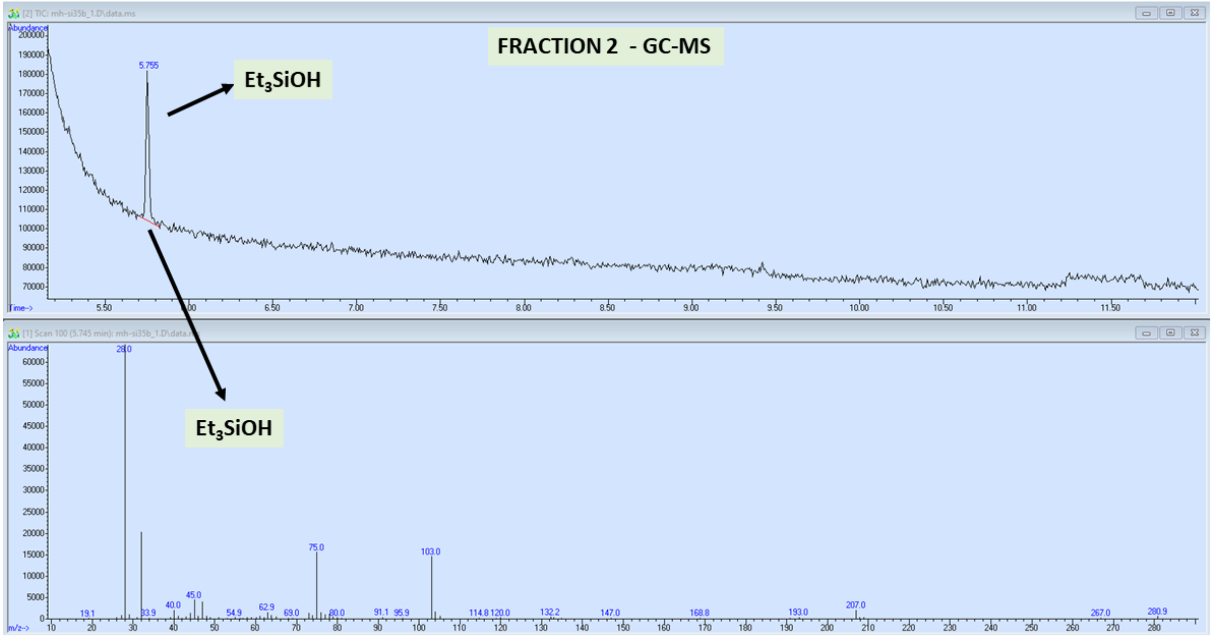

Figure S 11. Steps 2/3: Distillation of the catalysis mixture (top) followed by analysis the second isolated fraction (F2) via GC-MS (bottom).

To conclude, in situ NMR analysis of the reaction mixture led to the observation of H_2 alongside those attributed to the Si by-products ($Et_3SiOSiEt_3$ and $HOSiEt_3$). Whereas subsequent distillation of the mixture lead to the isolation of two distinct fractions, F1 and F2. NMR analysis and GC analysis of F1 revealed the formation of $Et_3SiOSiEt_3$ as the main species while analysis of F2 concluded that Et_3SiOH was formed during the reaction, although in smaller amount.

6. Hydrogen detection and controls

Hydrogen detection by in situ NMR spectroscopy

Procedure. Using the general conditions, inside a head space vial (hereafter named '**vial-A**')) was first added 100 µL of DI water (5.55 mmol) and KO*t*Bu (11.8 mg; 105.5 µmol). Then dry 0.5 mL DMSO was added. **Vial-A** was sealed under air with a screw-thread cap equipped with a PTFE septum. **Vial-A** was connected through a stainless-steel cannula to another similar vial ('**vial-B**') containing ca. 1 mL toluene-d_8. Finally, 200.5 mg of dry $HSiEt_3$ (1.72 mmol) was quickly added with a syringe equipped with a long needle that penetrates smoothly through the PTFE septum of **vial-A** until it reaches the top of the stirred catalysis mixture. In order to saturate the solution of toluene-d_8 in **vial-B**, the hydrogen generated in the catalysis mixture of **vial-A** was bubbled into **vial-B** by immerging the septum ca. 0.2 cm inside the solution. The mixture was stirred at room temperature for ca. 30 minutes before a sample of toluene-d_8 was taken for ^{1}H NMR analysis using a *J*-Young tube. The resulting NMR spectrum is shown below (Figure S12).

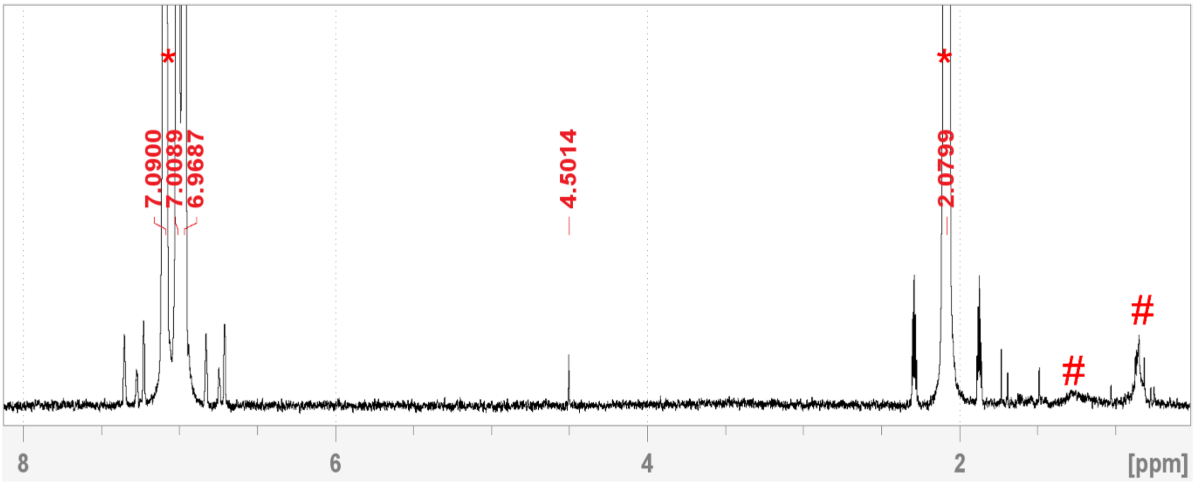

Figure S 12. ^{1}H NMR spectrum (toluene-d_8*) showing the hydrogen (peak at 4.50 ppm) that was generated from the silane hydrolysis mixture (**vial-A**) and that was directly bubbled into the toluene-d_8 solution contained in the second vial (**vial-B**). Oil impurity (#).

Hydrogen detection by gas chromatography (GC)

Procedure. Using the general conditions, inside a head space vial was first added 66 µL of DI water (3.66 mmol) and KO*t*Bu (20.6 mg; 183.18 µmol). Then dry 0.5 mL DMSO was added. The vial was sealed under air with a screw-thread cap equipped with a PTFE septum for injection. Finally, 426 mg of dry $HSiEt_3$ was quickly added with a syringe equipped with a long needle that penetrates smoothly through the PTFE septum until it reaches the top of the stirred reaction mixture. The mixture was stirred at room temperature for ca. 30 minutes before head-space injection into the GC.

TCD head space injection –gas chromatographic conditions

GC: ExplorisGC (Thermo Fisher Scientific).
Column flow (He): 5.0 mL/min.
Column: CP-Molsieve 5A 50 m x 0.32. mm x 30 mm, 5 in.
Run Time: 30.00 min.
Syringe temperature: 50.00 °C.
Injection Volume: 1000.00 mL.

Incubation (Agitator)temperature/ Incubation time: 40.000 °C/2.00 min.
TCD detector temperature: 250 °C.
Filament detector temperature: 300 °C.
Oven: a. 70 °C for 10 min.
b. 70-300°C at 23 °C/min, 300 °C for 10 m

TCD head space injection – the original report

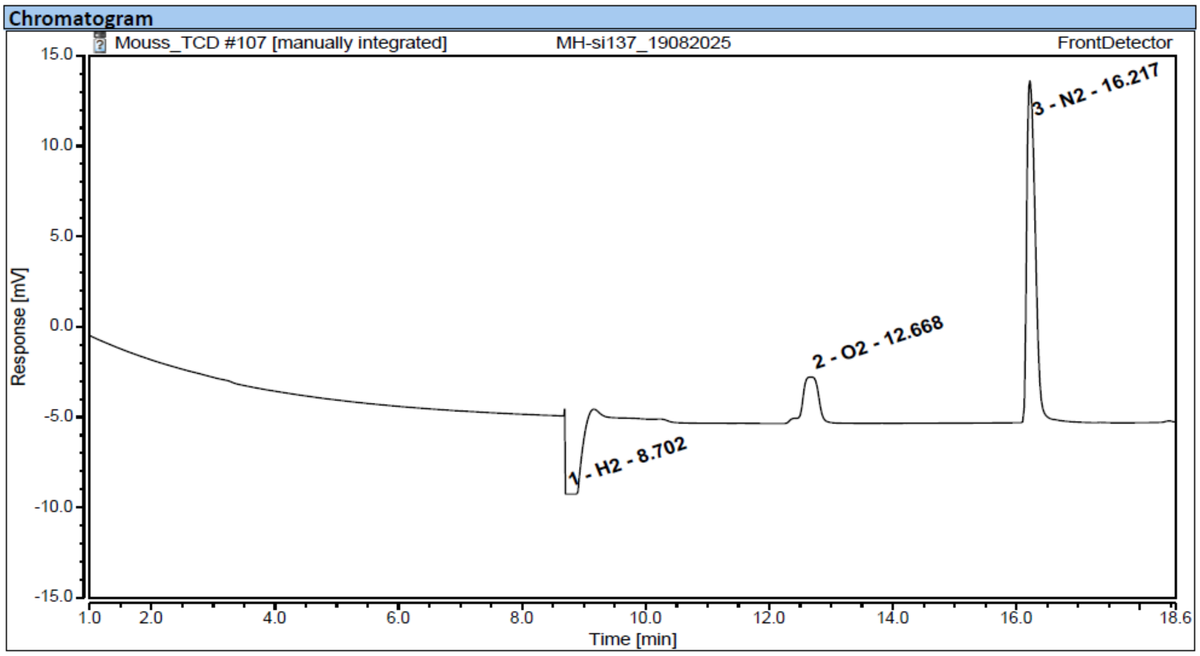

Figure S 13. GC-TCD chromatogram showing the hydrogen (peak with retention time at 8.7 minutes) that was generated from the catalytic mixture of hydrolysis of $HSiEt_3$.

Verification that hydrogen was generated, via indirect hydrogenation

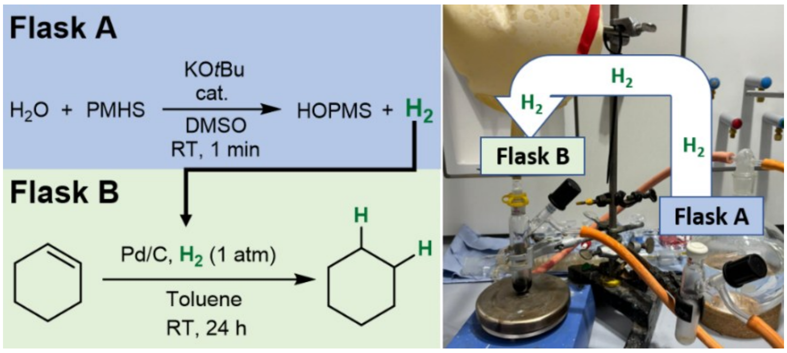

Figure S 14. The hydrogen generated from the silane hydrolysis (flask A) was directly used for the hydrogenation of cyclohexene (flask B) to cyclohexane (100% conversion; >99% yield). Conditions Flask A: PMHS (3 g, 50.45 mmol), H_2O (0.5 mL, 27.75 mmol), KO*t*Bu (155.75 mg, 1.375 mmol), DMSO (1 mL); conditions Flask B: cyclohexene (91.2 mg; 1.11 mmol), Pd/C-10 wt% (118 mg; 0.1108 mmol), toluene (2 mL). GC-MS analysis of the reaction sample in flask B revealed full conversion into cyclohexane (see spectrum below).

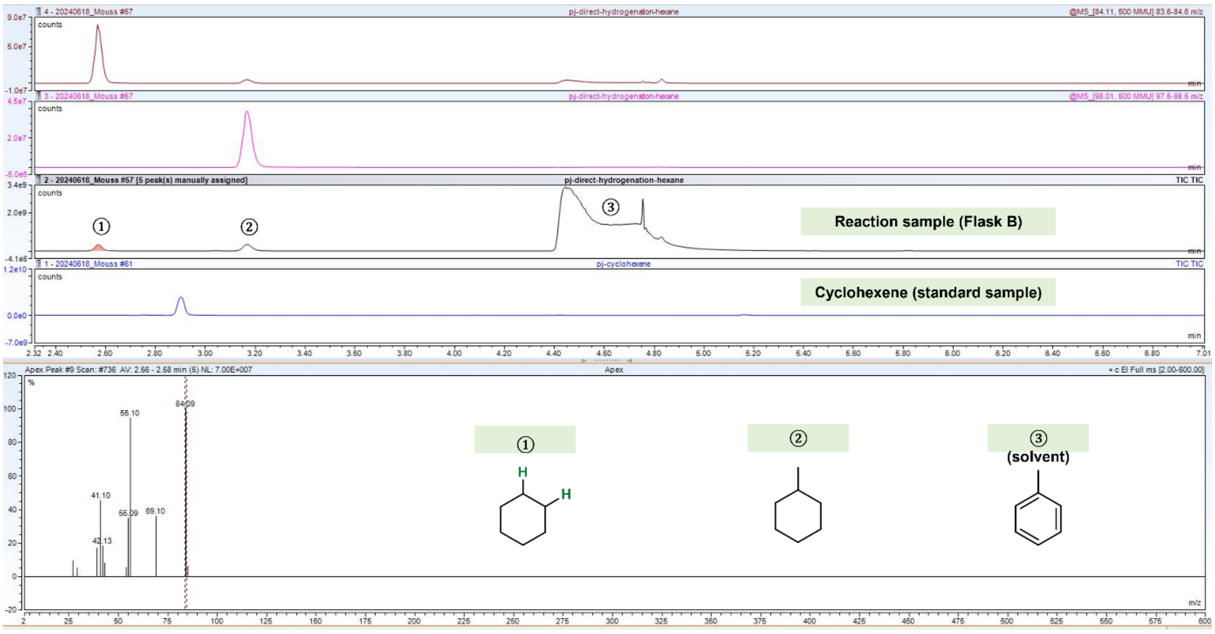

Figure S 15. Analysis of the cyclohexene hydrogenation catalysis mixture (Flask B; Figure S14) via GC-MS. The stacked chromatograms identify the characteristic peaks for cyclohexane (main hydrogenation product labelled #1) alongside peaks characterizing the byproduct methylcyclohexane (#2) and the solvent toluene (#3). Full conversion of cyclohexene to cyclohexane was observed.

Further control experiments exclude the hydrogen generation solely from DMSO and $HSiEt_3$

In order to check for the potential contribution of residual water content to the measured hydrogen volume in the above silane hydrosilane experiments, we have conducted a series of control experiments where either dried or non-dried $HSiEt_3$ was mixed with either dried or non-dried DMSO in the presence of KO*t*Bu and without the addition of water.

The results shown in Figure S16 demonstrate that when dry $HSiEt_3$ and dry DMSO were used, only *ca.* 0.5 mL H_2 was generated. In contrast, when non-dried $HSiEt_3$ was combined with non-dried DMSO, 14 mL H_2 was generated, which implies that a significant amount of water was present in both samples. These results are consistent with the water content measurements made for non-dried and dried samples of $HSiEt_3$ and DMSO using either ^{1}H NMR spectroscopy or Karl Fischer titration (Table S6). It is worth knowing that Karl Fisher titrations analysis of KO*t*Bu contains only *ca.* 1.7 wt% of H_2O, which is negligible in terms of hydrogen generation potential in comparison to the total volume of hydrogen generated under the standard conditions (*i.e.* 1.11 mmol H_2O versus 0.055 mmol KO*t*Bu).

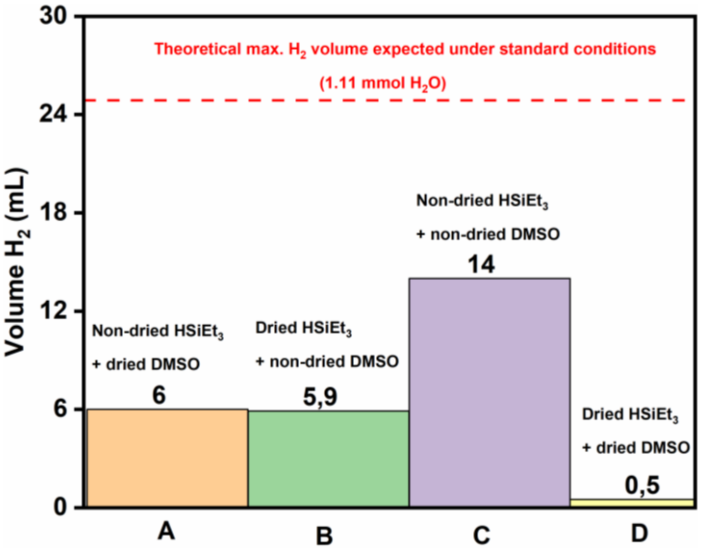

Figure S 16. Water-free control experiments (no water is added to these mixtures): comparison of the volume of hydrogen (y-axis) generated from combinations A, B, C or D of either dried or non-dried substances (x-axis). Conditions: $HSiEt_3$ (129 mg, 1.11 mmol), KOtBu (6.23 mg, 0.055 mmol), DMSO (0.5 mL). The maximum theoretical V_{H2} that can be released from each combination of substances can be deduced from Table S6 below.

Table S 6. Water content measurements for different sources of substances used in the hydrolysis of silanes. The theoretical volume of released H_2 is calculated based on the measured water content in each substance.

Source	Water content determination (method)	Theoretical V H_2 (mL)
$HSiEt_3$ - Not dry	1.89 mol%[a]	2.88[b]
$HSiEt_3$ - Dry	0.2 mol%[a]	0.30
DMSO – Not dry	1.046 wt% [c]	6.90
DMSO - Dry	0.045 wt% [c]	0.30
KO*t*Bu - Dry	0.0367 wt% [c]	0.002

[a]Average of three successive ^1H NMR measurements of the same sample using $CDCl_3$ capillary. [b]This theoretical volume is lower compared to the experimentally measured one (ca. 6 mL), which is attributed to the low miscibility of H_2O in $HSiEt_3$. [c]Karl Fischer titration.

In addition, we have verified the potential presence of SMe_2 in the catalysis mixture which could form by the deoxygenation of DMSO in the presence of $HSiEt_3$. Careful analysis of the reaction mixture using ^1H/^{13}C NMR and GC-FID and comparison with authentic samples showed no evidence for the formation of SMe_2[36], thus ruling out the DMSO deoxygenation pathway (Figures S17–S19; cf. mechanism in Figure S21).

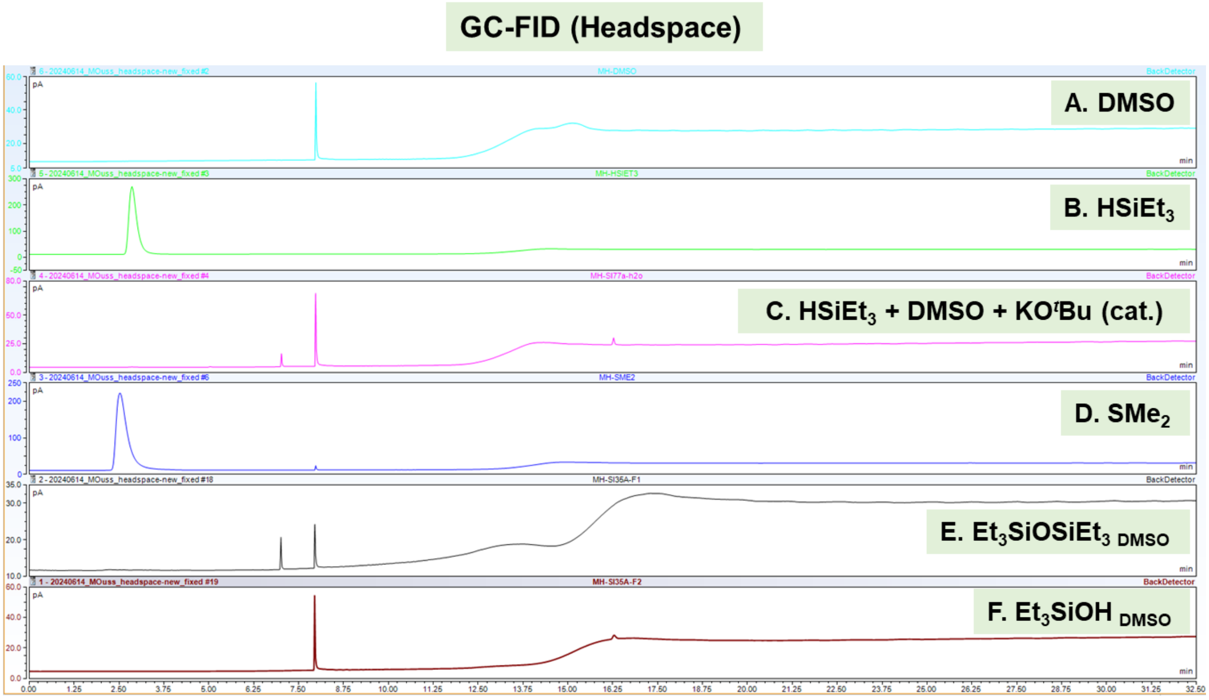

Figure S 17. Gas chromatography analysis (flame ionization detector (FID)) of the reaction mixture of silane hydrolysis and comparison to authentic (standard) samples using 20-mL headspace vials. **A.** DMSO (same sample as that used for catalysis studies). **B.** $HSiEt_3$ (same sample as that used for catalysis studies). **C**. Reaction mixture: DI-H_2O (50 µL, 2.78 mmol), $HSiEt_3$ (320 mg, 2.75 mmol), DMSO (0.25 mL), KO*t*Bu (17 mg, 0.152 mmol). **D**. SMe_2 (authentic sample). **E**. $Et_3SiOSiEt_3$ (authentic sample) in DMSO. **F**. Et_3SiOH (authentic sample) in DMSO.

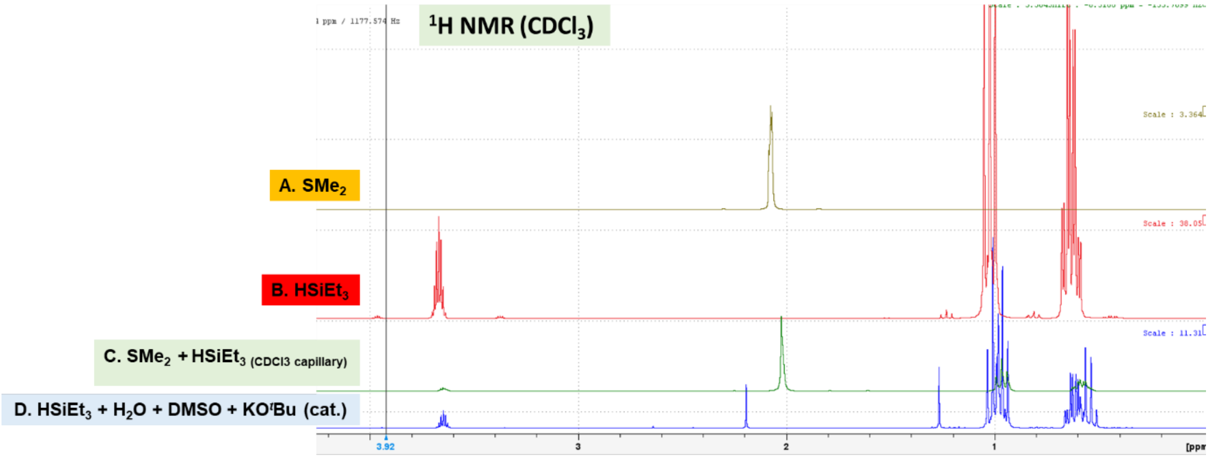

Figure S 18. ^{1}H NMR spectroscopy analysis ($CDCl_3$) of the reaction mixture of silane hydrolysis and comparison to authentic (standard) samples using a 300 MHz spectrometer at 293 K. **A**. SMe_2 (authentic sample). **B**. $HSiEt_3$ (same sample as the one used for catalysis studies). **C**. SMe_2 mixed with $HSiEt_3$ (same sample as that used for catalysis studies). **D**. Reaction mixture: DI-H_2O (50 µL, 2.78 mmol), $HSiEt_3$ (320 mg, 2.75 mmol), DMSO (0.25 mL), KO*t*Bu (17 mg, 0.152 mmol).

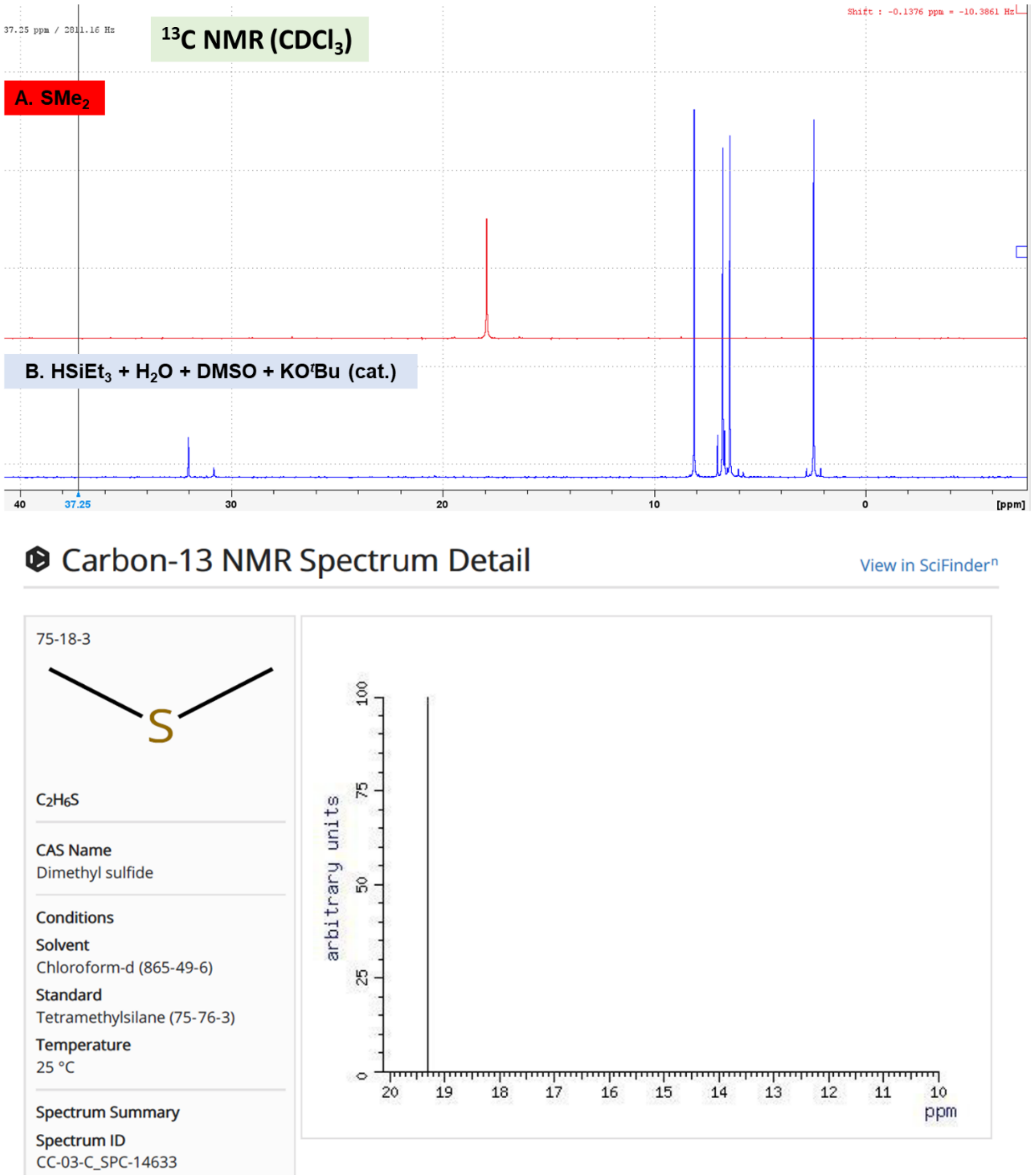

Figure S 19. TOP. ^{13}C NMR spectroscopy analysis ($CDCl_3$) of the reaction mixture of silane hydrolysis and comparison to authentic (standard) samples using a 300 MHz spectrometer at 293 K. **A**. SMe_2 (authentic sample). **B**. Reaction mixture: DI-H_2O (50 µL, 2.78 mmol), $HSiEt_3$ (320 mg, 2.75 mmol), DMSO (0.25 mL), KO*t*Bu (17 mg, 0.152 mmol). **BOTTOM.** ^{13}C NMR spectrum of SMe_2 in $CDCl_3$ (SciFinder database).

Labelling experiments exclude oxygen incorporation from DMSO into the Si byproducts

In order to verify whether DMSO is involved in oxygen (transfer) incorporation into the Si byproducts, a labelling experiment using $H_2{}^{18}O$ (99% ^{18}O) in place of $H_2{}^{16}O$ (main O-isotope in H_2O) was conducted under standard catalysis conditions (see general procedure for silane hydrolysis). After the evolution of hydrogen has ceased, a sample was submitted to GC-MS analysis. The results do not support the involvement of DMSO in the oxygen-transfer since the two isotopically enriched $H^{18}OSiEt_3$ and $Et_3Si^{18}OSiEt_3$ were the main Si-byproducts observed in the GC-MS; see the original GC-MS report in Figure S19 and the proposed mechanism for DMSO deoxygenation in Figure S20.

Note: About 30% of ^{16}O isotopically labelled forms of both Si byproducts was detected, which is attributed to the contribution of moisture mainly from DMSO. The detection of $H^{18}OSiEt_3$ provides strong evidence for the involvement of $H_2{}^{18}O$ in the oxygen incorporation (see Figures S20–S21.)

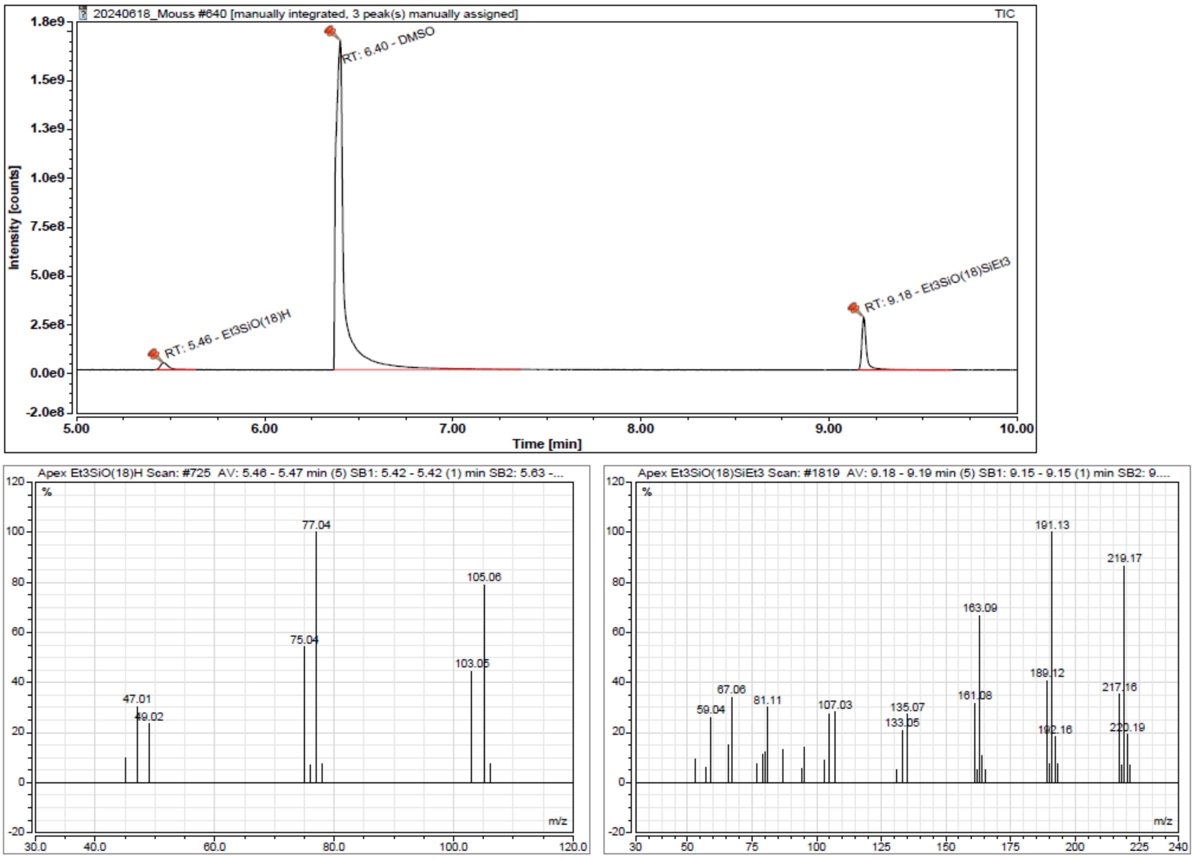

Figure S 20. GC-MS chromatogram of the reaction sample involving the hydrolysis of $HSiEt_3$ with $^{18}OH_2$ in DMSO and KO*t*Bu as catalyst. Note: About 30% of ^{16}O isotopically labels of both Si byproducts were detected, which is attributed to the contribution of moisture mainly from DMSO.

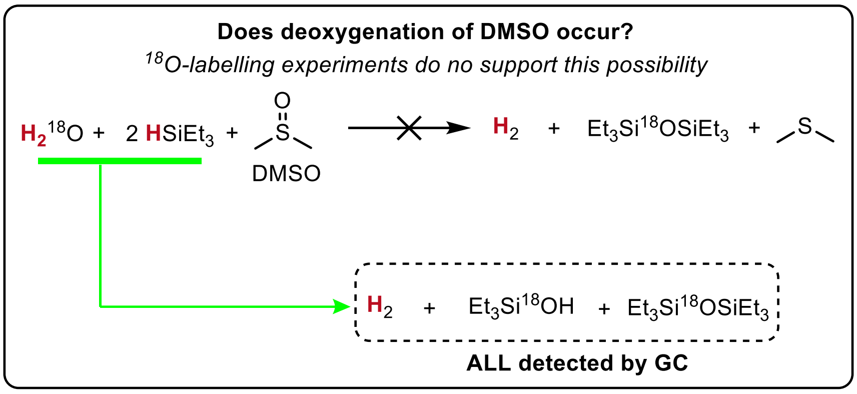

Figure S 21. Proposed (but experimentally ruled out) pathway for DMSO deoxygenation that might be involved in the hydrolysis of $HSiEt_3$ with $^{18}OH_2$ in DMSO and KO*t*Bu as catalyst.

7. Control experiments to exclude metal impurities

Adventitious metal trace: catalysis controls

Intentional addition of transition metal precursors.

Typical procedure for the preparation of the stock solution for the metal precursor. After drying the metal precursor under vacuum at 80°C for 24 h, a stock solution $(1.0 \times 10^{-2}\ M)$ in dried DMSO was prepared as follows: an appropriate amount of the sample was dissolved in 10 mL DMSO, and ultrasonication was applied if necessary.

Typical procedure for catalysis with transition metal addition. The general procedure for the silane hydrolysis was used, except that 10 ppm $(1.0 \times 10^{-6}\ M)$ of the given metal precursor was added to the reaction mixture using the stock solution prepared before.

As the data depicted in Figure S22 show, this study supports the claim that any contribution from a metal-mediated catalysis is very unlikely, if any, negligible. Surprisingly, in most of cases, addition of even ppm traces of metal additives seems to inhibit the catalysis (probably consuming part of KO*t*Bu). This study also demonstrates that the use of $Pd(OAc)_2$ alone (without KO*t*Bu) is not sufficient to catalyze the reaction.

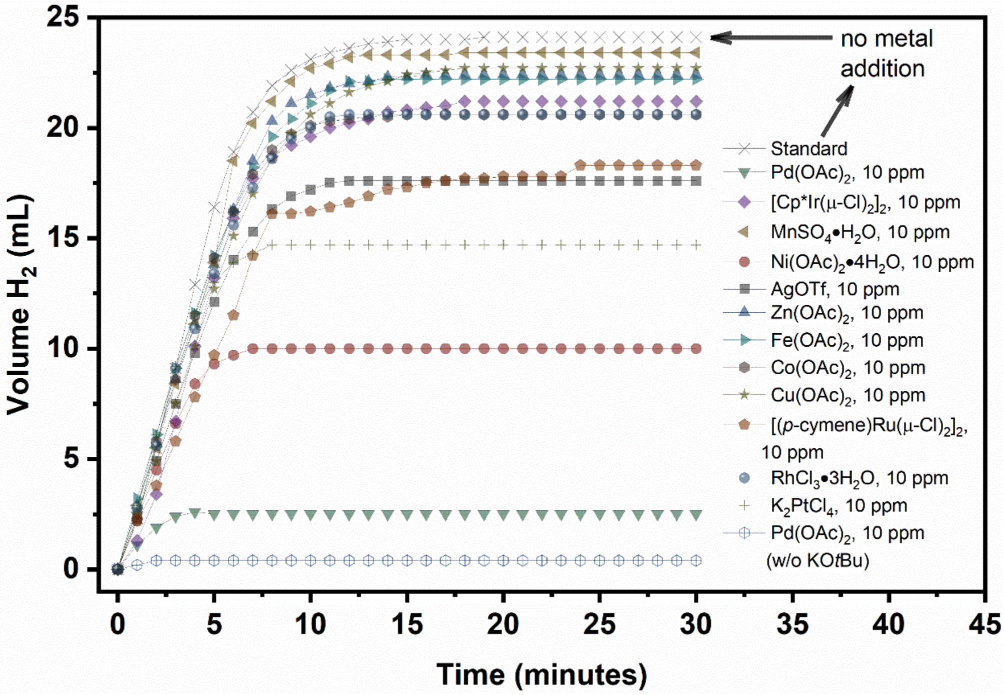

Figure S22. Intentional addition of transition metal precursors. Kinetic studies comparing of the silane hydrolysis reaction conducted under standard conditions (without metal addition) with the same reaction but conduced with intentional addition of 10 ppm of several transition metal precursors. Conditions: $HSiEt_3$ (129 mg, 1.11 mmol), DI-H_2O (0.02 mL, 1.11 mmol), KO*t*Bu (6.23 mg, 0.055 mmol), DMSO (0.5 mL), metal precursor (10 ppm), room temperature.

ICP-MS analysis of DMSO and KOtBu

ICPMS (inductively coupled plasma mass spectrometry) trace metal analysis was conducted on DMSO and KO*t*Bu because of their known solubilizing and coordinating abilities with regard to transition metal salts. The analysis revealed negligible metal content in both samples (*e.g.*, Ag, Pt, Pd, Rh, Ir ≤ 50 ppb; more data in Table S7).

Table S 7. ICP-MS analysis of the DMSO and KO*t*Bu samples that are used throughout this study (μg/Kg = ppb).

Sample	DMSO	KO^{t}Bu
Mn (μg/kg)	14	55
Fe (μg/kg)	161	595
Co (μg/kg)	19	<10
Ni (μg/kg)	51	319
Cu (μg/kg)	10.4	232
Zn (μg/kg)	84	586
Rh (μg/kg)	<0.1	<1.0
Pd (μg/kg)	1.4	28
Ag (μg/kg)	1.1	38
Ir (μg/kg)	3.8	29
Pd (μg/kg)	<5.0	<50
Pd (μg/kg)	14	55
**Procedure**	100 mg in 5 ml 20% HNO_3 + 0.2% HCl; no further dilution	dissolved 50 mg in 5 ml MQ; further dilution for analysis 1+4 in 20% HNO_3 + 0.2% HCl

Comparison of different batches of KOtBu. First, conducting the reaction using KO*t*Bu purchased from three different suppliers (Fluka, Aldrich and TCI) showed a decrease of ca. 10% in the catalytic activity for the Aldrich and TCI batches when compared to the Fluka batch that was usually employed throughout this study (Figure S23). The decreased catalytic activity is attributed to the older date of production and delivery for the Aldrich and TCI batches in comparison to the newer batch of Fluka. Second, the use of KO*t*Bu from another laboratory within our institute (Prof. Dr. O. Kappe) or the use of a fresh KO*t*Bu batch (ordered from Aldrich) also did not show a major deviation from standard H_2 generation kinetics (Figure S23). Similarly, no significant deviation could be seen from the standard H_2 generation kinetics in the case where freshly sublimed KO*t*Bu (freshly ordered from Aldrich) was used (Figure S23).

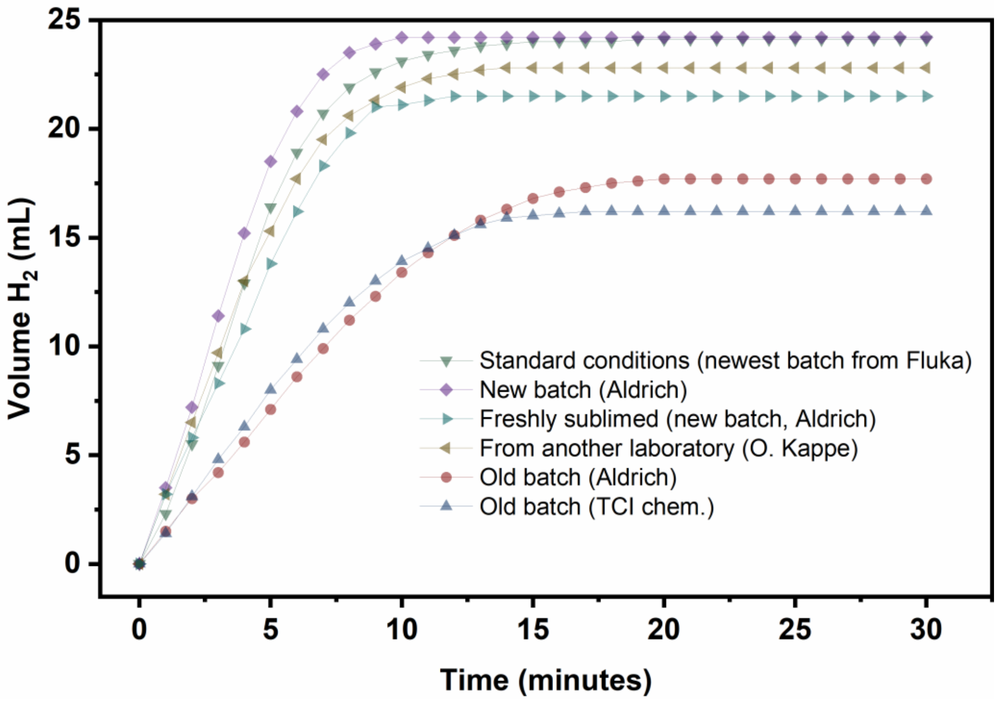

Figure S 23. Trace metal controls using different batches of KO*t*Bu: comparison of the volume of hydrogen (y-axis) as a function time (x-axis). Conditions: $HSiEt_3$ (129 mg, 1.11 mmol), DI-H_2O (0.02 mL, 1.11 mmol), KO*t*Bu (6.23 mg, 0.055 mmol), DMSO (0.5 mL). The maximum theoretical V_{H2} that can be released from 1.11 mmol H_2O is ≈ 24.5 mL.

Orthogonal metal poisoning experiments. Additional control experiments were performed using strong transition-metal chelators. Protic chelators (e.g., EDTA) were avoided as their acidic protons competitively react with both the silane and the base, confounding volumetric analysis. Instead, the strong aprotic ligands 2,2'-bipyridine (bipy) and triphenylphosphine (PPh_3) were utilized. As shown in Figure S24, the addition of 10 mol% of either ligand resulted in kinetic profiles that perfectly overlap with standard metal-free conditions. This lack of kinetic perturbation firmly rules out catalysis by adventitious trace metals.

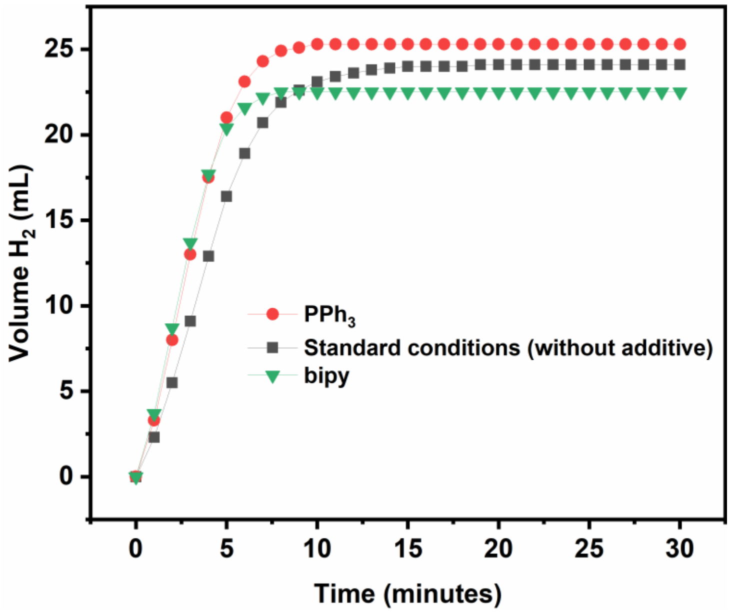

Figure S 24. The effect of different chelating ligand additives on the volume of hydrogen generated from the reaction of H_2O and $HSiEt_3$, in comparison with the standard conditions (red sphere dots). Conditions: Et_3SiH (1,11 mmol), H_2O (0.02 mL, 1.11 mmol), KOtBu (6.23 mg, 0.055 mmol), DMSO (0.5 mL). The maximum theoretical $V_{(H2)theo.}$ that can be released from reaction of 1.11 mmol H_2O with $HSiEt_3$

is ≈ 24.5 mL. For both additives, 10 mol% was added with respect to the limiting reagents (H_2O and $HSiEt_3$).

Using brand new glassware and freshly purified chemicals. When the reaction was conducted under the standard conditions using freshly distilled DMSO and $HSiEt_3$ as well as brand-new (unused) glassware and stir bar, the kinetics of hydrogen generation were very similar to those observed under standard conditions (Figure S25).

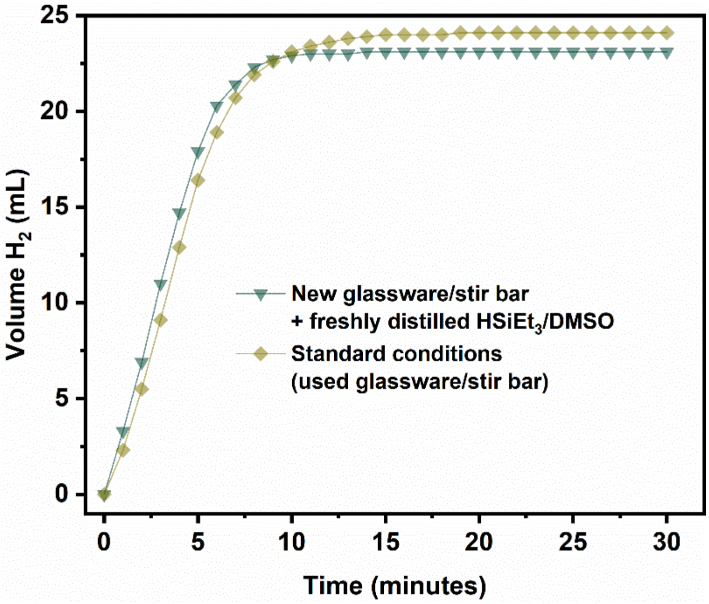

Figure S 25. Reaction material controls: comparison of the silane hydrolysis reaction usually conducted in a used glassware and stir bar with the same reaction carried out using an unused glassware and stir bar in combination with freshly distilled $HSiEt_3$ and DMSO. Conditions: $HSiEt_3$ (129 mg, 1.11 mmol), DI-H_2O (0.02 mL, 1.11 mmol), KO*t*Bu (6.23 mg, 0.055 mmol), DMSO (0.5 mL).

Using other types of reaction vessels. In order to exclude the potential effect from the borosilicate glassware used throughout this study, the reaction was conducted using either a brand-new plastic vial (Figure S26-A) or an unused stainless-steel reactor (Figure S26-B). No decrease in catalytic activity was observed.

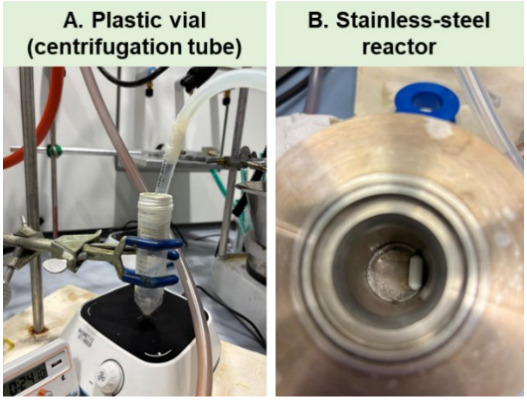

Figure S 26. Container controls: comparison of the silane hydrolysis reaction usually conducted in a borosilicate glassware (Schlenk tube) with the same reaction carried out either in a brand-new plastic vial (A) or a stainless-steel reactor (B). Conditions: $HSiEt_3$ (129 mg, 1.11 mmol), DI-H_2O (0.02 mL, 1.11 mmol), KO*t*Bu (6.23 mg, 0.055 mmol), DMSO (0.5 mL).

Inter-laboratory study control. The experiment was also repeated by another laboratory within our institute (Prof. Dr. W. Kroutil, experiment conducted by Federico Rossi), which revealed no major change in the overall kinetics (Figure S27). The person who carried out the experiment used chemicals and material from his own laboratory (DI-H_2O, $HSiEt_3$, DMSO,

KO*t*Bu, Schlenk tube, stir bar, spatula, pipettes, septum, syringe, etc). In conclusion, this result supports the claim that any contribution from a metal-mediated catalysis is very unlikely, if any, negligible. It also nicely demonstrates the reproducibility of our procedure.

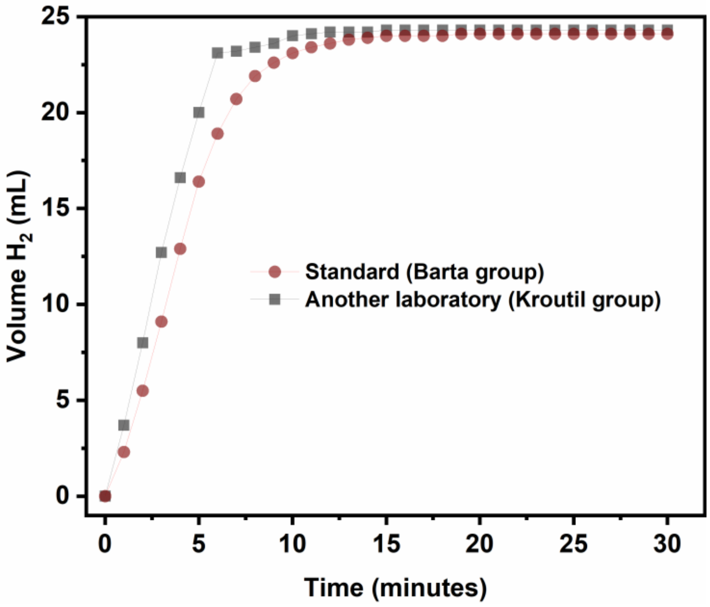

Figure S 27. Inter-laboratory study control: comparison of the silane hydrolysis reaction usually conducted in our laboratory (Barta group) with the same reaction carried out at an external laboratory (Kroutil group). Conditions: $HSiEt_3$ (129 mg, 1.11 mmol), DI-H_2O (0.02 mL, 1.11 mmol), KO*t*Bu (6.23 mg, 0.055 mmol), DMSO (0.5 mL).

8. Control experiments to exclude radical pathway

It was shown that the KO*t*Bu/DMSO system can behave as a radical initiator in several metal-free catalyzed transformations, with some examples showing that the presence of light could be a key component of the radical-initiation process.[37] In order to verify the possible role of radical species in our system, a series of control experiments and analyses were conducted (Figure S28). First, a DMSO:KO*t*Bu (211:1 molar ratio) mixture was analyzed by electron paramagnetic resonance spectroscopy (EPR), which showed no evidence of a radical species being generated under these conditions (Figure S28a–b). Second, radical trap experiments conducted in the presence of stoichiometric or catalytic amounts of TEMPO (2,2,6,6-tetramethylpiperidine-*N*-oxyl), a well-known radical scavenger,[38] showed that the hydrogen generation rate did not vary relative to the TEMPO-free standard conditions (Figure S28c–d). Likewise, when the reaction was conducted in the dark instead of the conventional laboratory day-light illumination no difference in hydrogen generation kinetics was noticed, thus ruling out any light-initiated radical species involved in the catalysis (Figure S28e). Irradiation of the reaction with Blue-LED (excluding day-light) also did not have any effect on the kinetics (Figure S28f).

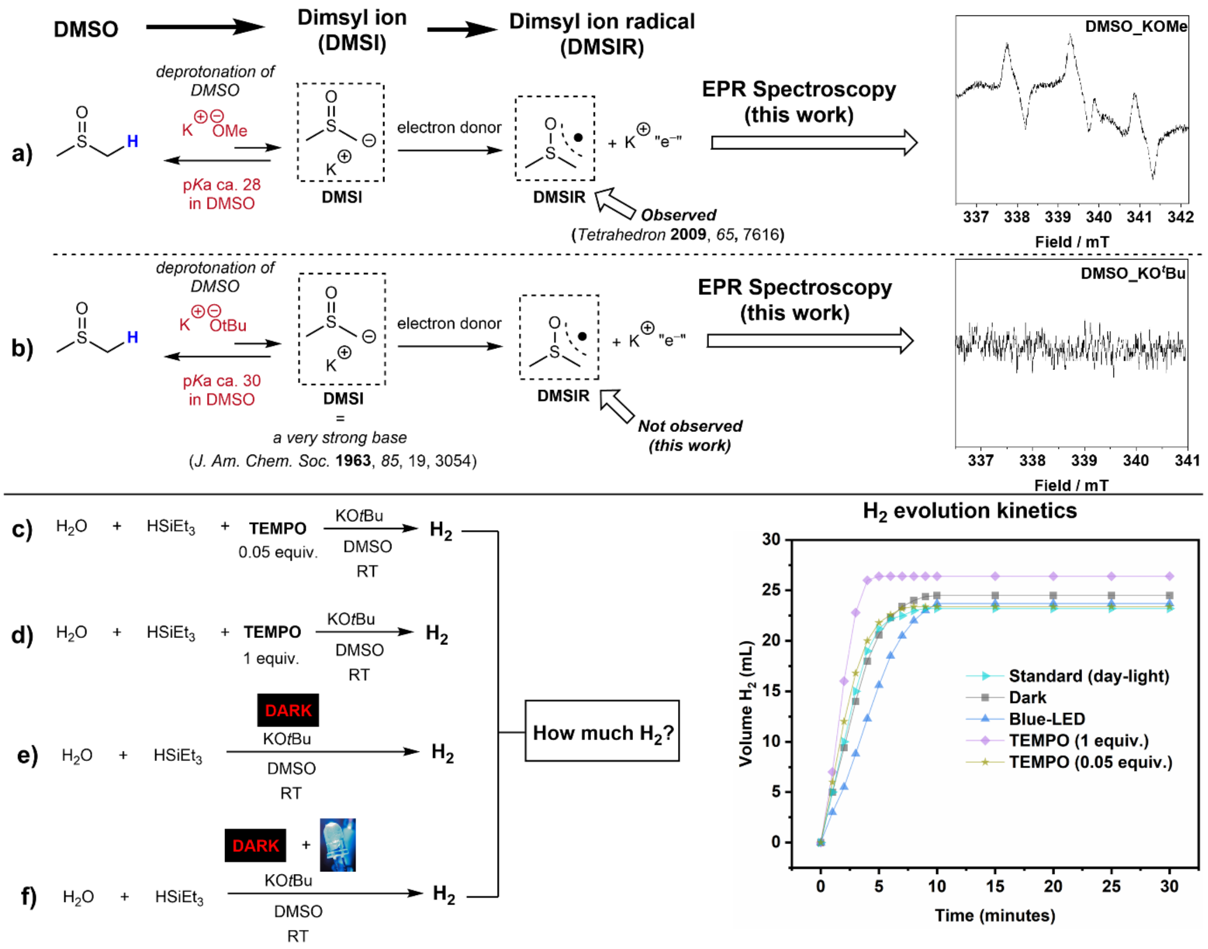

Figure S 28. EPR spectroscopy of the mixtures DMSO-KOMe (a) or DMSO-KO*t*Bu (b) and control experiments (c-f) with respect to the potential contribution from radical species in the silane hydrolysis.

9. Mechanistic aspects

a. Isolation of the new Si species K[HSi(OEt)$_3$(OtBu)]

In order to verify the mechanism of the reaction, the hypothesis of an ionic pentacoordinate hydrosilicate species was examined by the attempted isolation of the putative intermediates K[HSi(OEt)$_3$(OY)] by reaction of KOY with HSi(OEt)$_3$ (OY = OH, OMe, O*t*Bu). Attempts for the formation and isolation of the species K[HSi(OEt)$_3$(OH)] and K[HSi(OEt)$_3$(OMe)], using MeCN, DCM, toluene or THF, all led to decomposition upon solvent removal or recrystallization. However, K[HSi(OEt)$_3$(O*t*Bu)] was successfully isolated and characterized, using THF as the reaction medium (see details below).

Procedure for the preparation of K[HSi(OEt)$_3$(OtBu)]. A solution of HSi(OEt)$_3$ (200.0 mg, 1.22 mmol) in 1.6 mL THF was added dropwise to a slurry of KO*t*Bu (136.6 mg, 1.22 mmol) in 5 mL THF – some gas evolution was noticed. After stirring the reaction mixture at room temperature (RT) for 4 h, the resulting homogeneous yellowish solution was evaporated under vacuum until dryness. An oily viscous residue was obtained, which upon further drying under high vacuum turned into a yellowish residue. Then to the latter residue was added ca. 7 mL *n*-heptane then stored inside the freezer of the glovebox (–40 °C). After 24 h, no crystallization resulted from the latter sample. Therefore, the solvents were removed under vacuum yielding a yellowish residue, which was further dried until a 'sticky' white-yellowish solid residue was obtained. Then, the latter solid residue was washed three times with *n*-heptane (yellow heptane layer). The washed yellow residue was dried under vacuum for 2 h to give a less stickier white/pale-yellow residue (57.5 mg; max-theor.~ 337.3 mg; yield = 17%). Then, 22.3 mg were used for full NMR analysis in toluene-d_8 (yielding a clear homogeneous and colorless solution with soap-like foaming behavior), the data of which are presented below. However, due its high sensitivity against moisture, the identity of K[HSi(OEt)$_3$(O*t*Bu)] could only be ascertained by NMR spectroscopy. It was not possible to measure the sample by elemental analysis and mass spectrometry without chemically damaging it.

Discussion of the NMR data for K[HSi(OEt)$_3$(OtBu)]. The NMR data listed below as well as the corresponding spectral data (Figures S30–S36) all confirmed the identity of the adduct K[HSi(OEt)$_3$(O*t*Bu)]. However, these data also revealed that the isolated compound is a complex mixture consisting of one major isomer and one group of three minor isomers which were detected in ca. 2:1 ratio, respectively. The structure of the major isomer is tentatively assigned to a structure bearing the *tert*-butoxide ligand in axial position with respect to the hydride ligand, as supported by ^{1}H,^{1}H-NOESY NMR in Figure S35. Similar resonances were found for the remaining minor isomers to which we tentatively assign structures that correspond to three possible equatorial configurations of the adduct K[HSi(OEt)$_3$(O*t*Bu)] (Figure S29). More details can be found in related spectral images in the following section.

Nonetheless, the detection of the four isomers remains a puzzle. It is intriguing to observe such isomers at room temperature due to the fluxionality associated with the low energy barriers of the Berry pseudo-rotation, an exchange process usually studied at lower temperatures.[39] At this preliminary stage, it is still challenging to provide definitive evidence in support of our proposal, calling for additional in-depth experimental and computational studies. Nevertheless, as a preliminary explanation to this phenomenon, we postulate that each isomer is kinetically stabilized towards each other by the chelate-type coordination of the counter cation K^+ to three oxygen atoms provided by the ethoxy and *tert*-butoxide ligands (see possible structures in Figure S29). In support to this idea is the proposal by Corriu and coworkers of a similar structural arrangement of the counter cation K^+ around the ethoxy ligands of isolated pentacoordinate silicate species K[H$_2$Si(R)$_3$], explaining why the intramolecular $H_{axial}\ldots H_{equatorial}$ exchange in solvents such as benzene-d_6 or toluene-d_8 was prevented.[40]

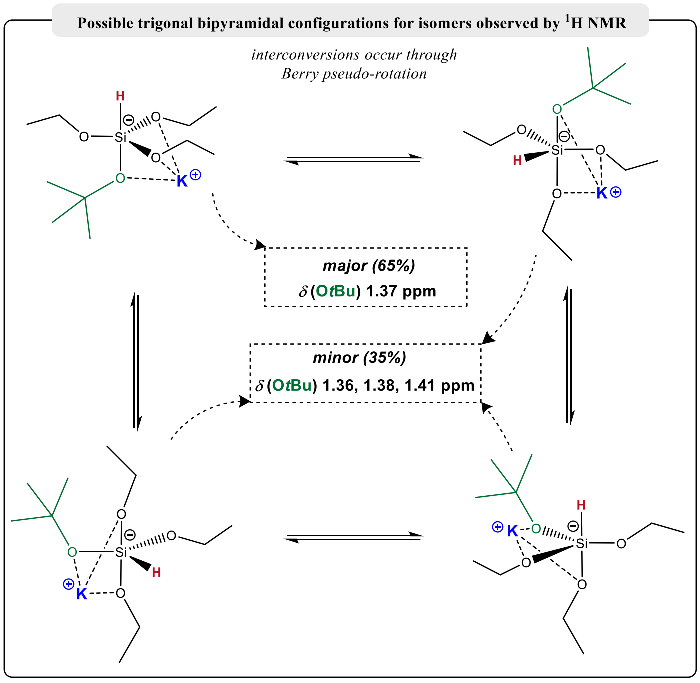

Figure S 29. Proposed structures for the putative configuration isomers of the adduct K[HSi(OEt)$_3$(O*t*Bu)].

The NMR data showed the characteristic ^{1}H and ^{29}Si resonance signals for the hydride and silicon at respectively δ 4.09 and –85.85 ppm. The latter resonances agree with those reported for the related pentacoordinate species KHSi(OEt)$_4$ (δ ^{1}H = +4.24 ppm in benzene-*d*$_6$; δ ^{29}Si = –86.2 ppm in THF-*d*$_8$)[40], and are shifted downfield in ^{1}H by ca. 0.34 ppm and shifted upfield in ^{29}Si by ca. 27 ppm relative to resonances of HSi(OEt)$_3$ (*cf.* Figures S37–S38 for details). Furthermore, the observed Si–H coupling constant, $^1J(^1\text{H}-^{29}\text{Si})$, for the adduct K[HSi(OEt)$_3$(O*t*Bu)] is ca. 25% smaller (210 Hz) than that of HSi(OEt)$_3$ (286 Hz). The decrease in $^1J(^1\text{H}-^{29}\text{Si})$ is a characteristic feature for a five-coordinate silicate compared to the corresponding four-coordinate neutral species, the origin of which is attributed to the decrease of the proportion of s character of the orbital in the Si–H bond.[40] This observation also suggests that the hydridic nature of the silicon-bound hydrogen should be more pronounced, which is in accord with the catalysis results of the present manuscript. Previous DFT calculations performed on the [HSi(Me)$_3$(O*t*Bu)]$^-$ anion as the model silicate showed that it readily releases a free hydride anion H$^-$ (ΔG ~ –11 kcal/mol).[41] The major isomer of the new adduct K[HSi(OEt)$_3$(O*t*Bu)] features ^{1}H and ^{13}C resonances for the Si-bound *t*BuO moiety that are both shifted upfield to respectively 1.37 ppm and 72.75/31.56 ppm, in comparison to those of free *t*BuO in pure KO*t*Bu (1.05 ppm and 68.08/31.35 ppm, respectively; see Figures S39–S40 for details). Similar observations were made for the other three minor isomers. These observations provide an additional support for the structural assignment of the isolated adduct K[HSi(OEt)$_3$(O*t*Bu)] and suggest that an electron density transfer mainly occurs from the C–O bond of the *t*BuO moiety to the Si–H bond, thus explaining the specific efficiency observed for KO*t*Bu when compared to most of other base catalysts.

NMR data for K[HSi(OEt)$_3$(OtBu)]

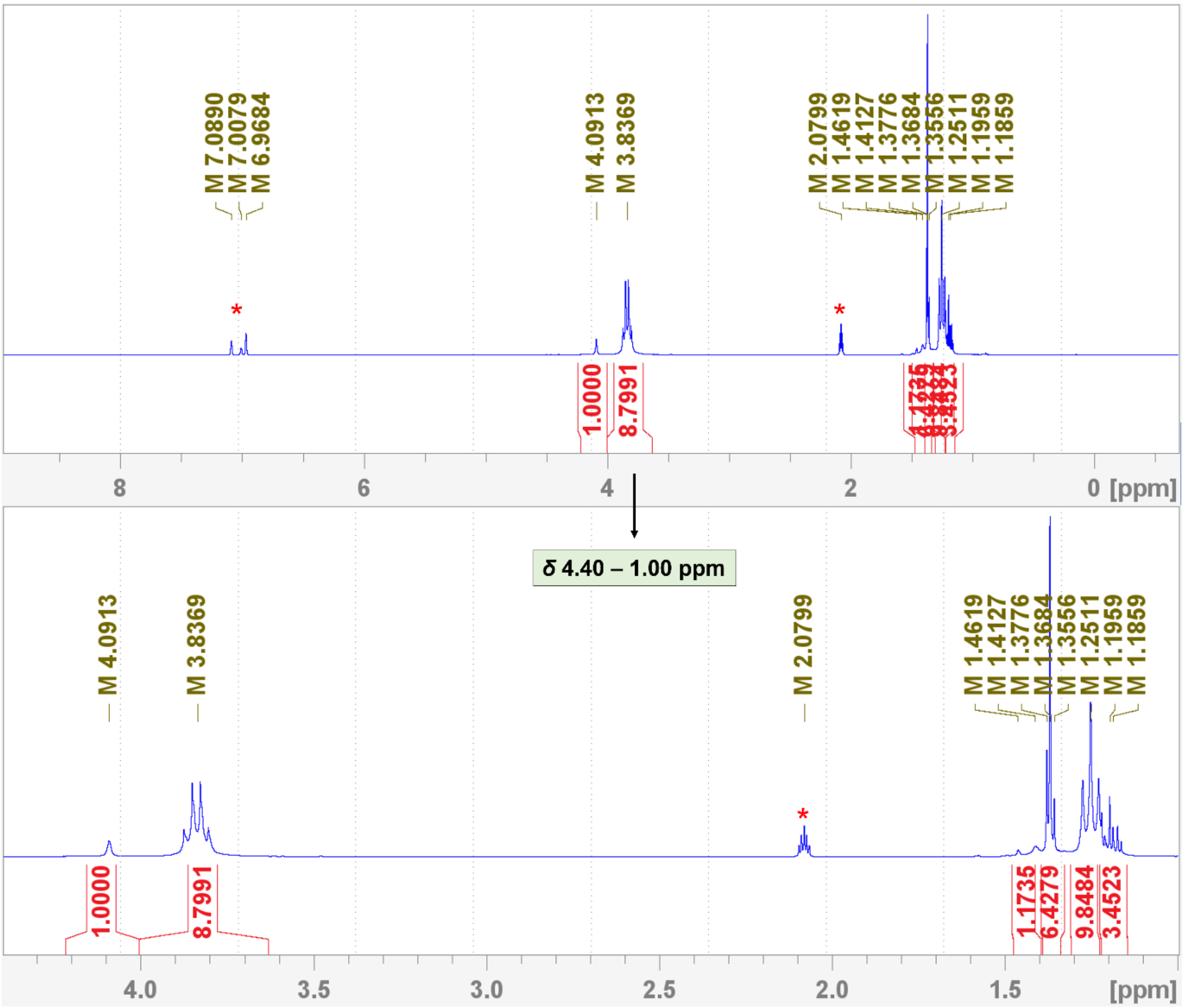

Figure S 30. ^{1}H NMR spectrum of the isolated K[HSi(OEt)$_3$(O*t*Bu)]. Solvent: Toluene-d_8(*). Spectrometer acquisition parameters: 300 MHz, 298 K.

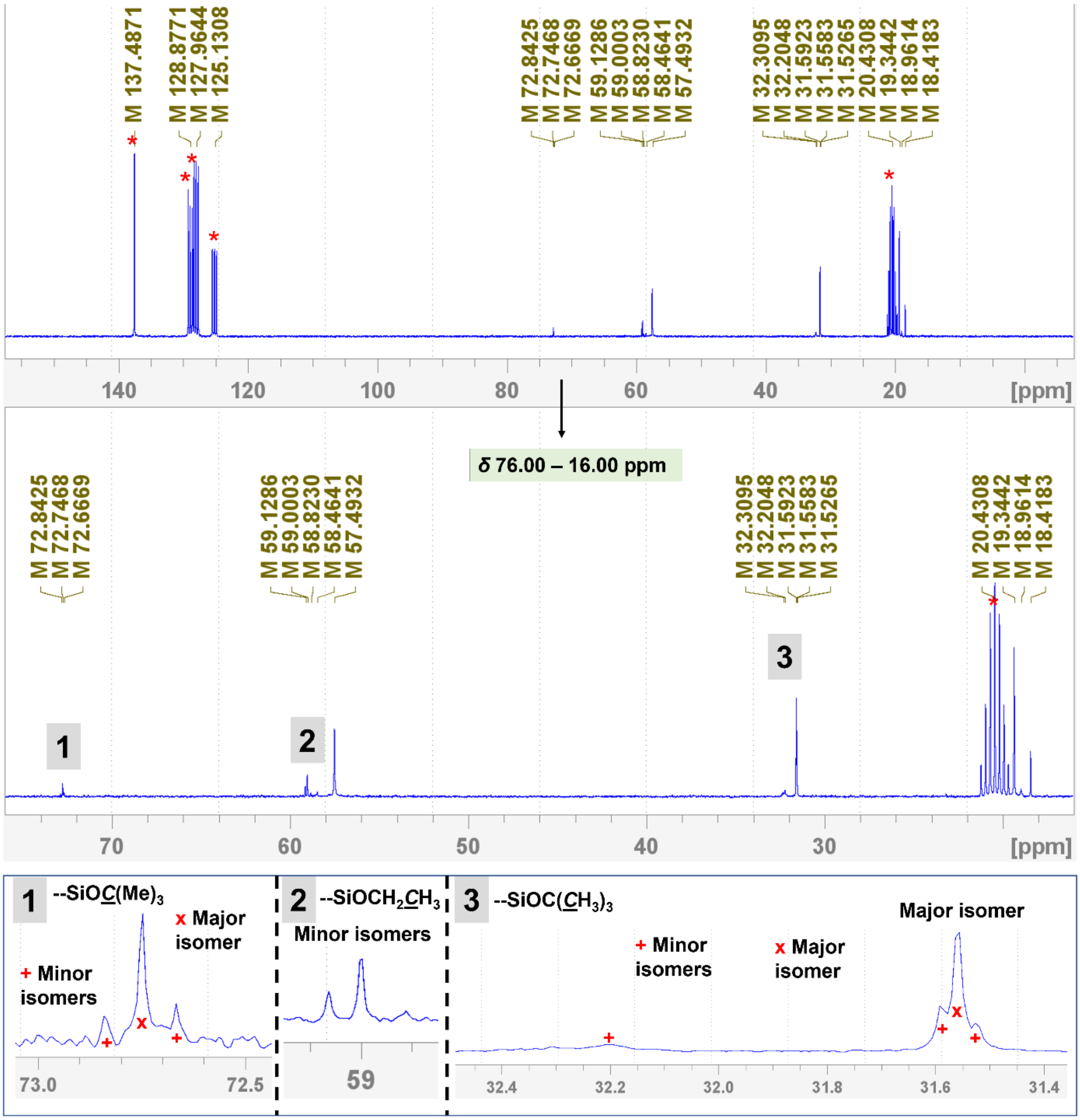

Figure S 31. $^{13}C\{^{1}H\}$ NMR spectrum of the isolated K[HSi(OEt)$_3$(O*t*Bu)]. Solvent: Toluene-*d*$_8$(*). Spectrometer acquisition parameters: Field at 300 MHz (^{13}C resonance at 75.5 MHz), 298 K.

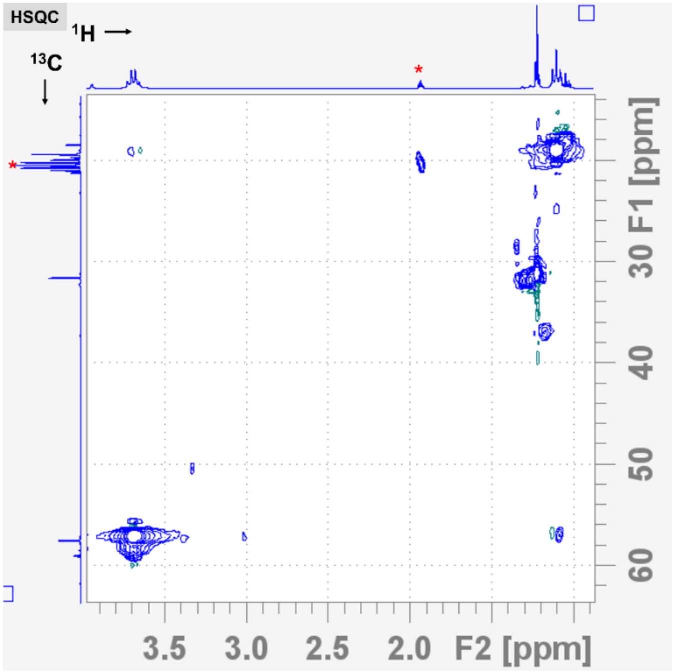

Figure S 32. ^{1}H-^{13}C HSQC NMR spectrum of the isolated K[HSi(OEt)$_3$(O*t*Bu)]. Solvent: Toluene-*d*$_8$ (*). Spectrometer acquisition parameters: Field at 300 MHz (^{13}C resonance at 75.5 MHz), 298 K.

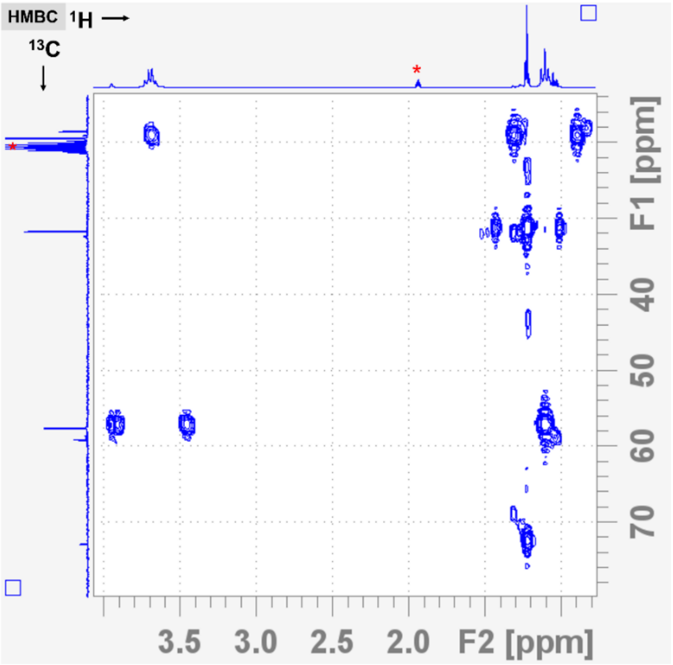

Figure S 33. ^{1}H-^{13}C HMBC NMR spectrum of the isolated K[HSi(OEt)$_3$(O*t*Bu)]. Solvent: Toluene-*d*$_8$ (*). Spectrometer acquisition parameters: Field at 300 MHz (^{13}C resonance at 75.5 MHz), 298 K.

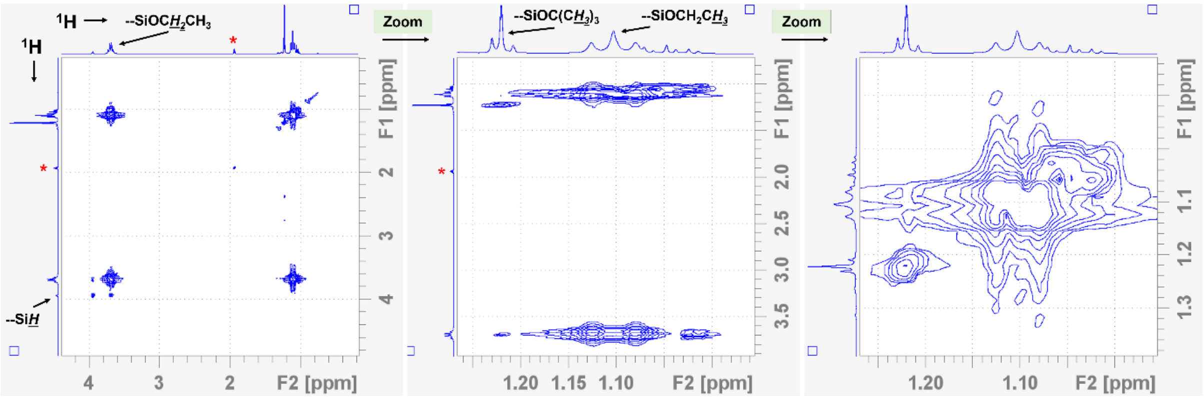

Figure S 34. ^{1}H-^{1}H COSY NMR spectrum of the isolated K[HSi(OEt)$_3$(O*t*Bu)]. Solvent: Toluene-*d*$_8$ (*). Spectrometer acquisition parameters: Field at 300 MHz, 298 K.

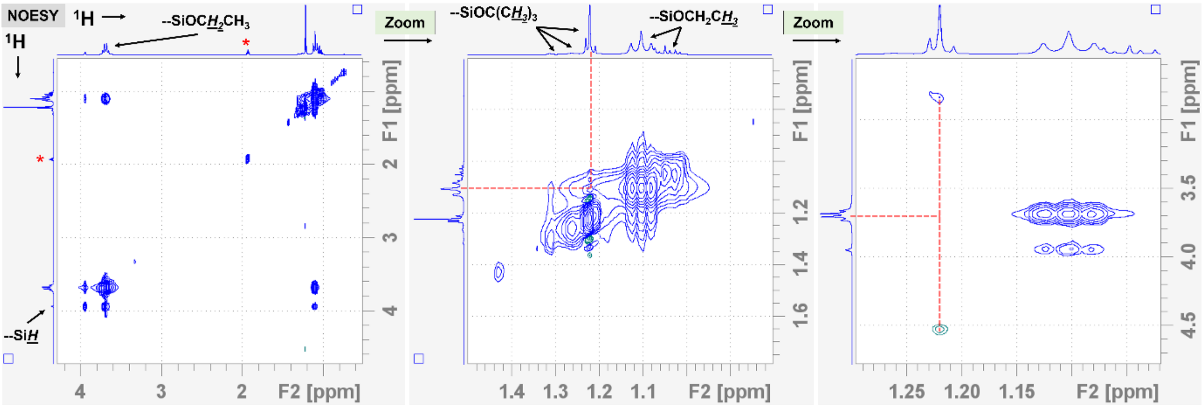

Figure S 35. ^{1}H-^{1}H NOESY NMR spectrum of the isolated K[HSi(OEt)$_3$(O*t*Bu)]. Solvent: Toluene-*d*$_8$ (*). Spectrometer acquisition parameters: Field at 300 MHz, 298 K.

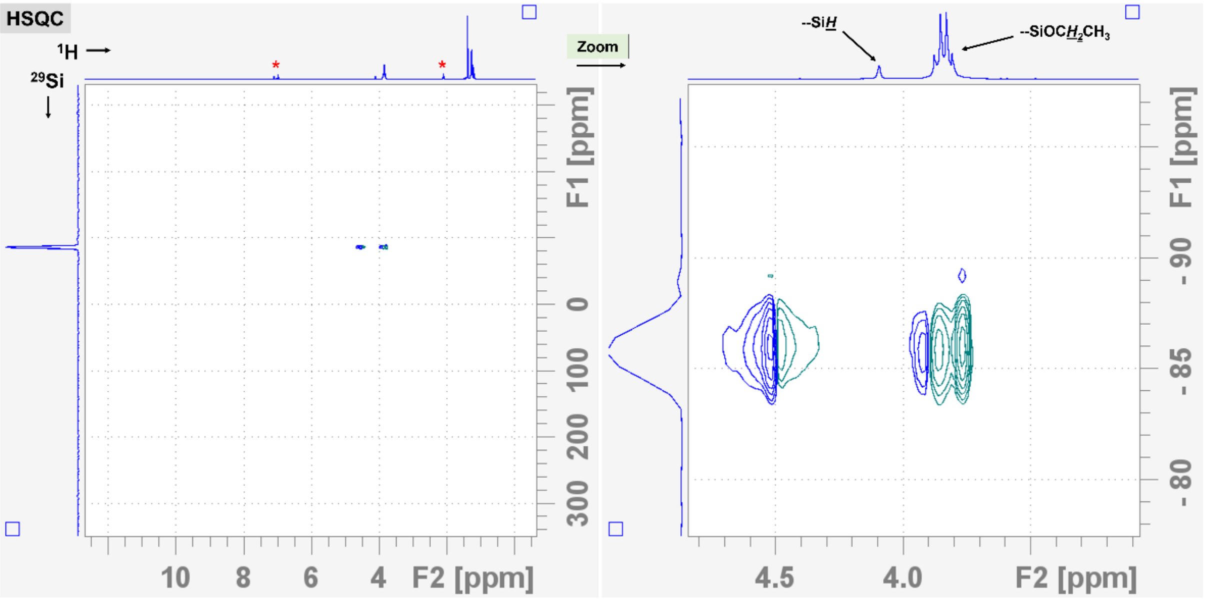

Figure S 36. ^{1}H-^{29}Si HSQC NMR spectrum of the isolated K[HSi(OEt)$_3$(O*t*Bu)]. Solvent: Toluene-*d*$_8$ (*). Spectrometer acquisition parameters: Field at 300 MHz (^{29}Si resonance at 59.6 MHz), 298 K.

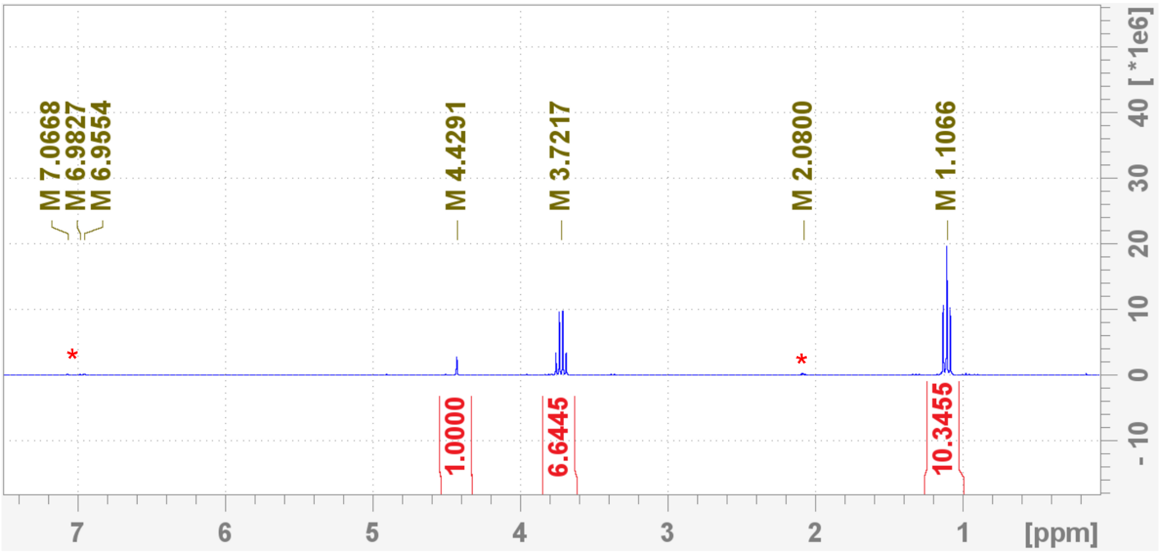

Figure S 37. ^{1}H NMR spectrum of $HSi(OEt)_3$. Solvent: Toluene-d_8(*). Spectrometer acquisition parameters: 300 MHz, 298 K.

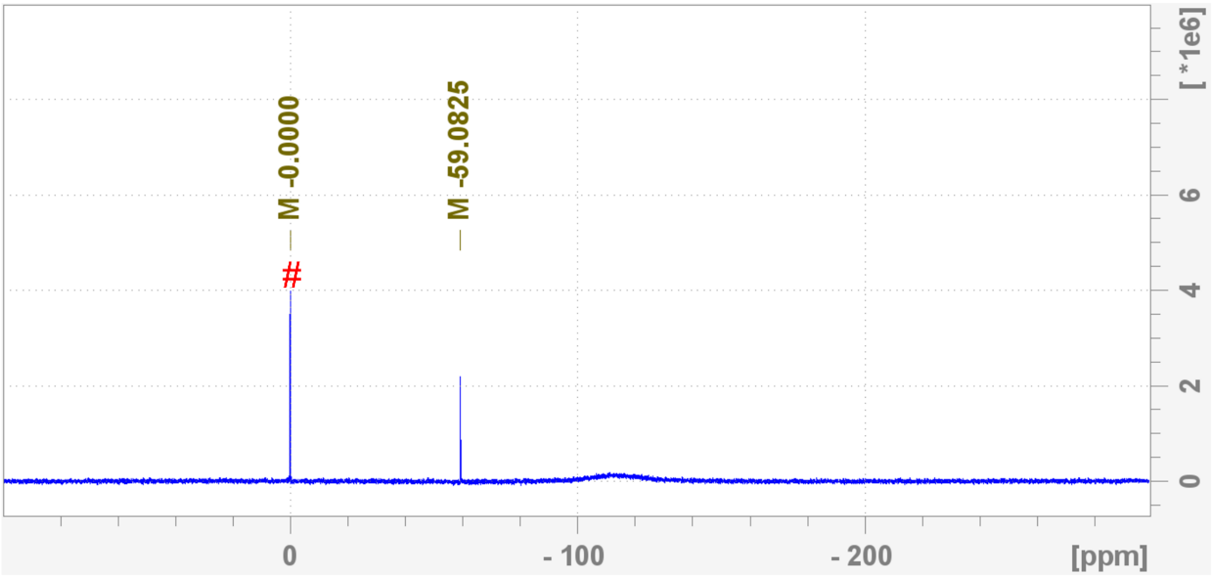

Figure S 38. ^{29}Si NMR spectrum of $HSi(OEt)_3$. Solvent: Toluene-d_8. Internal standard: $SiMe_4$(#). Spectrometer acquisition parameters: Field at 300 MHz (^{29}Si resonance at 59.6 MHz), 298 K.

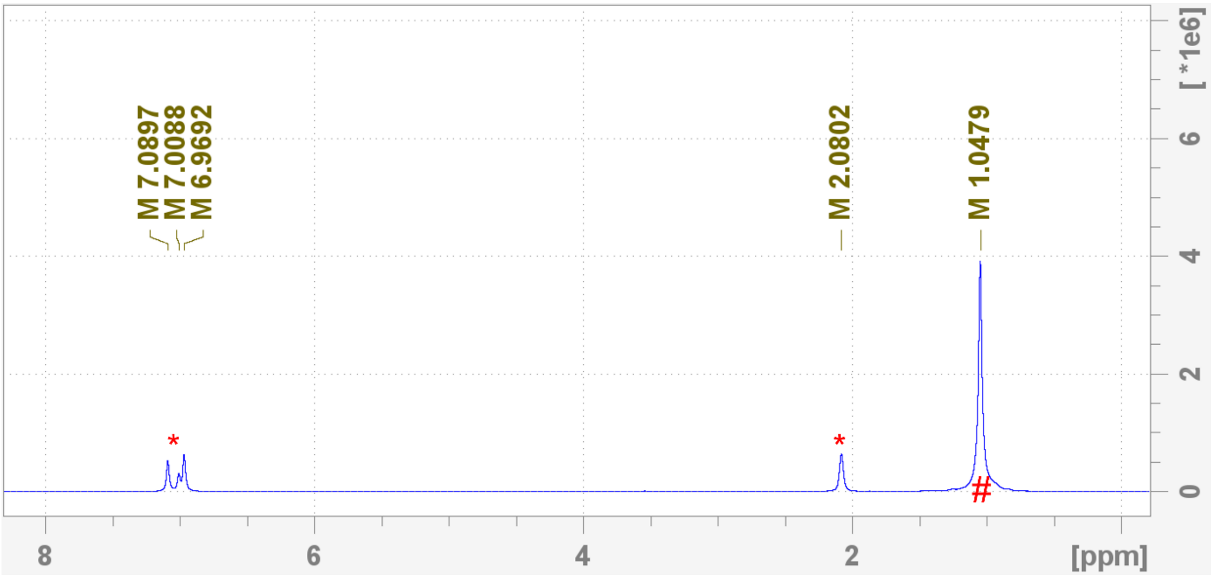

Figure S 39. ^{1}H NMR spectrum of KO*t*Bu. Solvent: Toluene-*d*$_8$(*). KO*t*Bu signal(#). Spectrometer acquisition parameters: Field at 300 MHz, 298 K.

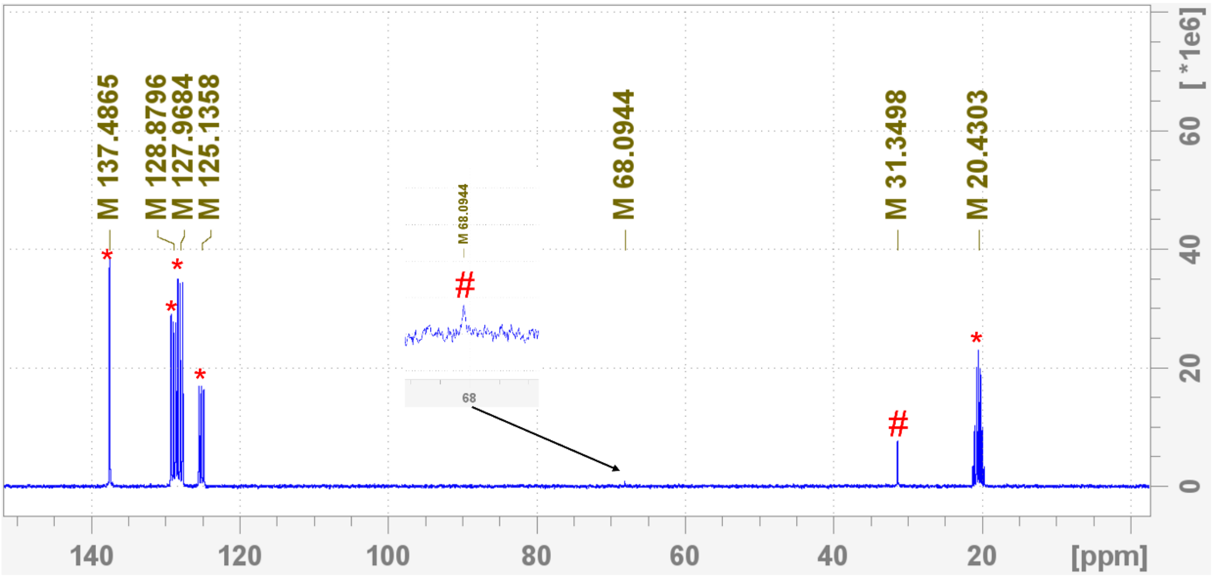

Figure S 40. $^{13}C\{^{1}H\}$ NMR spectrum of KO*t*Bu. Solvent: Toluene-*d*$_8$(*). KO*t*Bu signals(#). Spectrometer acquisition parameters: Field at 300 MHz (^{13}C resonance at 75.5 MHz), 298 K.

List of NMR data in ppm for K[HSi(OEt)$_3$(OtBu)] (toluene-d$_8$, 298 K, 300 MHz spectrometer)

***Note**. We list here the NMR data for the major axial isomer only (see structure in top left of Figure S29). Similar resonances were found for the minor isomers and more details can be found in related spectral images preceding with this section.*

^{1}H (300 MHz) 4.09 (s, 1H, $^{1}J(^{1}H\text{-}^{29}Si)$ = 210 Hz, Si-*H*), 3.84 (q, 6H, $^{3}J(^{1}H\text{-}^{1}H)$ = 7.05 Hz, Si-OC*H*$_2$CH$_3$), 1.37 (s, 9H, Si-OC(C*H*$_3$)$_3$), 1.25 (t, 9H, $^{3}J(^{1}H\text{-}^{1}H)$ = 6.97 Hz, Si-OCH$_2$C*H*$_3$); **$^{13}C\{^{1}H\}$ (75.5 MHz)** 72.75 (Si-O*C*(CH$_3$)$_3$), 57.49 (Si-O*C*H$_2$CH$_3$), 31.56 (Si-OC(*C*H$_3$)$_3$), 19.34 (Si-OCH$_2$*C*H$_3$). **$^{1}H\text{-}^{29}Si$ (300/59.6 MHz)** –85.85 ppm ($^{1}J(^{1}H\text{-}^{29}Si)$ = 210 Hz).

b. Stoichiometric reactivity of the isolated adduct K[HSi(OEt)₃(OtBu)]

In order to verify the plausible involvement of K[HSi(OEt)$_3$(O*t*Bu)] in the mechanism of the silane hydrolysis reaction, the stoichiometric reactivity of the isolated sample was examined by reaction with DI-H_2O, MeOH and DMSO. The resulting mixtures were analyzed by ^{1}H NMR spectroscopy and the results are displayed in Figures S41–S43. As the NMR data show, the reactions of K[HSi(OEt)$_3$(O*t*Bu)] with MeOH or DMSO gave mainly MeOSiEt$_3$ or the solvated species K[HSiR$_3$(O*t*Bu)]•DMSO$_n$, alongside H_2 and the liberated *tert*-butoxide (either as KO*t*Bu or protonated/methylate form). In the case of the reaction with DI-H_2O, the formation of mainly Si(OH)$_4$ alongside EtOH and H_2 were observed, i.e., the products of complete hydrolysis of the reactive intermediate fragment HOSi(OEt)$_3$. Therefore, these results support the hypothesis of K[HSi(OEt)$_3$(O*t*Bu)] as being a key intermediate along the mechanism of the silane hydrolysis reported in this study.

Procedure and reaction conditions for stoichiometric reactivity. Under nitrogen atmosphere, in a dry vial was first added ca. 10 mg of K[HSi(OEt)$_3$(O*t*Bu)] (3.4 μmol; 1 equiv.) in 0.6 mL toluene-*d*$_8$. Then DI-H_2O (one drop), MeOH (one drop) or 14 mg of DMSO (179.2 μmol; ca. 53 equiv.) was added. The content of the vial was mixed then quickly added to a sample NMR tube that was sealed before analysis.

NMR spectra for the stoichiometric reactions of K[HSi(OEt)₃(OtBu)]

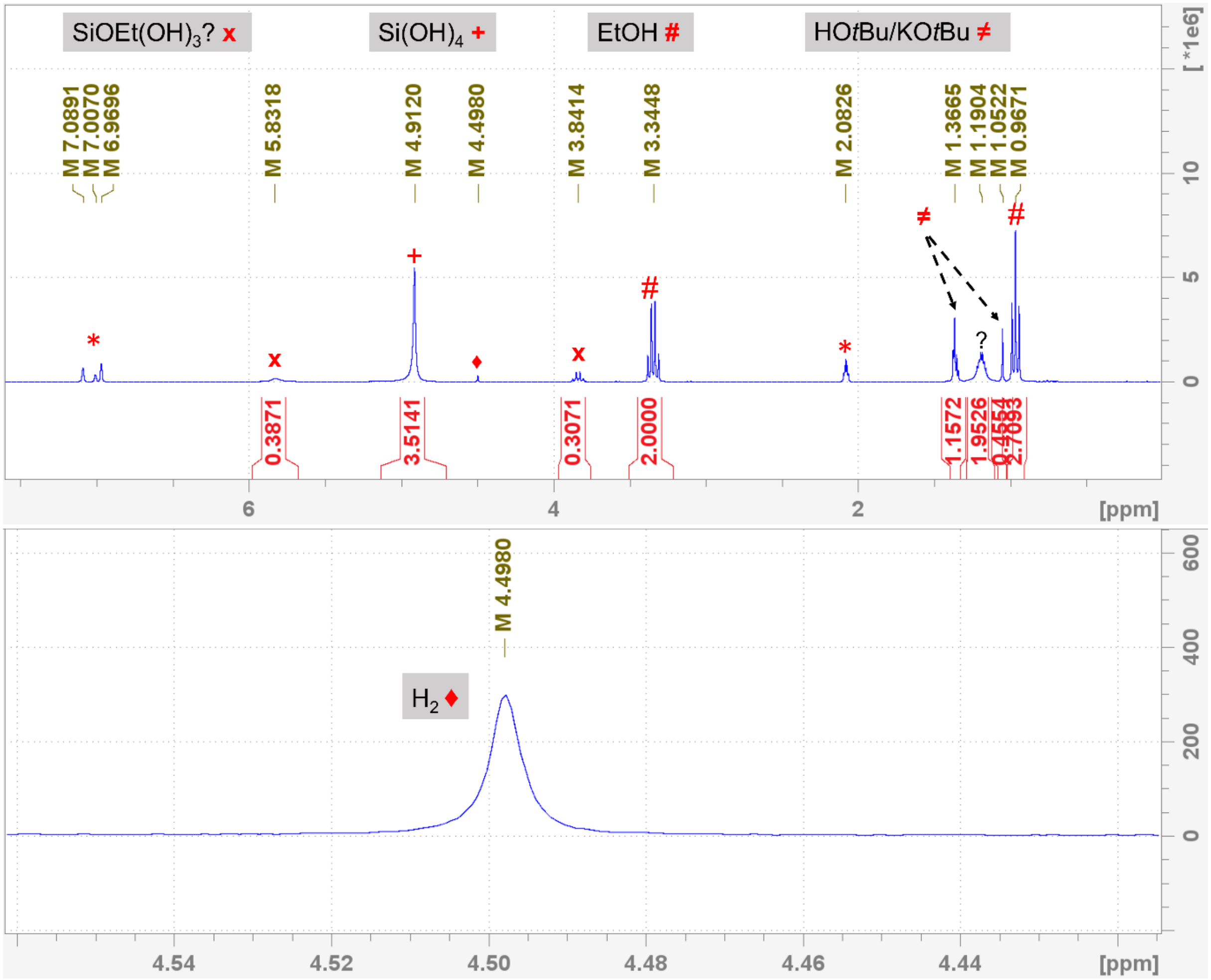

Figure S 41. ^{1}H NMR spectrum of the reaction mixture resulting from the stoichiometric reaction of K[HSi(OEt)$_3$(O*t*Bu)] with DI-H_2O. Solvent: Toluene-*d*$_8$(*). Spectrometer acquisition parameters: Field at 300 MHz, 298 K.

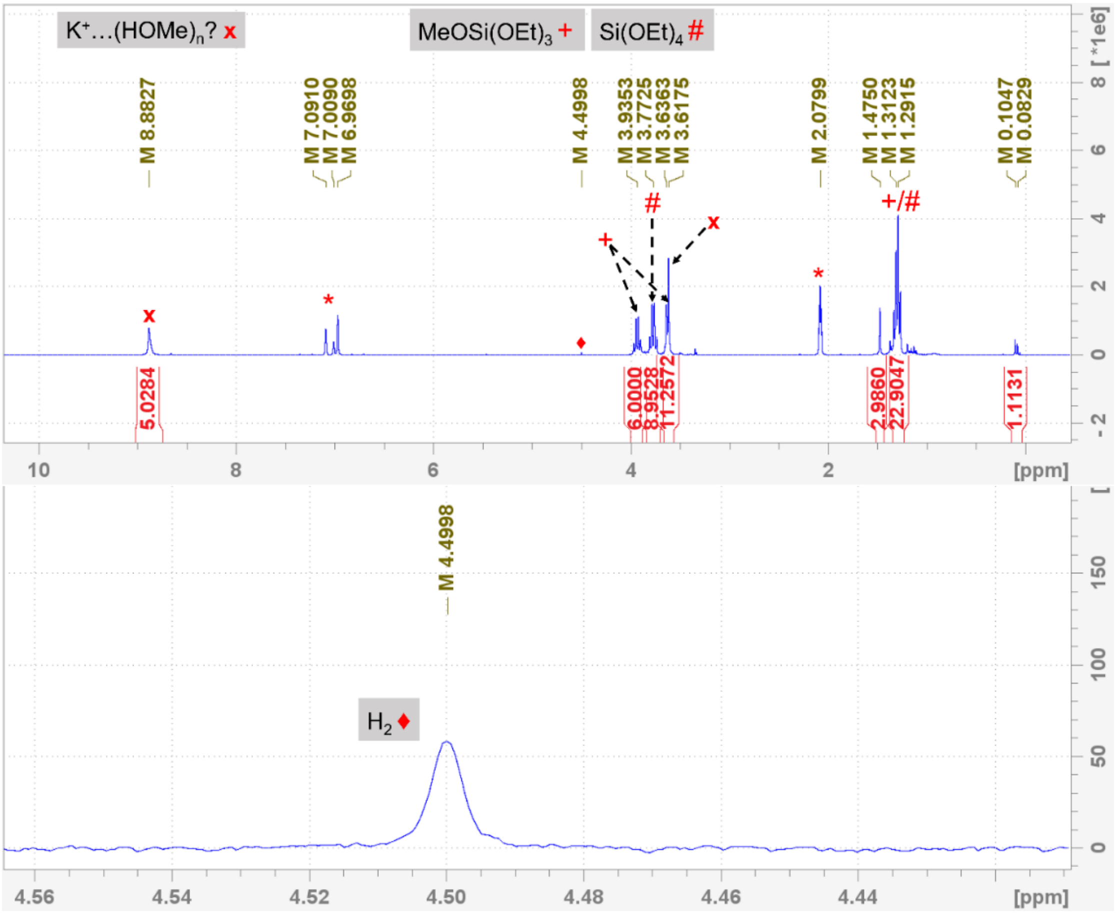

Figure S 42. ^{1}H NMR spectrum of the reaction mixture resulting from the stoichiometric reaction of K[HSi(OEt)3(O*t*Bu)] with MeOH. Solvent: Toluene-*d*8(*). Spectrometer acquisition parameters: Field at 300 MHz, 298 K.

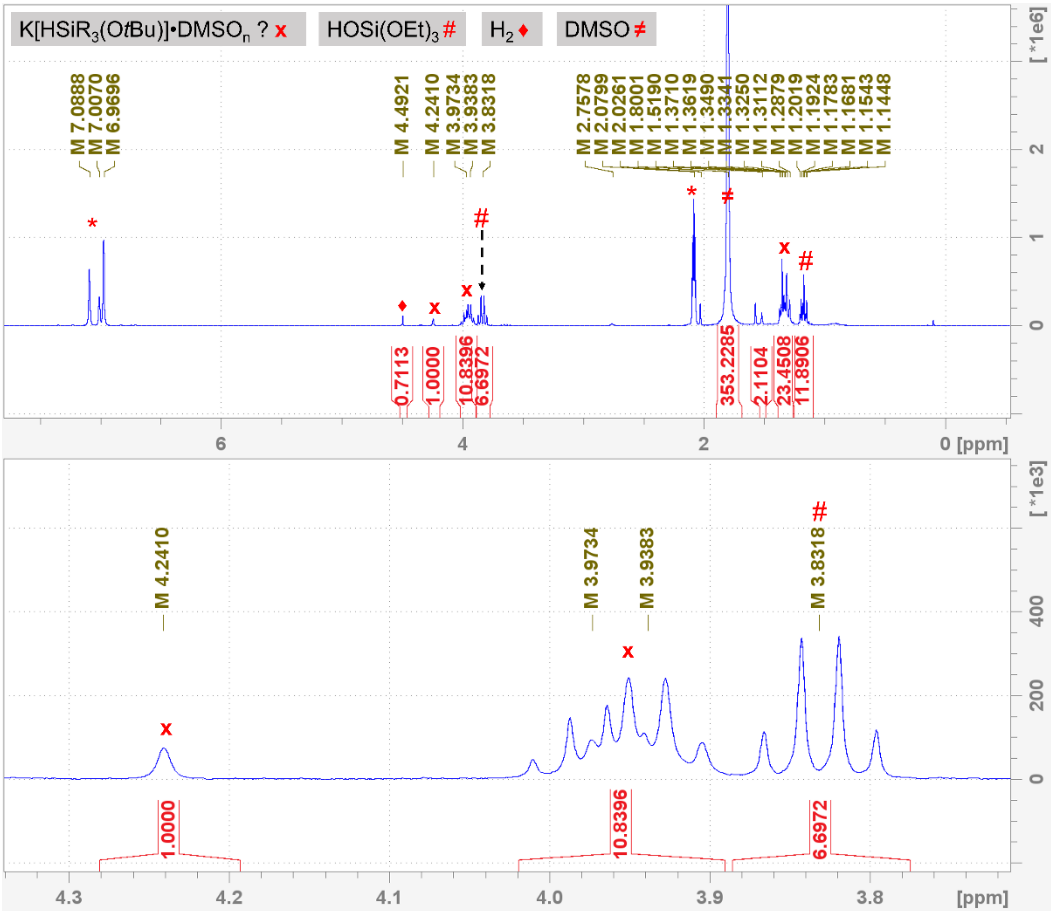

Figure S 43. ^{1}H NMR spectrum of the reaction mixture resulting from the stoichiometric reaction of K[HSi(OEt)$_3$(O*t*Bu)] with DMSO. Solvent: Toluene-*d*$_8$(*). Residual H_2 (♦) was formed because of the presence of moisture probably coming from DMSO; likewise for the putative silanol species HOSi(OEt)$_3$ (#). Spectrometer acquisition parameters: Field at 300 MHz, 298 K.

c. *Catalyst activity test of the isolated adduct K[HSi(OEt)$_3$(OtBu)]*

In order to verify the plausible involvement of K[HSi(OEt)$_3$(O*t*Bu)] in the mechanism of the silane hydrolysis reaction, the catalytic activity of the isolated sample was examined in the hydrolysis of HSi(OEt)$_3$ and compared to that of KO*t*Bu as catalyst. The results are displayed in Figure S44. As these data show, the activity of K[HSi(OEt)$_3$(O*t*Bu)] is comparable to that of KO*t*Bu. Therefore, these results support the hypothesis of K[HSi(OEt)$_3$(O*t*Bu)] as being a key intermediate along the mechanism of the silane hydrolysis reported in this study.

Procedure and reaction conditions for catalysis. In a dry Schlenk tube under nitrogen atmosphere was first added 20 µL of deionized (DI) water (1.11 mmol) and KO*t*Bu (6.2 mg; 5.5 µmol; 5 mol%) or K[HSi(OEt)$_3$(O*t*Bu)] (16.2 mg; 5.5 µmol; 5 mol%). Then dry 0.5 mL DMSO was added. The Schlenk tube was sealed under nitrogen atmosphere with a screw-thread cap equipped with a PTFE septum for injection. Finally, 182.4 mg of dry HSi(OEt)$_3$ (1.11 mmol) was quickly added with a syringe equipped with a long needle that penetrates smoothly through the PTFE septum until it reaches the top of the stirred reaction mixture. Just few seconds before injection of the silane, the side-arm of the Schlenk tube was open to the inverted burette for hydrogen collection and measurement (see Figure S3).

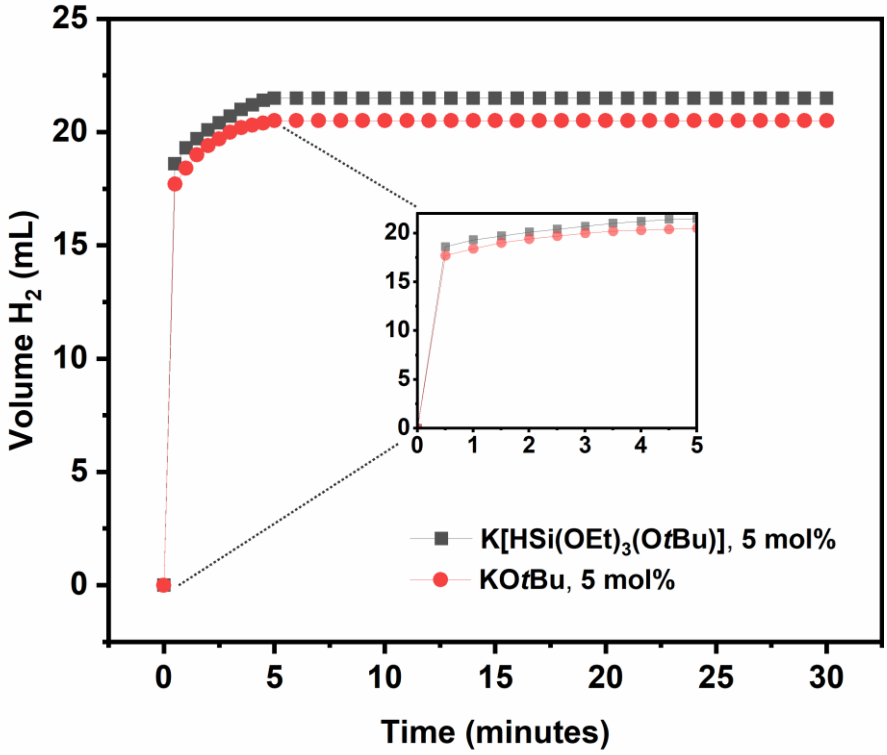

Figure S 44. Comparison of the kinetics of hydrogen generation from the hydrolysis of HSi(OEt)$_3$ reaction conducted under standard conditions (using KO*t*Bu as catalyst, gray square dots) with the same reaction but conducted with the freshly prepared K[HSi(OEt)$_3$(O*t*Bu)] as catalyst (red spherical dots). Conditions: HSi(OEt)$_3$ (182.4 mg, 1.11 mmol), DI-H_2O (0.02 mL, 1.11 mmol), catalyst (0.055 mmol; 5 mol%), DMSO (0.5 mL), room temperature.

d. Proposed formation pathways for the disiloxane $R_3SiOSiR_3$

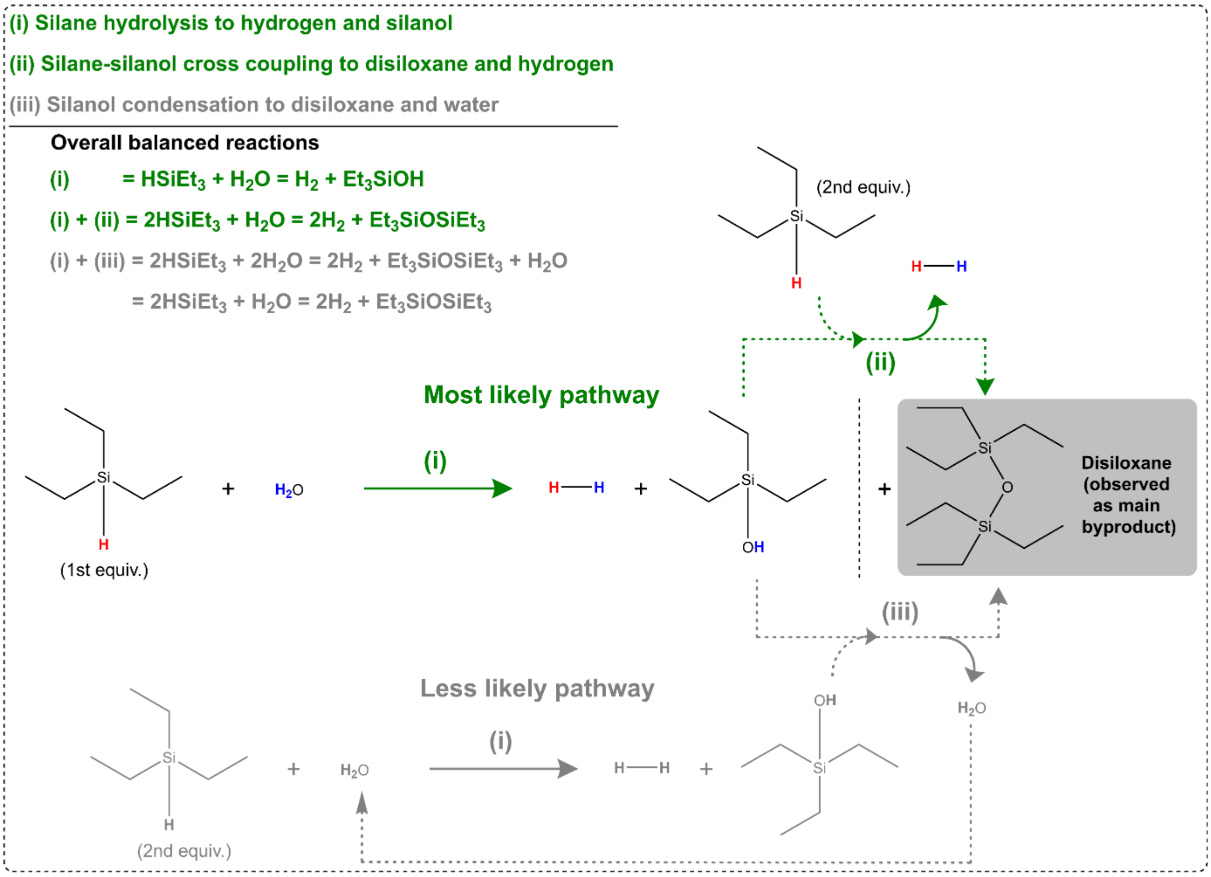

Figure S 45. Proposed hypothetical mechanism for the formation of the disiloxane from the hydrolysis of hydrosilane (triethylsilane is used as a model substrate). Although it is difficult to distinguish between the two pathways, we postulate that the pathway involving steps (i) and (ii) is the most likely to occur due to the pronounced hydride donor ability of the hydrosilane under the catalytic conditions used (catalytic KO*t*Bu and DMSO as co-solvent).

e. Computational studies

Detailed mechanistic investigations with Me_3SiH

As discussed in the main text, building up on the work of Voronova *et al*,[42] we initially assumed a mechanism as depicted in Figure S46, where the silane gets activated by the catalyst (OY^-). To remove the influence of the conformational space and, thus, to speed up the mechanistic investigations, most calculations were performed with Me_3SiH instead of Et_3SiH as the substrate (for the verification calculations on the $HSiEt_3$ system see section ii). The activation by the catalyst gives the overall system a negative charge and leads to the formation of an activated trigonal bipyramidal species $[HSiMe_3(OY)]^-$ (**int-A**), where the catalyst could be positioned either in the axial or equatorial position (Figure S47). Next, H_2O forms a dihydride interaction with the silicon-bound hydrogen of **int-A**, leading to the adduct of educts **int-B**. From here on H_2 evolution commences *via* transition state **TS1**, **int-C** and finally **TS2**, resulting in the formation of an agglomerate of the products H_2, OH^- and $SiMe_3(OY)$ (**prod**). The breakup of this agglomerate, i.e., escape of hydrogen gas and OH^-, then yields the free products and, accordingly, the state from which the overall free reaction energy must be determined.

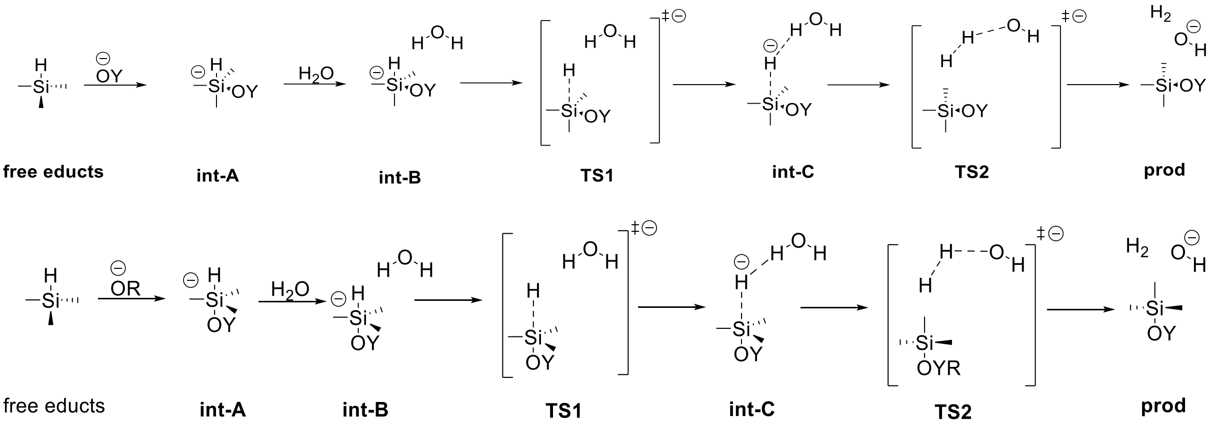

Figure S 46. Reaction pathways with catalyst OY$^-$ in equatorial (top) and axial (bottom) position, respectively.

The two catalysts considered (OtBu$^-$ and OH$^-$) and their respective binding modes (equatorial and axial) lead to four different investigated pathways (termed **OH$_{eq}$**, **OH$_{ax}$**, **O*t*Bu$_{eq}$** and **O*t*Bu$_{ax}$**).

Comparing the energy diagrams for the **OH$_{eq}$** and **OH$_{ax}$** pathways (Figure S47, left), it can clearly be seen that the rate determining energy barrier (**TS1**) is significantly lower for the axial configuration, and also the preactivated species **int-A** as well as the agglomerate of the educts **int-B** are energetically favored in the axial case.

The same observations are made for the **O*t*Bu$_{eq}$** and **O*t*Bu$_{ax}$** pathways (Figure S47, right). As for OH$^-$, also for O*t*Bu$^-$ as the catalyst the preactivated species **int-A** as well as the agglomerate of the educts **int-B** are energetically favored in the axial configuration. Regarding the rate determining energy barrier, while we were unable to obtain the structure of **TS1** for pathway **O*t*Bu$_{eq}$**, the educt adducts **int-A** of this pathway are already higher in energy than the highest transition states (**TS1**) of pathway **O*t*Bu$_{ax}$**. Hence, pathway **O*t*Bu$_{ax}$** demonstrates a lower overall barrier than its equatorial counterpart **O*t*Bu$_{eq}$**.

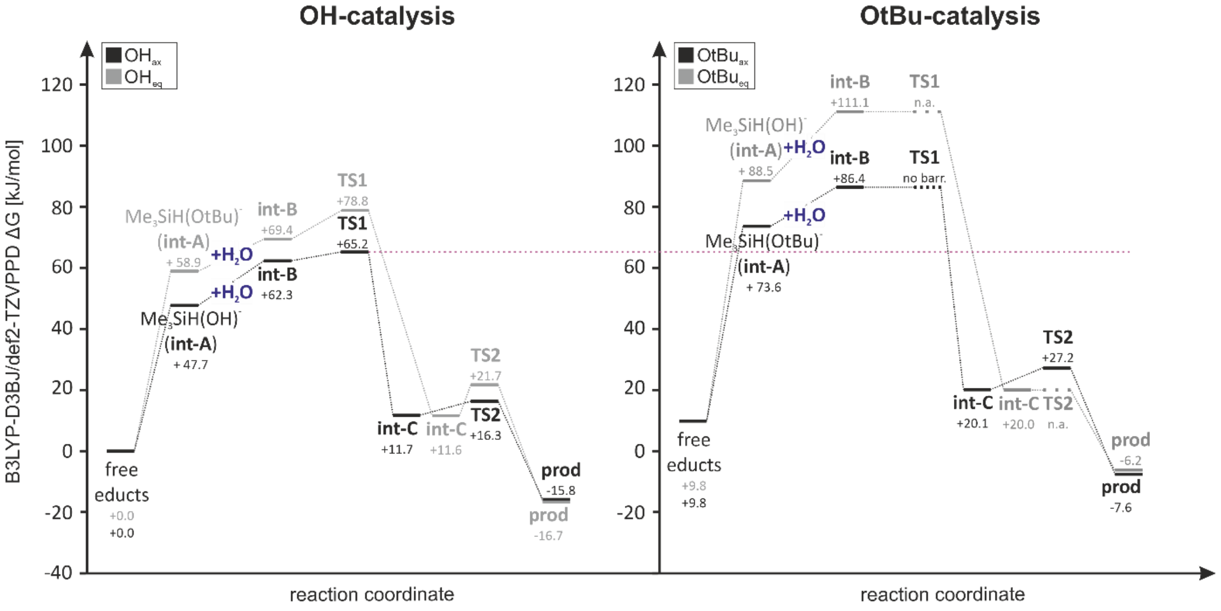

Figure S 47. Energy diagram of the investigated OH-catalyzed (left) and OtBu-catalyzed (right) pathways. ΔG values were calculated at the B3LYP-D3BJ/def2-TZVPPD@B3LYP-D3BJ/def2-SVPD level of theory employing the COSMO-GCM solvation model for DMSO. "Free educts" represents Me_3SiH, H_2O and OR'^- calculated separately. To be able to compare both OH^- and $OtBu^-$ on the same energetic scale, OH^- was chosen as the energetic reference and the reaction energy of HOtBu with OH^- was used to determine the energy of the free educts in the $OtBu^-$ case.

Comparing the two favorable pathways for each potential catalyst, i.e., **OH$_{ax}$** and **OtBu$_{ax}$**, OH-catalysis clearly exhibits a distinctly lower energy barrier than OtBu-catalysis (Figure S47), which can be chiefly attributed to the bulky tert-butyl group. Using the Eyring equation

$$k = A \exp\left(-\frac{\Delta G^{\ddagger}}{RT}\right)$$

the ratio of the rate constants $k_{\mathrm{OH,ax}}/k_{\mathrm{OtBu,ax}}$ of the axial pathways **OH$_{ax}$** and **OtBu$_{ax}$** was calculated to be 5179.5 at $T = 298.15$ K (assuming the preexponential factors A to be equal, R being the universal gas constant, and $\Delta G^{\ddagger}$ being the highest point along the reaction coordinate). This strongly indicates OH^- to be the overall better catalyst.

Moreover, as also stated in the main text, no matter the identity of the initial catalyst OY^-, the reaction always leads to the formation of OH^-. Since OH^- catalysis displays the much lower energetic barrier, it can be expected to take over unless prevented by other means. Together with nearly identical initial reaction rates of KOH and KOtBu (Figure 3 of the main text), this leads us to propose OH^- to be the main catalytic species. This is further supported by the observation of the background reaction without the presence of any added base catalyst (Figure 3 of the main text), as OH^- could also be formed by the autoprotolysis of water.

i. Geometric considerations regarding the preference for the axial pathway

Figure 48 to Figure S53 display the optimized geometries for each reaction step in the **OH$_{eq}$** pathway, calculated at the B3LYP-D3BJ/def2-SVPD level of theory and employing the COSMO-GCM solvent model for DMSO.

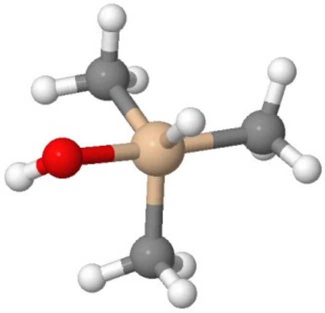

Figure S 48. int-A of pathway **OH$_{eq}$**, activated $Me_3SiH(OH)^-$ molecule.

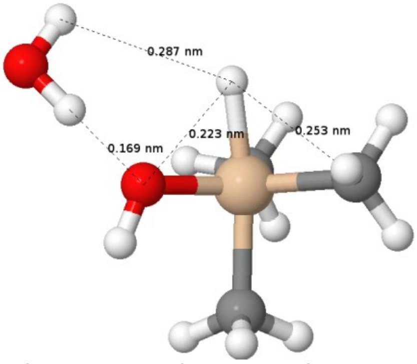

Figure S 49. int-B of pathway **OH$_{eq}$**, containing a hydrogen-bonded H_2O molecule. Note the angled Si-H bond and the missing H_2O hydride interaction (as opposed to the axial pathway, compare Figure S55).

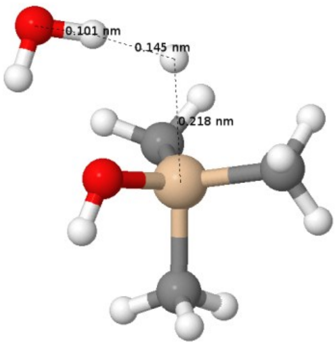

Figure S 50. TS1 of pathway **OH$_{eq}$**, breaking of the Si-H bond.

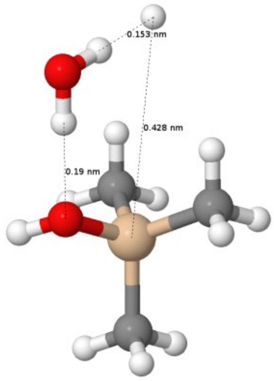

Figure S 51. int-C of pathway **OH$_{eq}$**, intermediate before H_2 evolution. Due to the equatorial arrangement, a hydrogen bond is formed between the water substrate and the bound catalyst moiety (as opposed to the axial pathway, compare Figure S57).

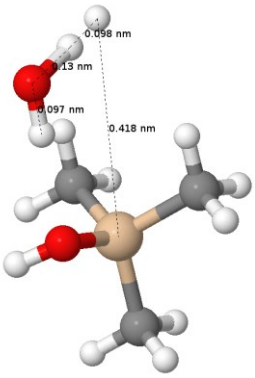

Figure S 52. TS2 of pathway **OH$_{eq}$**, elongation of the H-OH bond in the water molecule to form H_2 as indicated by the differing bond lengths in the H_2O (0.097 vs 0.130 nm).

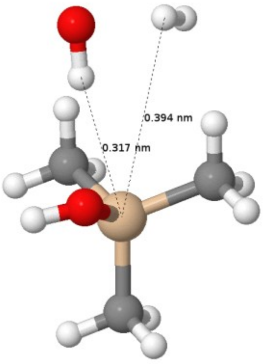

Figure S 53. prod of pathway **OH$_{eq}$**, completed H_2O deprotonation and geometric rearrangement of the individual products.

Figure S54 to Figure S58 display the optimized geometries for each reaction step in the **OH_{ax}** pathway, calculated at the B3LYP-D3BJ/def2-SVPD level of theory and employing the COSMO-GCM solvent model for DMSO.

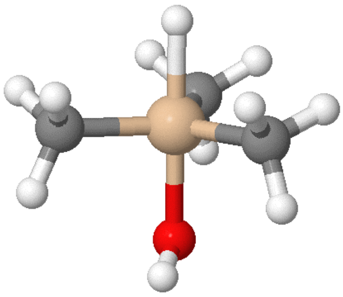

Figure S 54. int-A of pathway **OH_{ax}**, activated $Me_3SiH(OH)^-$ molecule.

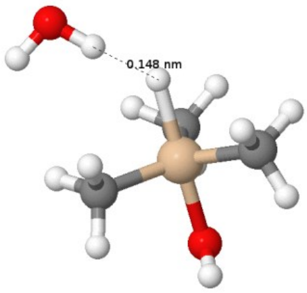

Figure S 55. int-B of pathway **OH_{ax}**, containing a hydrogen bonded H_2O molecule. Note the already formed H_2O hydride interaction (as opposed to the equatorial pathway, compare Figure S49).

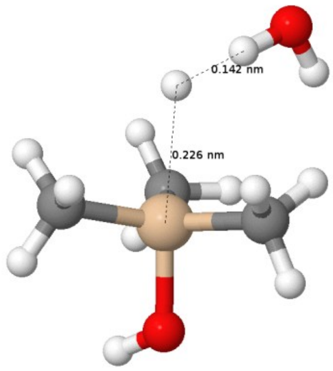

Figure S 56. TS1 of pathway **OH_{ax}**, breaking of the Si-H bond.

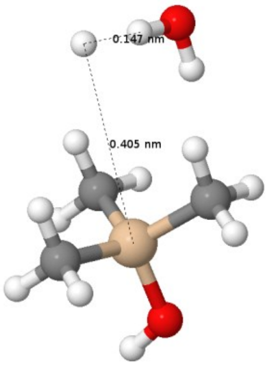

Figure S 57. int-C of pathway **OH_{ax}**, intermediate before H_2 evolution. No possibility of formation of a hydrogen bond between the water substrate and the bound catalyst moiety (as opposed to the equatorial pathway, compare to Figure S51).

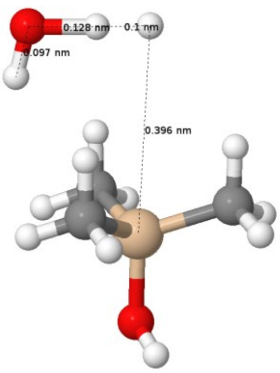

Figure S 58. TS2 of pathway **OH_{ax}**, elongation of the H-H_2O-bond to form H_2, as indicated by the differing bond lengths in the H_2O (0.097 vs 0.128 nm).

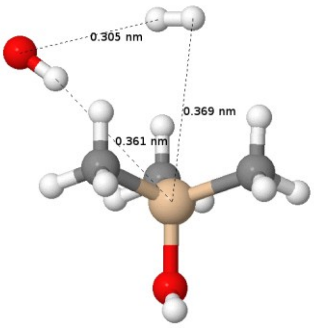

Figure S 59. prod of pathway **OH_{ax}**, completed H_2O deprotonation and geometric rearrangement of the individual products.

Since $OtBu^-$ and OH^- follow the same pattern for both equatorial and axial configuration (and since OH^- is indicated to be the significantly better catalyst), the corresponding geometries of the OtBu catalyzed pathways are omitted here.

Even though the equatorial positioning of the catalyst allows for hydrogen bonding between the Si-OH group and the water throughout the reaction (Figure S48 to Figure S53), the H_2O molecule is located further away from the hydride in the corresponding educt **int-B** of the **OH$_{eq}$** pathway (Figure S48), preventing the formation of the crucial beneficial dihydride interaction between H_2O and the silicon-bound hydrogen. Such a dihydride interaction is, however, present in **int-B** of the **OH$_{ax}$** pathway (Figure S55), whereas simultaneous hydrogen bonding to the Si-OH group is not possible for obvious geometric reasons. Moreover, the group located *trans* to the catalyst exhibits an elongated Si-CH_3 or Si-H bond, respectively, thereby weakening it (Figure S60 and Figure S61). This suggests both the elongation of the H-Si bond and the early formation of the H_2O-HSi hydrogen bond, already in **int-B** of the **OH$_{ax}$** pathway, to play a significant role in the overall energetical lowering of the axial reaction pathway. Also, the significantly lower relative energetic difference between **TS1** and **int-B** in the **OH$_{ax}$** pathway as compared to their equatorial congeners could be attributed to these effects.

An additional reason for the overall lower energies of the axial pathways can be found in the steric interactions of the methyl groups, which is already visible at the stage where the pentavalent **int-A** species are formed (Figure S60 and Figure S61). Positioning the catalyst axially allows for the maximum distance between the methyl groups, which are all equatorially arranged with an angle of approx. 120° (Figure S60). Contrarily, equatorial binding of the catalyst forces one of the methyl groups to adopt an axial position, thereby generating two approx. 90° angles between the methyl groups and creating unfavorable steric interactions (Figure S61).

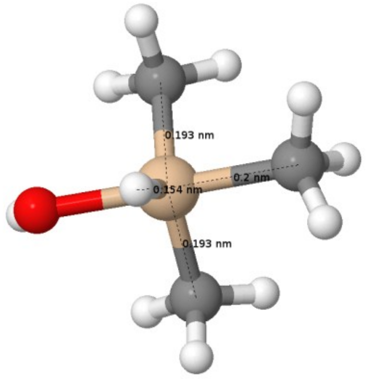

Figure S 60. int-A of pathway **OH$_{eq}$**, exhibiting bond elongation of the trans-positioned CH_3 group.

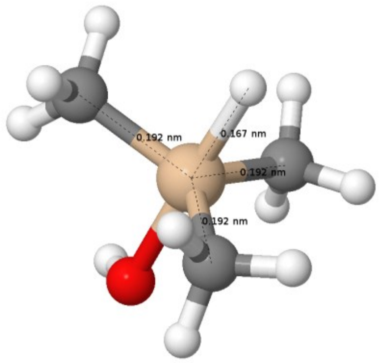

Figure S 61. int-A of pathway **OH$_{ax}$**, exhibiting bond elongation of the trans-positioned H atom.

ii. Mechanistic investigations with Et3SiH

To verify our computational results obtained for the Me_3SiH model system, we subsequently verified our mechanistic rationale by recalculating the initial steps (**int-A** and **int-B**) with the experimentally tested Et_3SiH (S62). Expectedly, the energies of the ethyl species are generally slightly higher than their methyl congeners due to the higher steric demand of the ethyl side chains. Overall, the energetic trend is not impacted by the nature of the silane side chains and we therefore consider any conclusions drawn from the Me_3SiH model system to be valid also for the Et_3SiH (and related) systems. Hence, we did not pursue our calculations any further from this point, i.e., **TS1** and subsequent steps were omitted for Et_3SiH.

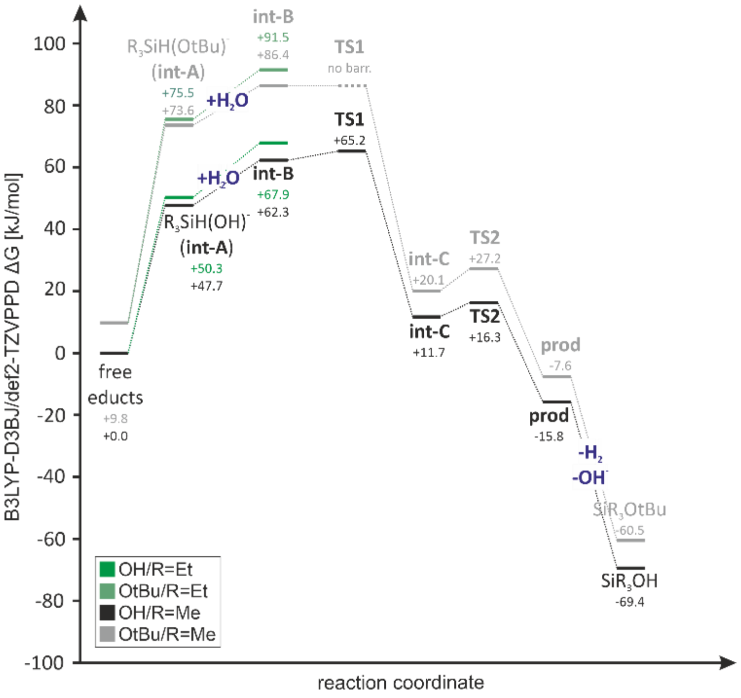

Figure S 62. Energy diagram of the proposed mechanism of the reaction of Me_3SiH or Et_3SiH with H_2O, assuming OH^- and $OtBu^-$ to be the catalyst, respectively. ΔG values were calculated at the B3LYP-D3BJ/def2-TZVPPD@B3LYP-D3BJ/def2-SVPD level of theory employing the COSMO-GCM solvation model for DMSO. “Free educts” represents Me_3SiH, H_2O and OR'^- calculated separately. To be able to compare both OH^- and $OtBu^-$ on the same energetic scale, OH^- was chosen as the energetic reference and the reaction energy of HOtBu with OH^- was used to determine the energy of the free educts in the $OtBu^-$ case.

iii. *Rationale for the synthesis of K[(EtO)$_3$SiH(OtBu)] – Gibbs Free Energies of the Pentavalent Species of Type **int-A***

Given the experimental isolation of K[(EtO)$_3$SiH(O*t*Bu)], the high energies of the calculated **int-A** species $Me_3SiH(OH)^-$, $Me_3SiH(OtBu)^-$, $Et_3SiH(OH)^-$ and $Et_3SiH(OtBu)^-$ (see Figure S62 and Figure S63) initially appeared slightly puzzling since such high energy intermediates would *a priori* not be expected to be isolable. However, we suspected that the nature of the side chains of the silane, i.e., alkyl vs alkoxy, could play an important role regarding the possibility to synthesize K[(EtO)$_3$SiH(O*t*Bu)] in the lab.

To test our mechanistic rationale for consistency with the experimentally observed behavior, we therefore opted to model the corresponding alkoxy congeners $(MeO)_3SiH(OH)^-$, $(MeO)_3SiH(OtBu)^-$, $(EtO)_3SiH(OH)^-$ and $(EtO)_3SiH(OtBu)^-$. Notably, exchanging the alkyl for alkoxy side chains has a profound stabilizing effect on the pentavalent species **int-A** and also the corresponding H_2O adducts **int-B** (Figure S63). As opposed to their alkyl congeners, the formation of the species of type **int-A** is even rendered exergonic compared to the free educts for all silanes bearing alkoxy side chains but (EtO)$_3$SiH(OH), thus explaining the successful synthesis of K[(EtO)$_3$SiH(O*t*Bu)].

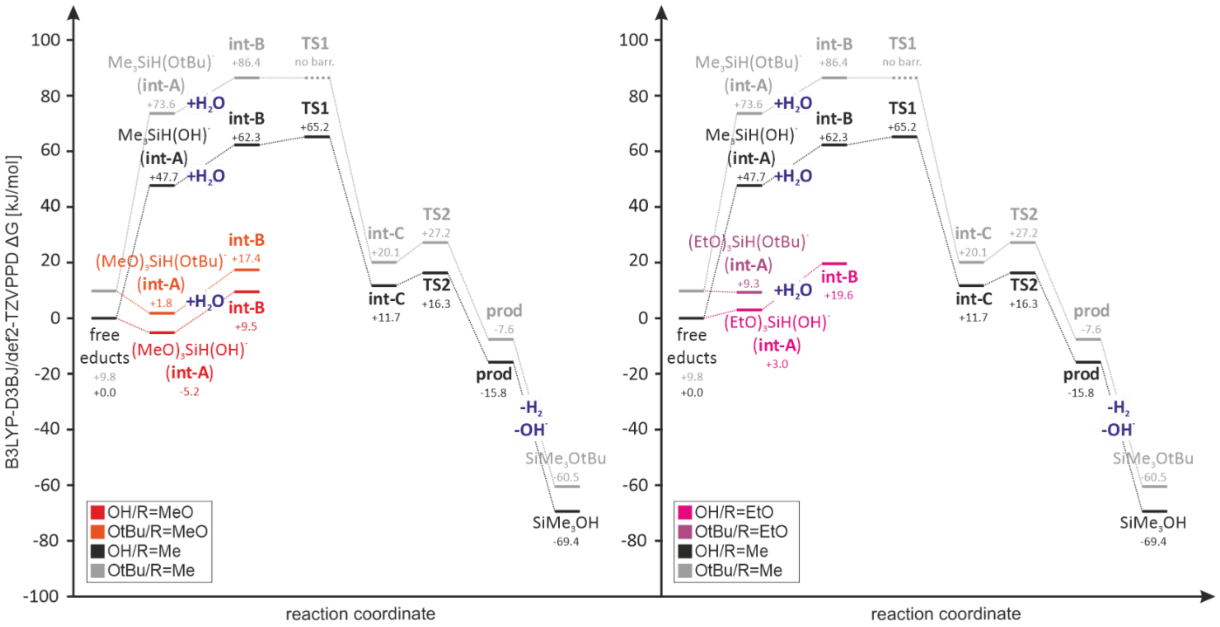

Figure S 63. Energy diagram of the proposed mechanism of the reaction of Me_3SiH/MeO_3SiH (left) and Et_3SiH/EtO_3SiH (right) with H_2O, assuming OH^- and $OtBu^-$ to be the catalyst, respectively. ΔG values were calculated at the B3LYP-D3BJ/def2-TZVPPD@B3LYP-D3BJ/def2-SVPD level of theory employing the COSMO-GCM solvation model for DMSO. “Free educts” represents Me_3SiH, H_2O and OY^- calculated separately. To be able to compare both OH^- and $OtBu^-$ on the same energetic scale, OH^- was chosen as the energetic reference and the reaction energy of HOtBu with OH^- was used to determine the energy of the free educts in the $OtBu^-$ case.

iv. Rationale for the promoting role of DMSO

As illustrated in the new **Figure S64**, we attribute the specific accelerating effect of DMSO to two primary factors:

- **High dissociating power (Figure S64-A):** As comprehensively detailed by Parker[43],[44], DMSO possesses a highly dissociating power. It efficiently breaks down the tetrameric KOtBu cluster by strongly solvating the K^+ cation, thereby unleashing a highly reactive, bare $^-$OtBu anionic nucleophile.
- **Stabilization of intermediates and transition states (Figure S64-B):** The dipolar aprotic nature of DMSO enables strong classical ion-dipole and dipole-dipole interactions, which, as the reviewer points out, could be expected to influence the reaction behavior.[45] Since this is an intriguing possible feature of the reaction, we had also already considered this hypothesis by investigating the influence of the experimentally most important solvents DMSO, ACN, THF and H_2O both in an implicit and explicit way (unpublished results). However, the results of this study are not yet conclusive as outlined below, thus not incorporated in the current study.

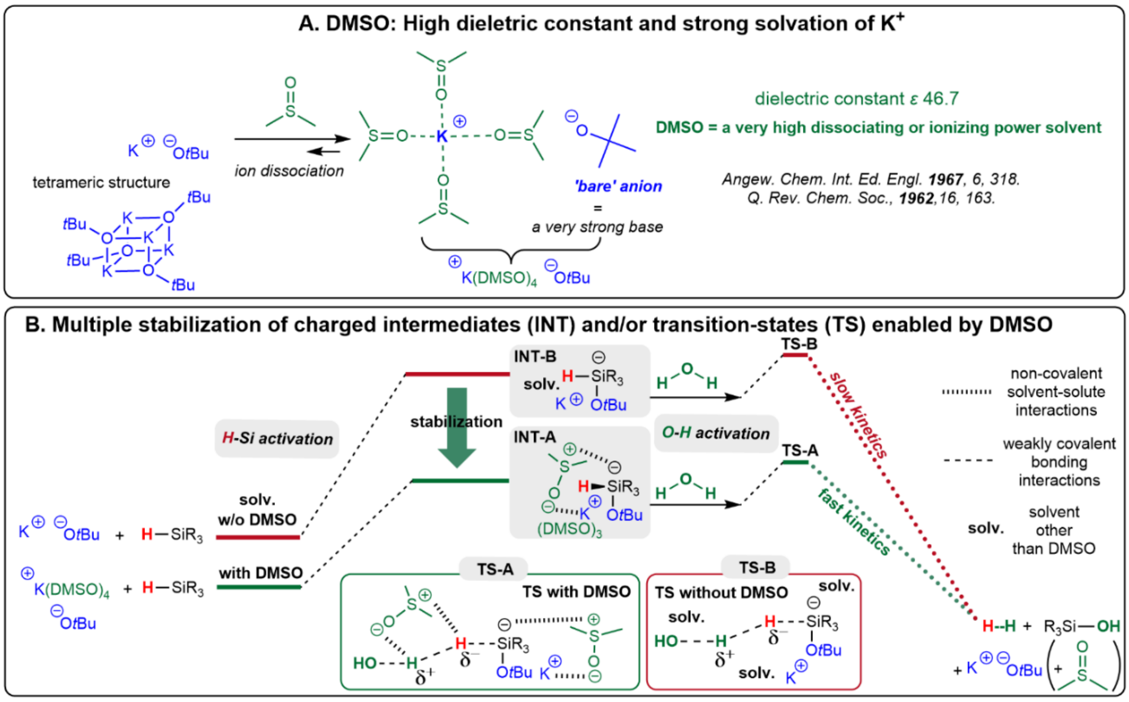

Figure S 64. Proposed rationale for the role of DMSO in the silane hydrolysis developed in this study. (A) Highly dissociating power of DMSO with respect to KOtBu. (B) Stabilizing solvation effects of DMSO lower the energy of intermediates and/or transition states that are proposed to arise from the Si–H activation and O–H activation steps.

To arrive at a holistic description of the reaction able to capture and explain all experimentally observed effects, we are currently working on a much more comprehensive description of our proposed mechanistic model by performing calculations of the full solvation shells of all species along the reaction coordinate. Because modeling the complete, dynamic explicit solvation shell across every intermediate of this complex system is a computationally non-trivial endeavor, a dedicated theoretical study is currently underway in our laboratory and will be reported in due course. **Due to its complexity, this study is, however, far outside the scope of the current manuscript.**

10. ***Hydrogen evolution profile for the controlled-addition experiment***

To investigate whether the intrinsic hyper-reactivity of our system could be modulated, we designed a preliminary continuous-addition experiment. We utilized a syringe pump to slowly feed 6.5 g of PMHS into the catalytic mixture (1 mL seawater, 62 mg KO*t*Bu, 2 mL DMSO) while cooling the reactor in a 15 °C thermostated bath.

This controlled-feed approach successfully suppressed the initial violent gas evolution, confirming the kinetics can be mechanically dampened (**Figure S65**). However, as the reaction progressed, the continuous formation of the solid siloxane network significantly increased the viscosity of the mixture. This likely hindered mass transfer, leading to an accumulation of unreacted PMHS followed by a late-stage kinetic acceleration (where nearly 1 L of H_2 was rapidly generated over the final ~2 minutes).

This experiment demonstrates a vital proof-of-concept: the H_2 evolution rate is not locked into a <60-second burst and can be modulated via reactant feed rates and thermal control. However, it also highlights that achieving a perfectly flat, steady-state hydrogen flow (suitable for fuel cells) requires dedicated chemical engineering optimizations, such as active, high-torque mechanical stirring or a continuous flow-through reactor design, to mitigate viscosity-induced mass transfer limitations and localized heating.

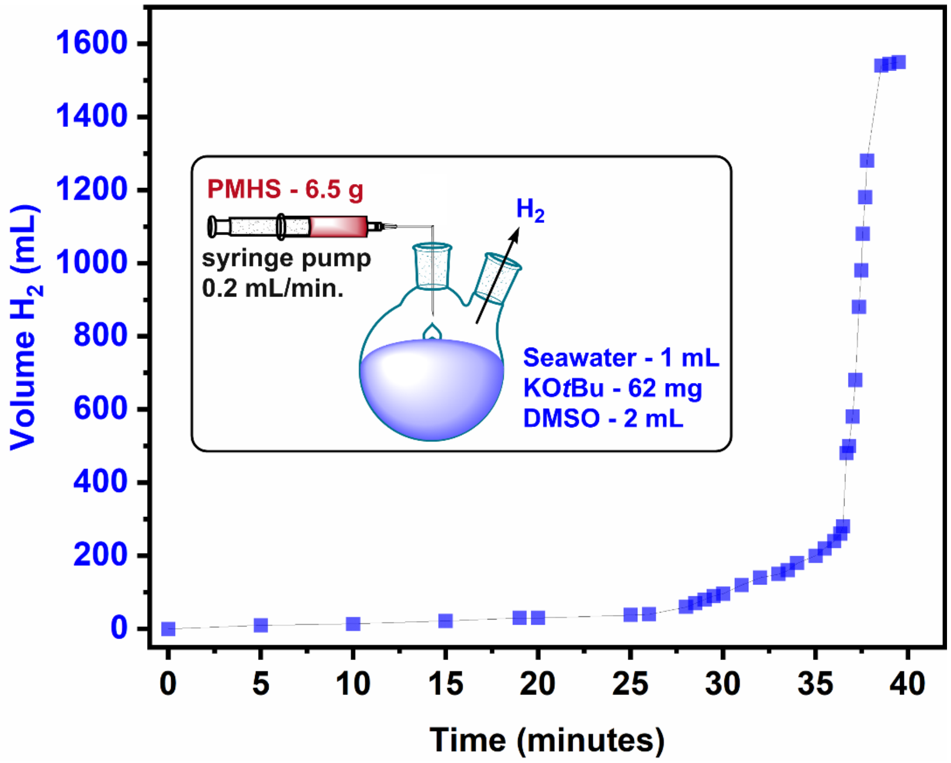

Figure S 65. Hydrogen evolution profile for the controlled-addition experiment. To modulate the release rate, PMHS (6.5 g) was continuously added via syringe pump (0.2 mL/min) to a catalytic mixture of seawater (1 mL), KOtBu (62 mg), and DMSO (2 mL) at 15 °C (inset). The continuous feed successfully suppressed the initial rapid gas evolution, though a late-stage kinetic acceleration was observed (at ~36 min) due to the increasing viscosity of the co-generated siloxane resin hindering mass transfer.

11. Control using different sources of seawater

As a preliminary study aiming at demonstrating the broader applicability of the system described herein, and in order to verify the possible influence of the type of seawater on the kinetics of hydrogen generation, we have collected two sources of Mediterranean seawater originating from Southern Europe (Croatia) and Northern Africa (Tunisia), two regions that should in principle contain enough compositional differences that potentially translate into differing chemical behavior. The results are shown in Figure S66 below. Only a slight difference in H_2 kinetics and H_2 yield could be seen between the two sources of seawater.

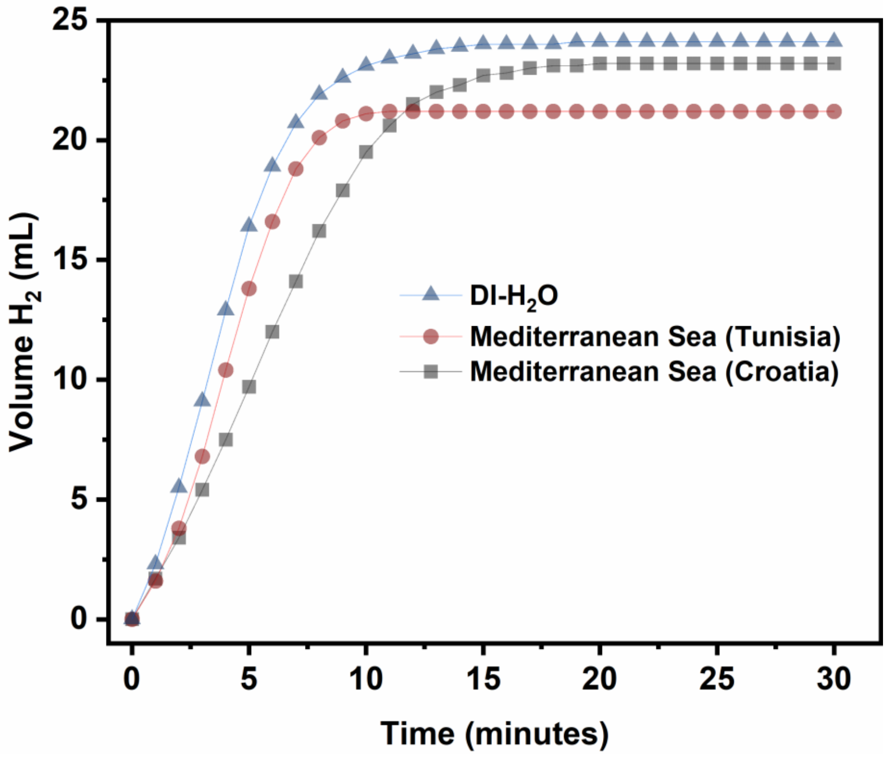

Figure S 66. Kinetic studies comparing of the silane hydrolysis reaction conducted under standard conditions (using DI-water) with the same reaction but conduced with two different sources of seawater: Mediterranean seawater from Croatia and Tunisia. Conditions: $HSiEt_3$ (129 mg, 1.11 mmol), H_2O (0.02 mL, 1.11 mmol), KOtBu (6.23 mg, 0.055 mmol), DMSO (0.5 mL), room temperature.

We conducted a series of kinetic experiments simulating the ionic environment of seawater. We added varying concentrations of NaCl to deionized water, as well as specific trials incorporating the hard divalent cations Mg^{2+} and Ca^{2+}. As shown in **Figure S67**, the kinetic profiles of these salt-spiked reactions are virtually superimposable with our standard deionized water conditions.

The initial burst kinetics and final H_2 volumetric yields remained unchanged. This demonstrates that the KOtBu/DMSO catalytic system is remarkably robust; the highly active silicate intermediate is not inhibited by high ionic strength, nor is it poisoned by competitive coordination or premature precipitation induced by hard, divalent spectator ions.

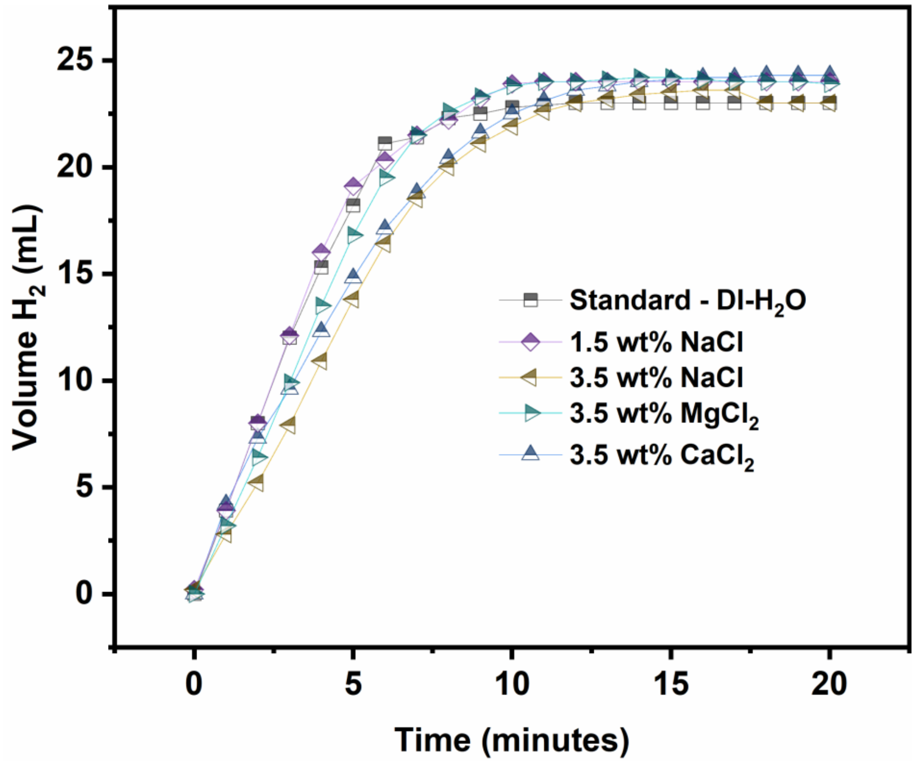

Figure S 67. Intentional addition of salts to DI-H_2O. Spike kinetic studies comparing of the silane hydrolysis reaction conducted under standard conditions (without salt addition, dark-blue line) with the same reaction but conduced with intentional addition of varying amounts of different salts to DI-H_2O (to mimic seawater). Conditions: $HSiEt_3$ (129 mg, 1.11 mmol), DI-H_2O (0.02 mL, 1.11 mmol), KOtBu (6.23 mg, 0.055 mmol), DMSO (0.5 mL), salt (1.5 or 3.5 wt% with respect to DI-H_2O), room temperature.

12. Recovery, reuse and recyclability

As explained in Scheme S1 below, we envisioned an integrated approach where the waste hydrosilane PMHS is valorized into H_2 using seawater while the silicone resin co-generated in the process is repurposed. Specifically, after the hydrogen generation step (step 1), the DMSO co-solvent was recycled via vacuum distillation (step 2) and reused in catalysis (step 3). Next, the recovered Si resin was purified to remove remaining KO*t*Bu (step 4) before repurposing via depolymerization (step 5) into Si monomers ($MeSiCl_3$ and $MeSiCl_2OH$).

Scheme S1. Summary of the integrated process

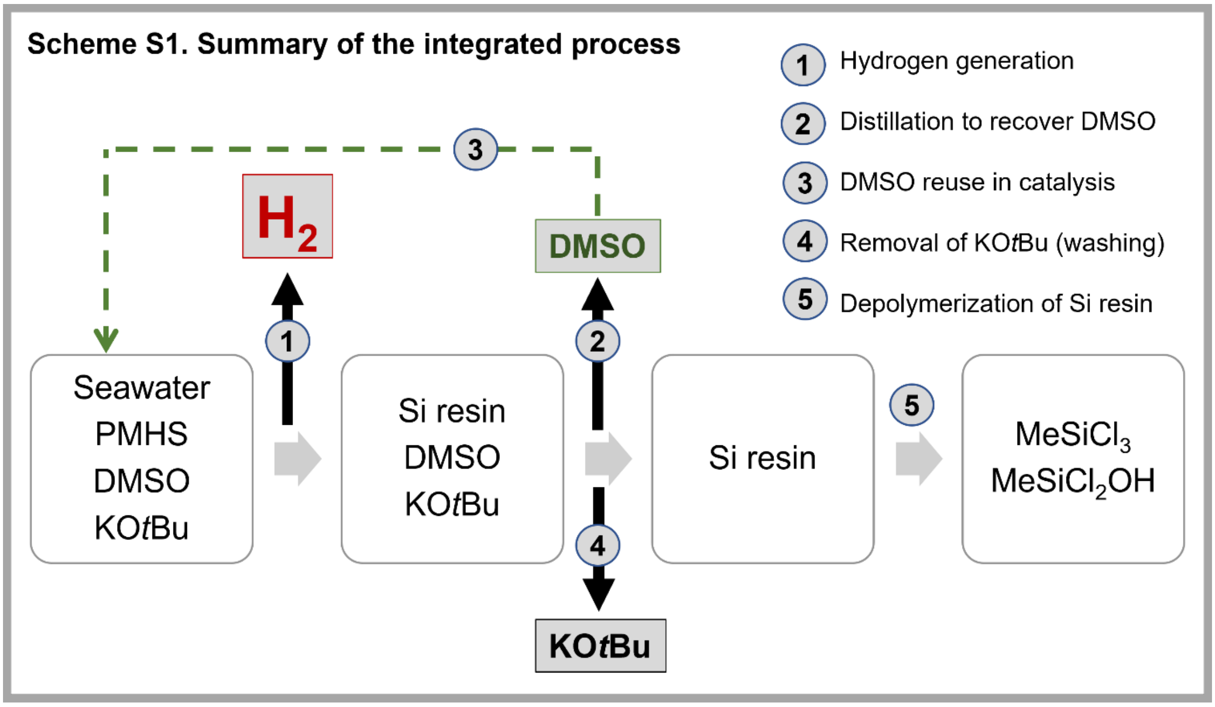

Procedure for hydrogen generation followed by distillation (steps 1 and 2; Scheme S1)

Following the general procedure, hydrogen was first generated from the mixture consisting of seawater (0.2 g, 11.1 mmol) and PMHS (1.3 g, 2 equiv.) in 1.1 g DMSO using KO*t*Bu (62 mg, 0.05 equiv.). A 25 mL flask was used. The rapid generation of hydrogen was accompanied by the formation of a white solid (Si Resin). The reaction mixture was directly submitted to bulb-to-bulb distillation using the Büchi Kugelrohr. The first fraction that was obtained was a clear liquid identified to be DMSO (0.95 g; 96% recovery) by ^{1}H NMR spectroscopy analysis in CD_3CN (see spectrum on Figure S68).

Procedure for reuse of DMSO (step 3; Scheme S1)

The recovered DMSO was applied in the same reaction as described above: seawater (0.2 g, 11.1 mmol), PMHS (1.3 g, 2 equiv.), 0.95 g DMSO, KO*t*Bu (62 mg, 0.05 equiv.). In less than one minute, >200 mL was generated; the rate of hydrogen generation was so rapid that no accurate measurement of H_2 volume versus time could be made.

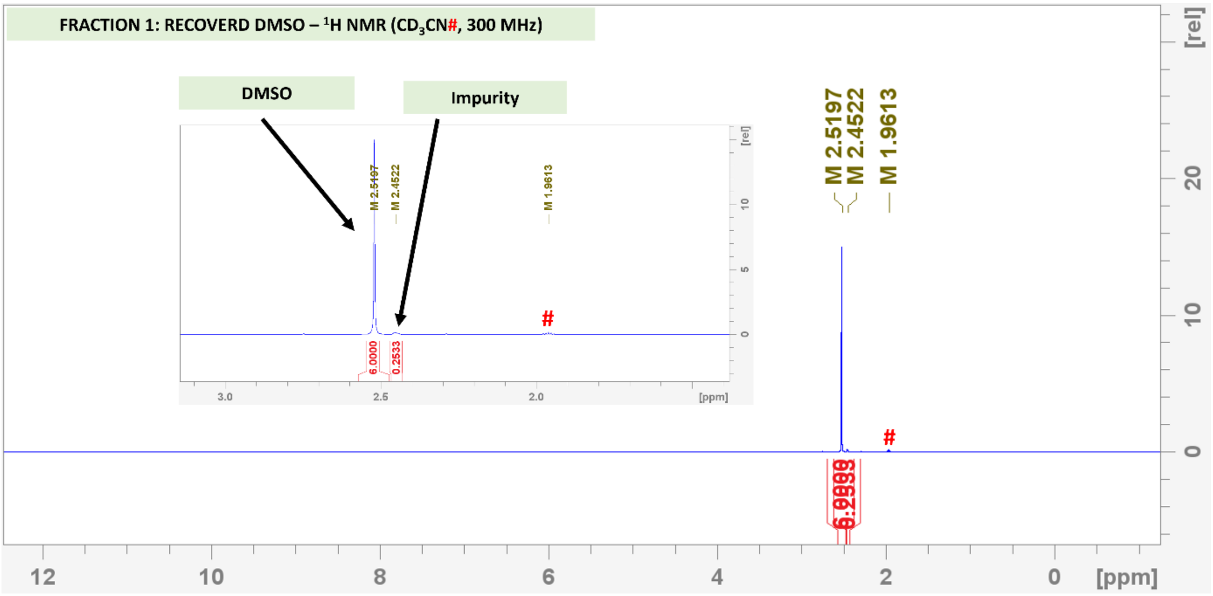

Figure S 68. Recovery of DMSO at the end of the reaction mixture between sea water and PMHS. General procedure for the hydrogen generation was used.

We performed additional experimental to demonstrate the robustness of the DMSO recycling (results complied in **Figure S69** below). The catalytic generation of hydrogen was conducted using our standard conditions (1.1 g DMSO, 0.2 g seawater, 62 mg KO*t*Bu, 1.3 g PMHS). After the first run, the DMSO was recovered via distillation. To account for minor mechanical/transfer losses inherent to distilling solvent at this small scale, a small amount of fresh DMSO was added to the recovered fraction to maintain the exact initial mass of 1.1 g for the subsequent runs, ensuring that the concentration and kinetic parameters remained perfectly comparable.

As shown in **Figure S69**, the hydrogen evolution profiles for three consecutive cycles are remarkably consistent. Both the initial kinetic rates (the slope of the V-t curve) and the final hydrogen yields are nearly identical across all three runs. This kinetic data definitively confirms that the DMSO co-solvent does not degrade or permanently deactivate during the highly basic catalytic cycle and can be easily recycled.

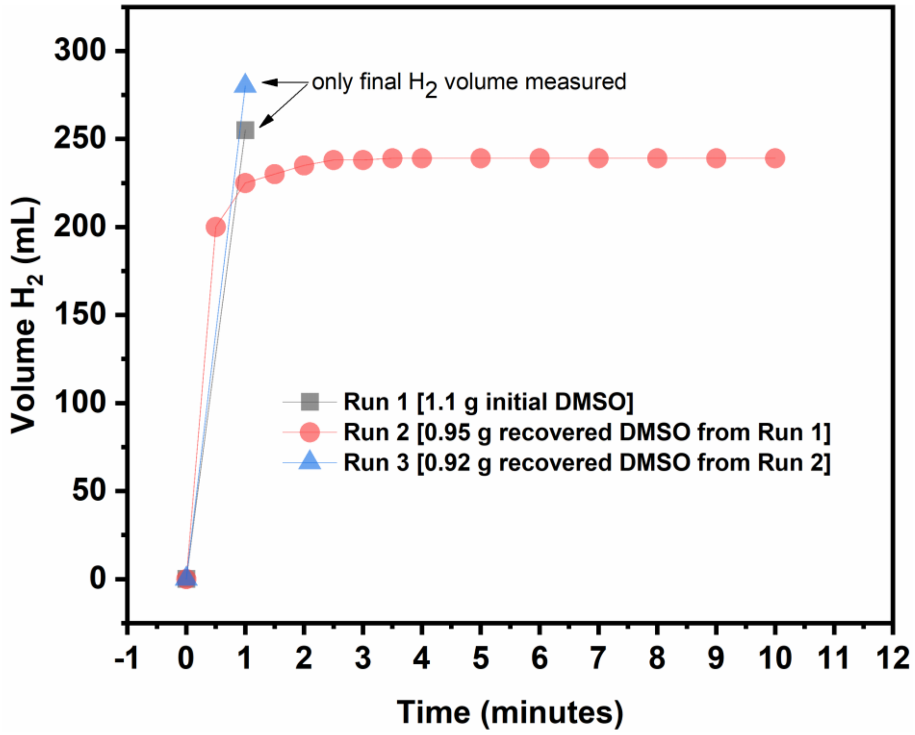

Figure S 69. Kinetic profiles of catalytic hydrogen evolution (H_2 Volume vs. time) over three consecutive cycles, demonstrating the robust recyclability of the DMSO co-solvent. Reaction conditions: 1.1 g DMSO, 0.2 g seawater, 62 mg KOtBu, 1.3 g PMHS; recovered DMSO was supplemented with trace fresh solvent to maintain a constant 1.1 g mass per cycle. Run 2: 0.95 g DMSO recovered (86%); Run 3: 0.92 g DMSO recovered (84%). The losses of DMSO are due to the rather viscous nature of DMSO making the recovery of traces DMSO ‘stuck’ along the walls of the distillation bulbs not trivial (to gain time, remaining DMSO was not recovered by extraction with another solvent).

Recovery of Si resin (step 4; Scheme S1)

The recovered Si resin was from the same hydrogen generation mixture that was used for recovering DMSO (see above). Reaction conditions: seawater (0.2 g, 11.1 mmol) and PMHS (1.3 g, 2 equiv.) in 1.1 g DMSO using KO*t*Bu (62 mg, 0.05 equiv.). After bulb-to-bulb distillation using the Büchi Kugelrohr, a white solid (Si resin) was left behind in the original flask of the reaction sample. This white solid was washed with DI-H_2O followed by stirring in MeOH at RT for 1 h, after which the suspension was filtered over a glass sintered funnel to recover a thin white powder that was further washed with DI-H_2O and MeOH before drying under vacuum at 80 °C for 1 h. Yield: 1.092 g (84% w/w with respect to starting PMHS).

Because the residual seawater ions (Na^+, Cl^-, Mg^{2+}, Ca^{2+}, SO_4^{2-}) and the KO*t*Bu catalyst are highly soluble in the aqueous/methanolic wash, they are efficiently extracted and removed from the insoluble silicon matrix. Therefore, these salts do not carry over to contaminate the solid resin, nor are they present to influence or interfere with the subsequent BCl_3-mediated depolymerization step.

The recovered Si resin was moderately soluble in chlorinated organic solvents ($CHCl_3$, CH_2Cl_2) but sparingly soluble in other organic solvent tested (e.g., DMSO, NCMe), and virtually insoluble in aqueous media. A ^{1}H NMR spectrum of the recovered resin was recorded in $CDCl_3$ (Figure S70).

NMR data of recovered Si resin

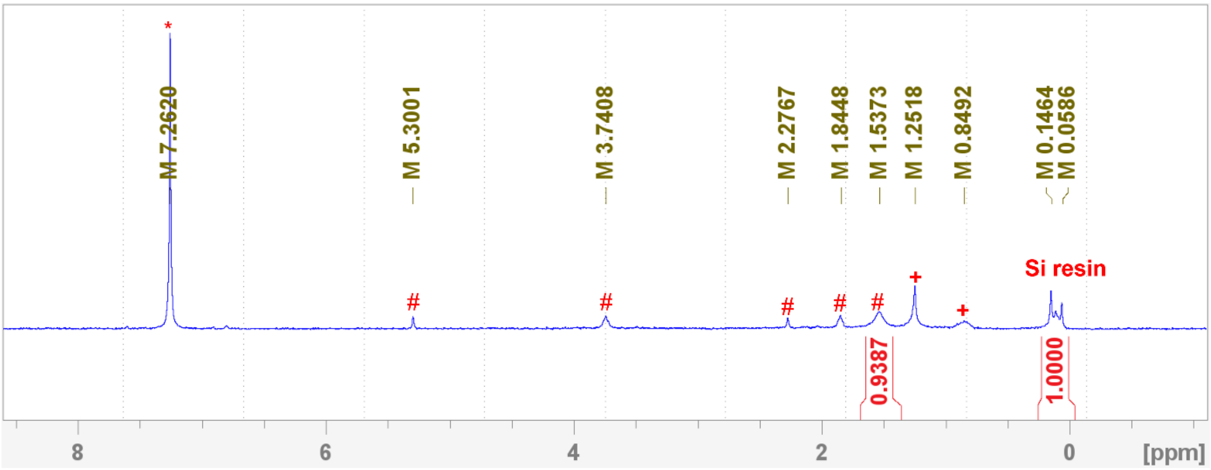

Figure S 70. ^{1}H NMR spectrum of the isolated Si resin. Solvent: $CDCl_3$(*). **#**: Residual solvent impurities (from left to right: CH_2Cl_2, THF, H_2O). **+**: Silicon grease. Spectrometer acquisition parameters: 300 MHz, 298 K.

Liquid-state NMR data ($CDCl_3$, 298 K, 300 MHz spectrometer). **^{1}H:** 0.15 ppm (s, Si-CH_3), 0.11/0.66/0.63 ppm (bs, presumably Si-CH_3 from smaller oligomers). **^{13}C{^{1}H}:** Despite the rather good solubility of the Si resin in chlorinated solvents, no ^{13}C resonance could be detected by ^{13}C {^{1}H} NMR (~4000 scan measurements). **^{29}Si:** Despite the good solubility of the Si resin in chlorinated solvents, no ^{29}Si resonance could be detected by ^{1}H-^{29}Si HSQC (~60 scan measurements).

Solid-state ^{29}Si NMR data. Due to the absence of any ^{29}Si resonance detectable by liquid-state NMR, the isolated Si resin was instead analyzed by solid-state ^{29}Si NMR using the technique of magic angle spinning (MAS). Details are given in the report below (Figure S71).[46] The observed resonance line at –65 ppm matches nicely with the resonance peak at ca. –65 ppm observed by liquid-state ^{29}Si NMR for the authentic Si resins that were independently synthesized by co-polymerization of $MeSiCl_3$ and water (cf. Supplementary Data 11-d). Further structural elucidation of the intermediate siloxane resin was attempted via solid-state 29Si CP-MAS NMR. Interestingly, no silicon signals could be detected, a physical phenomenon highly characteristic of a flexible, loosely crosslinked polymer gel. Because such mobile networks lack the structural rigidity required for effective cross-polarization, the signal remains severely attenuated. This physical profile, i.e., a flexible, lightly crosslinked oligomeric network rather than a dense, rigid T3 glass, provides a clear rationale for the resin's high solvent accessibility and its consequent rapid, high-yielding depolymerization by BCl_3 (vide infra).

REPORT

Solid-state ^{29}Si MAS NMR of polysiloxane (Si resin)

A. Marko, H.M.R. Wilkening, preliminary data

Institute for Chemistry and Technology of Materials, Graz University of Technology (NAWI Graz), Stremayrgasse 9, 8010 Graz (Austria)

Experimental. ^{29}Si (spin quantum number $I = 1/2$) magic angle spinning (MAS) NMR measurements were conducted using a 500 MHz Bruker Avance III spectrometer connected to a shimmed cryomagnet with a nominal magnetic field of 11.7 T. This field corresponds to a ^{29}Si Larmor frequency of 99.3 MHz. The polysiloxane powder was pressed into a 2.5 mm ZrO_2 MAS rotor. The sample was rotated at ambient bearing gas conditions at a spinning speed of 22 kHz. We acquired single pulse excitation experiments with a pulse length of 2 µs at a power level of 150 W to obtain 1D ^{29}Si MAS spectra after Fourier Transformation. The recycle delay was 500 s between each of the 50 scans. The chemical shifts were secondarily referenced to petalite (− 108(1) ppm). Zero-order and first-order phase corrections were applied to obtain pure absorption-mode signals.

Results. The ^{29}Si MAS NMR spectrum of the polysiloxane sample is shown in Fig. 1. A single broad Gaussian-shaped resonance line was observed at an isotropic chemical shift δ_{iso} of − 65 ppm. Signals at this shift are characteristic for T-type silicon sites, most likely a distribution of T^2 and T^3 local environments, that is, R−Si(OSi)$_2$(OR) and R−Si(OSi)$_3$ units, respectively.[1] These sites are typically found in highly crosslinked or condensed regions of polysiloxane networks.

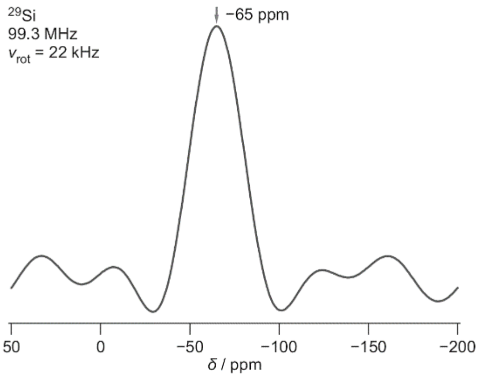

Figure 1: ^{29}Si MAS NMR spectrum (99.3 MHz, 22 kHz spinning speed) of the polysiloxane sample. The observed line at −65 ppm can be attributed to T^2 or T^3 local silicon environments, or more likely a distribution of both types arising from partial to full condensation within the polysiloxane network.

References

(1) Kalfat, R.; Babonneau, F.; Gharbi, N.; Zarrouk, H. *J. Mater. Chem.* **1996**, *6*, 1673−1678.

Figure S 71. Analysis of the isolated sample of Si Resin by solid-state ^{29}Si NMR using the technique of magic angle spinning (MAS).

Preparation of the authentic T-type polysiloxanes (samples LargeMW and SmallMW)

Two silicone resins were prepared and isolated using an adapted procedure by Itoh and coworkers.[47] In short, a mixture of 45 ml MIBK and 60 ml deionized water was cooled to 5–10 °C. Then, 22.4 g of methyltrichlorosilane ($MeSiCl_3$) were dissolved in 15 ml of methylisobutylketone (MIBK) and the solution was added dropwise to the MIBK/water mixture, with the temperature of the reaction always remaining between 0 and 10 °C. After complete addition, the reaction was slowly heated to 50 °C for 1 hour and then stirred at the same temperature for 2.5 hours. The organic phase was washed with deionized water and centrifuged to collect any insoluble material. The precipitate was washed with MIBK and

acetone and then dried under high vacuum for 24 h@RT (sample labelled as '**LargeMW**'; m = 0.2011 g). The supernatant was combined with the MIBK used for washing the precipitate, the solvents evaporated to dryness using the rotavapor and the resulting residue dried under high vacuum vacuum for 24 h@RT (**sample labelled as 'SmallMW'**; m = 5.630 g). NMR data of both isolated samples **LargeMW** and **SmallMW** match those reported by Itoh and coworkers[47].

NMR data for the isolated sample SmallMW

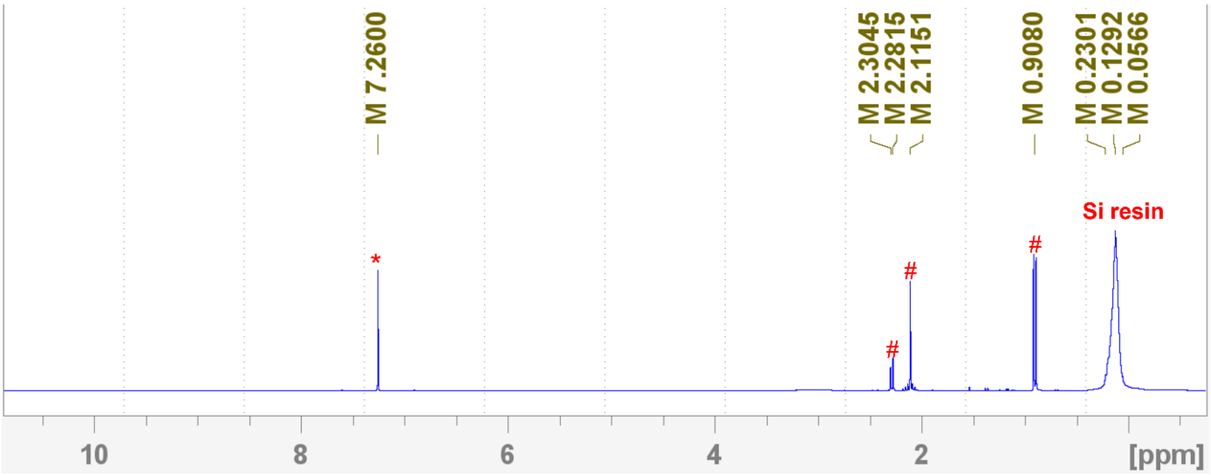

Figure S 72. ^{1}H NMR spectrum of the isolated Si resin '**SmallMW**'. $CDCl_3$(*). **#**: Residual solvent impurities (MIBK). Spectrometer acquisition parameters: Field at 300 MHz, 298 K.

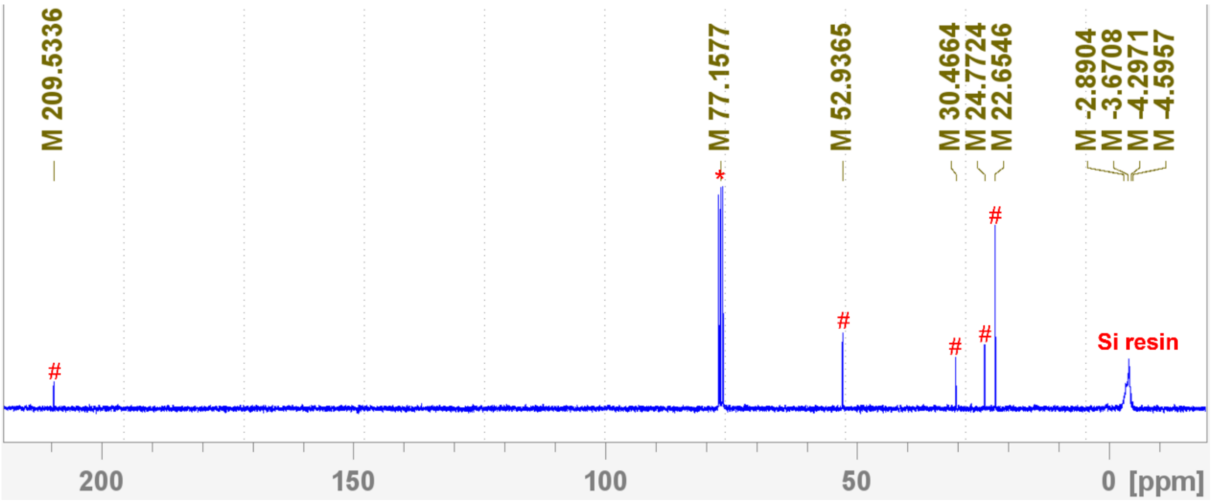

Figure S 73. $^{13}C\{^{1}H\}$ NMR spectrum of the isolated Si resin '**SmallMW**'. $CDCl_3$(*). **#**: Residual solvent impurities (MIBK). Spectrometer acquisition parameters: Field at 300 MHz (^{13}C resonance at 75.5 MHz), 298 K.

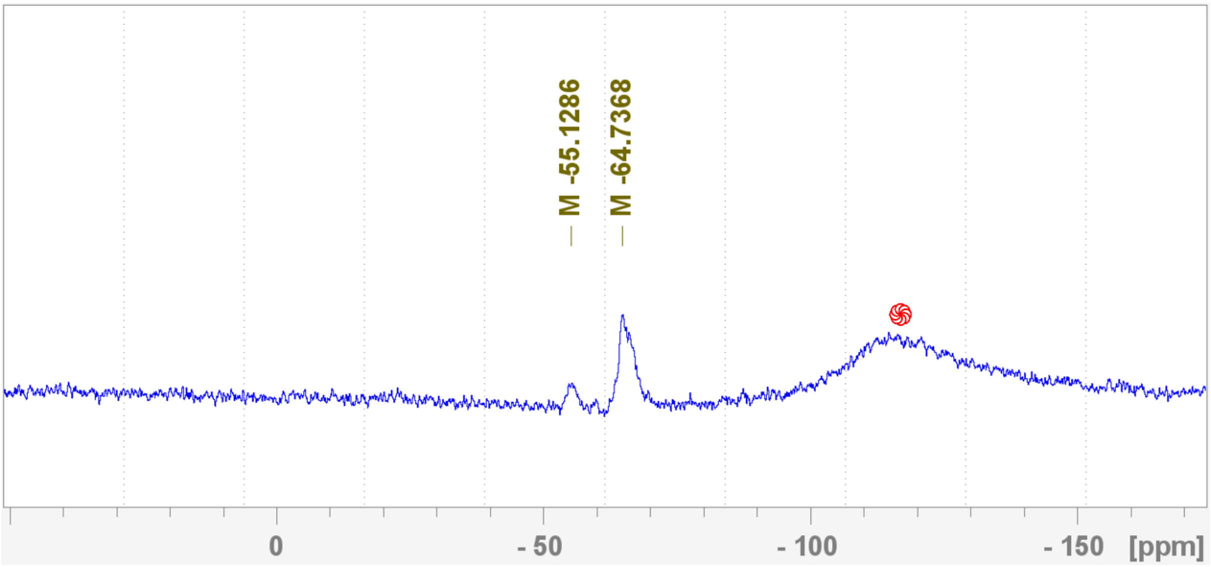

Figure S 74. $^{29}Si\{^1H\}$ NMR spectrum of the isolated Si resin '**SmallMW**'. Solvent: $CDCl_3$. ❂: Background Si signal from the borosilicate of the NMR tube. Spectrometer acquisition parameters: Field at 300 MHz (^{29}Si resonance at 59.6 MHz), 298 K.

NMR data in ppm for SmallMW ($CDCl_3$, 298 K, 300 MHz spectrometer): ^{1}H 0.23-0.06 (br, Si-CH_3); **$^{13}C\{^1H\}$ (75.5 MHz)** –2.6-4.5 (br, Si-CH_3). **^{29}Si (59.6 MHz)** –55.1 (br, Si-CH_3), –64.7 (br, Si-CH_3).

NMR data for the isolated sample LargeMW

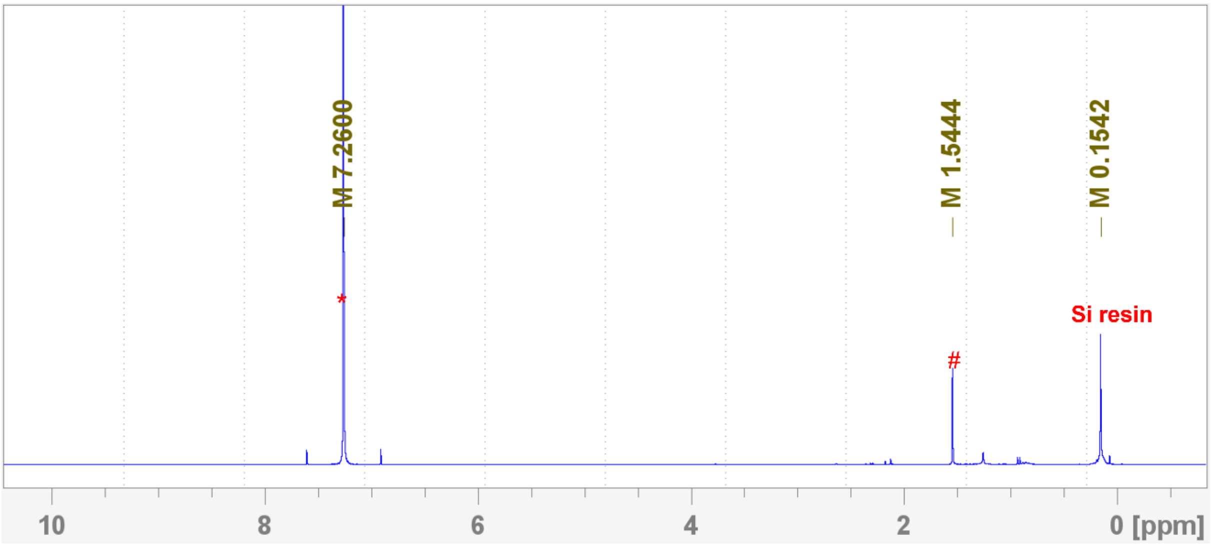

Figure S 75. ^{1}H NMR spectrum of the isolated Si resin '**LargeMW**'. $CDCl_3$(*). **#**: Residual solvent impurities (water). Spectrometer acquisition parameters: Field at 300 MHz, 298 K.

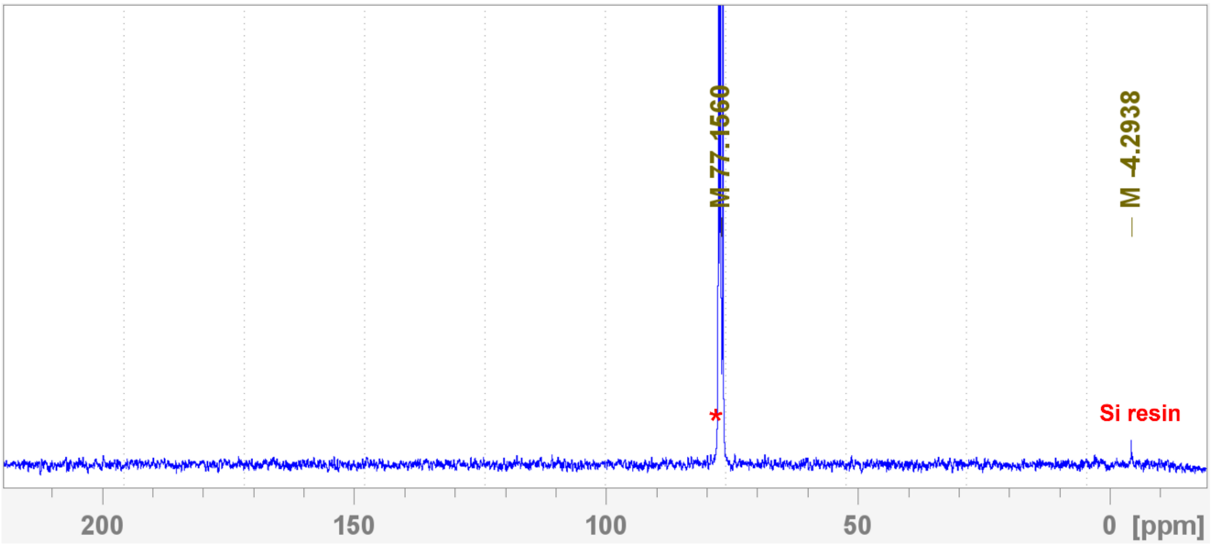

Figure S 76. $^{13}C\{^{1}H\}$ NMR spectrum of the isolated Si resin '**LargeMW**'. $CDCl_3$(*). Spectrometer acquisition parameters: Field at 300 MHz (^{13}C resonance at 75.5 MHz), 298 K.

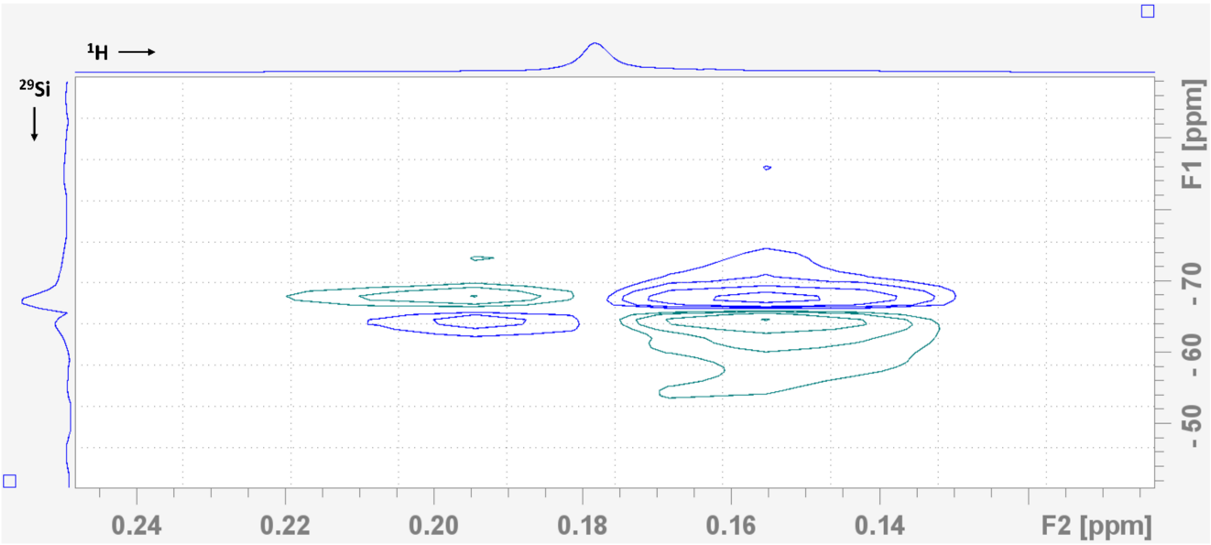

Figure S 77. ^{1}H-^{29}Si HSQC NMR spectrum of the isolated Si resin '**SmallMW**'. Solvent: $CDCl_3$. Spectrometer acquisition parameters: Field at 300 MHz (^{29}Si resonance at 59.6 MHz), 298 K.

NMR data in ppm for LargeMW ($CDCl_3$, 298 K, 300 MHz spectrometer): ^{1}H (300 MHz) 0.15 (s, Si-CH_3); **$^{13}C\{^{1}H\}$ (75.5 MHz)** –4.3 ppm (s, Si-CH_3). **^{1}H-^{29}Si (300/59.6 MHz)** –65.0 (br, Si-CH_3), –66.0 (br, Si-CH_3).

IR data comparison of recovered Si resin with the samples LargeMW and SmallMW

The Si resin sample isolated from the hydrogen generation studies was further analyzed by Infrared spectroscopy, and compared with the independently synthesized silicone resins **LargeMW** and **SmallMW**. The results depicted in Figure S78 below confirm the structure of a cross-linked polymethylsilsesquioxane for the Si resin mainly consisting of T^3 [$MeSiO_{3/2}$] units.

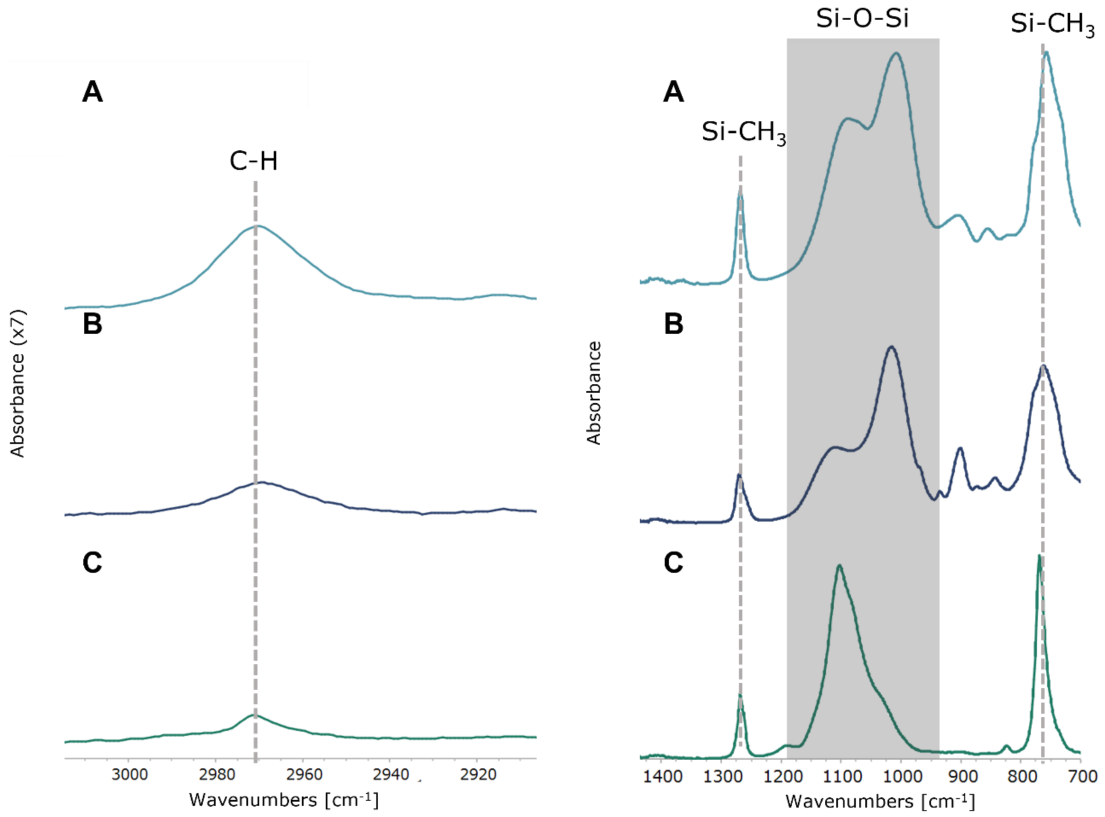

Figure S 78. IR spectra of the isolated silicone resins (**A**) **SmallMW**, (**B**) **Si resin** and (**C**) **LargeMW**.

Specifically, all three samples display peaks related to Si-CH_3 bond vibrations at around 1270 and 760 cm^{-1}, and the corresponding C-H bond vibrations can be seen at 2970 cm^{-1}. These vibrations are characteristic of the T^3 [$MeSiO_{3/2}$] units.[48]

In all samples analyzed, the answer to the question of open or cage-like structure for the polymethylsilsesquioxane lies in the region 1200–1000 cm^{-1}. At these wavenumbers, short ladder structures, open cages and cages with uneven numbers of Si-O units all show a double peak for Si-O-Si bond vibrations. These bands refer to asymmetric and symmetric modes of stretching vibrations, and can be seen in Figure S57-A at ~1088 cm^{-1} and ~1008 cm^{-1}, respectively.[48]

Cage structures with an even number of Si-O units, however, only show a peak for the asymmetric vibration mode. This indicates the presence of such structure in the **LargeMW** resin (Figure S78-C). The higher relative peak intensity of the symmetric mode in our original Si resin (Figure S78-B) can be linked to a more random network structure than for the **SmallMW** resin.

Depolymerization studies of the Si resin using method A (step 5; Scheme S1)

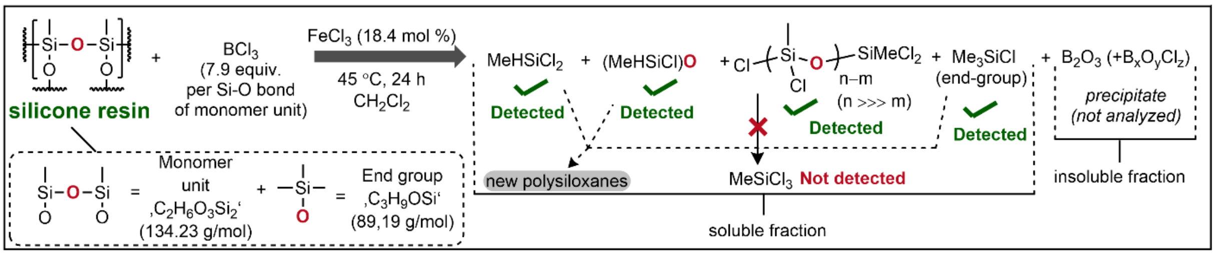

Procedure for the depolymerization of the silicone resin (method A: $BCl_3/FeCl_3$[49]). **Inside the glovebox,** in a dry head-space 20-mL vial charged with a stir bar was added 50 mg of the Si resin (0.37 mmol based on the repeating monomer unit assumed to be '$(CH_3)_2O_3Si_2$' and ignoring the end group unit '$(CH_3)_3SiO$'), 11.0 mg $FeCl_3$ (0.068 mmol, 18.4 mol %) and 1 mL dry CH_2Cl_2. Then BCl_3 in CH_2Cl_2 2.5 M (3.5 mL, 8.75 mmol, 7.9 equiv. per Si-O bond of monomer unit) was added portion-wise. The vial was tightly sealed to prevent the evaporation of the solvent and BCl_3. The final volume solution was ca. 3.5 mL. Stirring for ca. 24 h gave a clear yellowish solution with a white precipitate (B_2O_3 and $B_xO_yCl_z$). After this time, the vial was cooled to room temperature. For the determination of relative molar ratio by NMR, mesitylene was directly added to the rection mixture by mass (100.1 mg), then ca. 0.35 mL aliquot was taken for NMR analysis in $CDCl_3$ (0.3 mL added) using a J-Young tube. A very small amount of solid slowly deposited inside the tube.

NMR data confirmed the extensive but not complete depolymerization of the Si resin (as ascertained by the disappearance of the ^1H and ^{29}Si resonances at 0.15 and –65 ppm, respectively). The resultant mixture consisted of the Si species $MeHSiCl_2$, $(MeHSiCl)_2O$, Me_3SiCl as the main monomers identified, as well as the presumed $Cl(MeSiCl_2O)_mSiMeCl_2$ as the major dimeric/oligomeric Si species (≥50% based on NMR integrations). Other unknow lower molecular weight Si dimer/oligomers could not be unambiguously identified.

All attempts to identify the presence of the expected $MeSiCl_3$ were unsuccessful. It was previously shown that the presence of a Re catalyst supported on CeO_2 enabled the conversion of $MeSiCl_3$ more readily into branched cyclosiloxanes.[50] Hence, we can also assume a similar phenomenon occurring in the depolymerization mixture whereby $FeCl_3$ could catalyze the transformation of $MeSiCl_3$ into siloxane dimers or oligomers.

Another more viable explanation for the absence of formation of $MeSiCl_3$ is the inability of the $BCl_3/FeCl_3$ catalytic system for cleaving the cross-linked Si-O bond, which is expected to be less physically accessible and/or chemically reactive. To address this issue, we have applied modification to the $BCl_3/FeCl_3$ catalysis mixture whereby catalytic amounts of $AlCl_3$ were added (cf. next section for results).

NMR quantification. The molar ratio M_x/M_y (Eq. S1) between two identified analytes x and y was determined using the known concentration of cyclohexane as reference compound (internal standard). The results are provided in Table S8.

$\frac{M_x}{M_y} = \frac{I_x}{I_y} \times \frac{N_y}{N_x}$ (Eq. S1), where *I* is the integral and *N* is the number of nuclei giving rise to the signal.

Table S 8. NMR quantification using Eq. S1.

Compounds	Molar ratio
$MeHSiCl_2$:$(MeHSiCl)_2O$	1:0.25
$MeHSiCl_2$:Me_3SiCl	1:0.35

NMR data in ppm of the mixture from method A

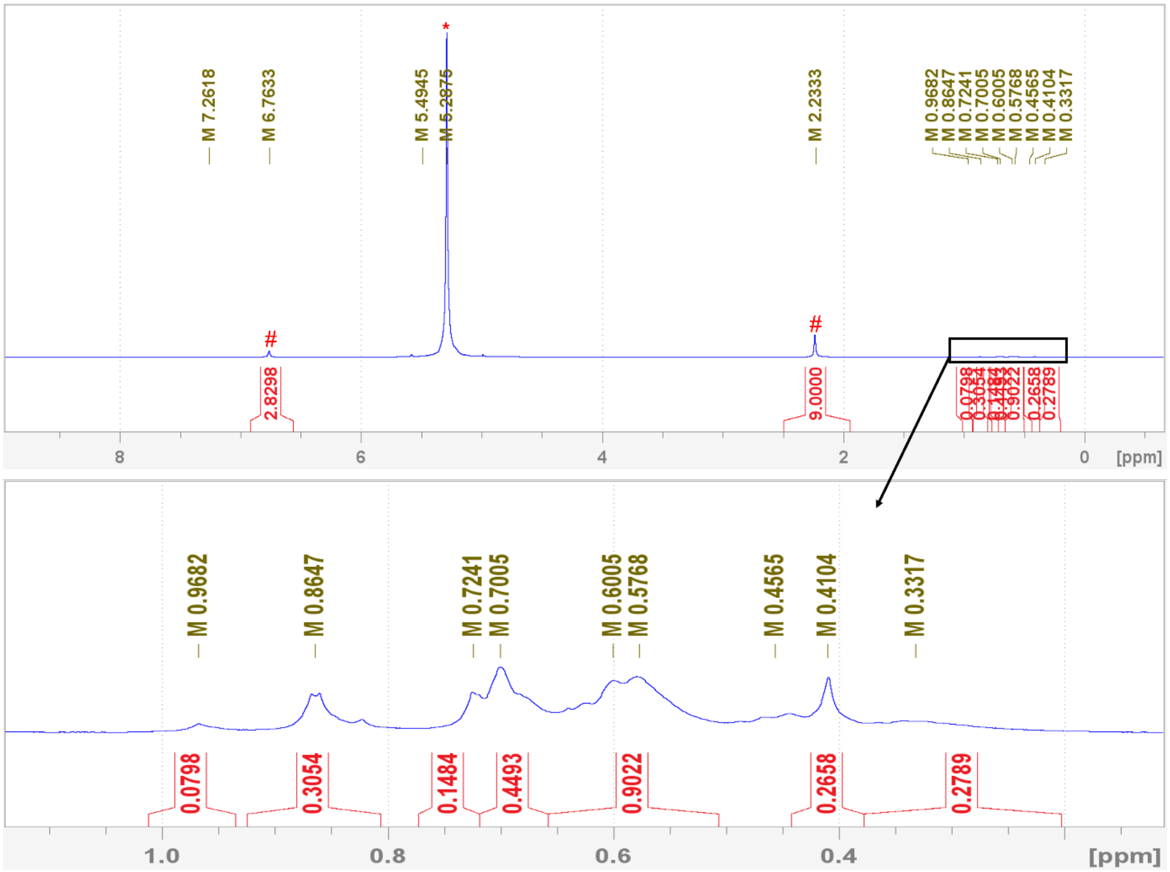

Figure S 79. ^{1}H NMR spectrum of the reaction mixture resulting from the Si resin depolymerization using Method A ($BCl_3/FeCl_3$@cat./CH_2Cl_2/45C@24h). Solvent mixture: $CDCl_3/CH_2Cl_2$(*) in ca. 1:1 v/v. Internal standard: 100.1 mg of mesitylene (#, 0.832 mmol). Spectrometer acquisition parameters: 300 MHz, 298 K.

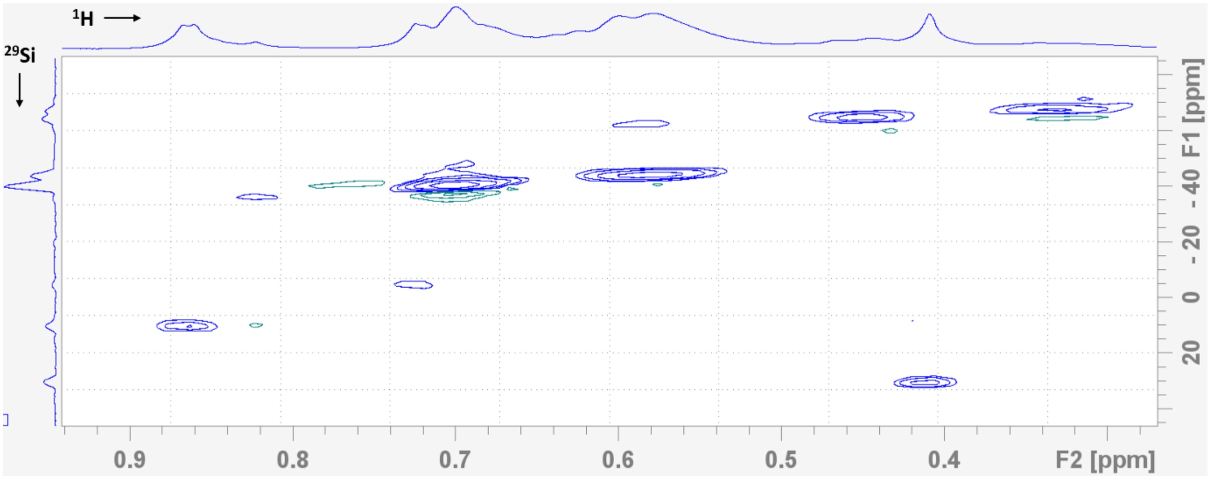

Figure S 80. ^{29}Si NMR spectrum of the reaction mixture resulting from the Si resin depolymerization using Method A ($BCl_3/FeCl_3$@cat./CH_2Cl_2/45C@24h). Solvent mixture: $CDCl_3/CH_2Cl_2$ in ca. 1:1 v/v. Spectrometer acquisition parameters: 300/59.6 MHz, 298 K.

^{1}H ($CDCl_3$, 300 MHz, 298 K). $MeHSiCl_2$[51]: 5.56 (H, overlaps with C*H*DCl_2), 0.86 (d, $^{3}J_{H\text{-}H}$= 2 Hz, Me); $(MeHSiCl)_2O$[52]: 5.42 (H, overlaps with C*H*DCl_2), 0.72 (d, $^{3}J_{H\text{-}H}$= 2 Hz, Me); Me_3SiCl[49]: 0.41 (s, Me); $Cl(MeSiCl_2O)_mSiMeCl_2$: 0.70–0.33 (br, Me). **^{1}H-^{29}Si NMR data in ppm ($CDCl_3$, 300/59.6 MHz, 298 K).** $(MeSiCl_2)_2$[53]: 10.5; $(MeHSiCl)_2O$[47]: –4.5; Me_3SiCl: 30.6; $Cl(MeSiCl_2O)_mSiMeCl_2$[49] (+unknown): –40.4, –44, –62.1, –64.7, –67.6.

Depolymerization studies of the Si resin using new method B (step 5; Scheme S1)

A modification was made to method A originally reported by Vu an coworkers[49], which led to the exhaustive depolymerization of the Si resin (see details below).

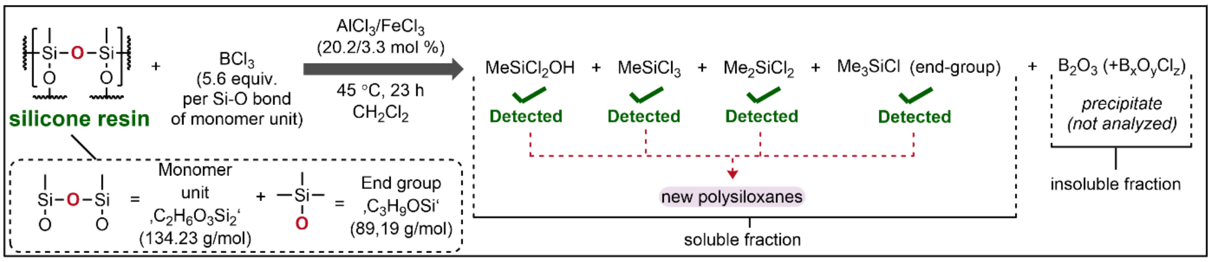

Procedure for the depolymerization of the silicone resin (method B: BCl_3/$AlCl_3$/$FeCl_3$). **Inside the glovebox,** in a dry head-space 20-mL vial charged with a stir bar was added 50 mg of the Si resin (0.37 mmol based on the repeating monomer unit assumed to be '$(CH_3)_2O_3Si_2$' and ignoring the end group unit '$(CH_3)_3SiO$'), 10.0 mg anhydrous $AlCl_3$ (0.075 mmol, 20.2 mol %), 2.0 mg anhydrous $FeCl_3$ (0.012 mmol, 3.3 mol %) and 1 mL dry CH_2Cl_2. Then BCl_3 in CH_2Cl_2 2.5 M (2.5 mL, 6.25 mmol, 5.6 equiv. per Si-O bond of monomer unit) was added portion-wise. The vial was tightly sealed to prevent the evaporation of the solvent and BCl_3. The final volume solution was ca. 3.5 mL. Stirring for ca. 22 h gave a clear yellowish solution with very little white precipitate noticeable (B_2O_3 and $B_xO_yCl_z$). After this time, the vial was cooled to room temperature. For the determination of relative molar ratio by NMR, cyclohexane was directly added as internal standard to the rection mixture by mass (46.3 mg), then ca. 0.35 mL aliquot was taken for NMR analysis in $CDCl_3$ (0.3 mL added) using a J-Young tube. A very small amount of solid slowly deposited inside the tube.

NMR data confirmed the complete depolymerization of the Si resin (as ascertained by the disappearance of the ^{1}H and ^{29}Si resonances at 0.15 and –65 ppm, respectively). The resultant mixture consisted mainly of the monomeric Si species $MeSiCl_3$, $MeSiCl_2OH$, Me_2SiCl_2 and Me_3SiCl. The dimeric/oligomeric Si species $Cl(MeSiCl_2O)_mSiMeCl_2$ previously observed with Method A were now not detected. The known Si dimer $(MeSiCl_2)_2O$ was also detected, although in very limited amount (see Table S9). The depolymerization of the authentic sample **SmallMW** using similar catalytic conditions as those employed here (method B) for the Si resin led to a similar depolymerization mixture, as ascertained by the ^{1}H and ^{1}H-^{29}Si NMR spectra depicted in Figures S81–82.

NMR quantification. The molar ratio M_x/M_y (Eq. S1) between two identified analytes x and y was determined using the known concentration of cyclohexane as reference compound (internal standard). The results are provided in Table S9.

$\frac{M_x}{M_y} = \frac{I_x}{I_y} \times \frac{N_y}{N_x}$ (Eq. S1), where *I* is the integral and *N* is the number of nuclei giving rise to the signal.

Table S 9. NMR quantification using Eq. S1.

Compounds	Molar ratio
$MeSiCl_2OH$:$MeSiCl_3$	1:0.40
$MeSiCl_2OH$:Me_2SiCl_2	1:0.06
$MeSiCl_2OH$:Me_3SiCl	1:0.07
$MeSiCl_2OH$:$(MeSiCl_2)_2O$	1:0.12

NMR data in ppm of the mixture from method B

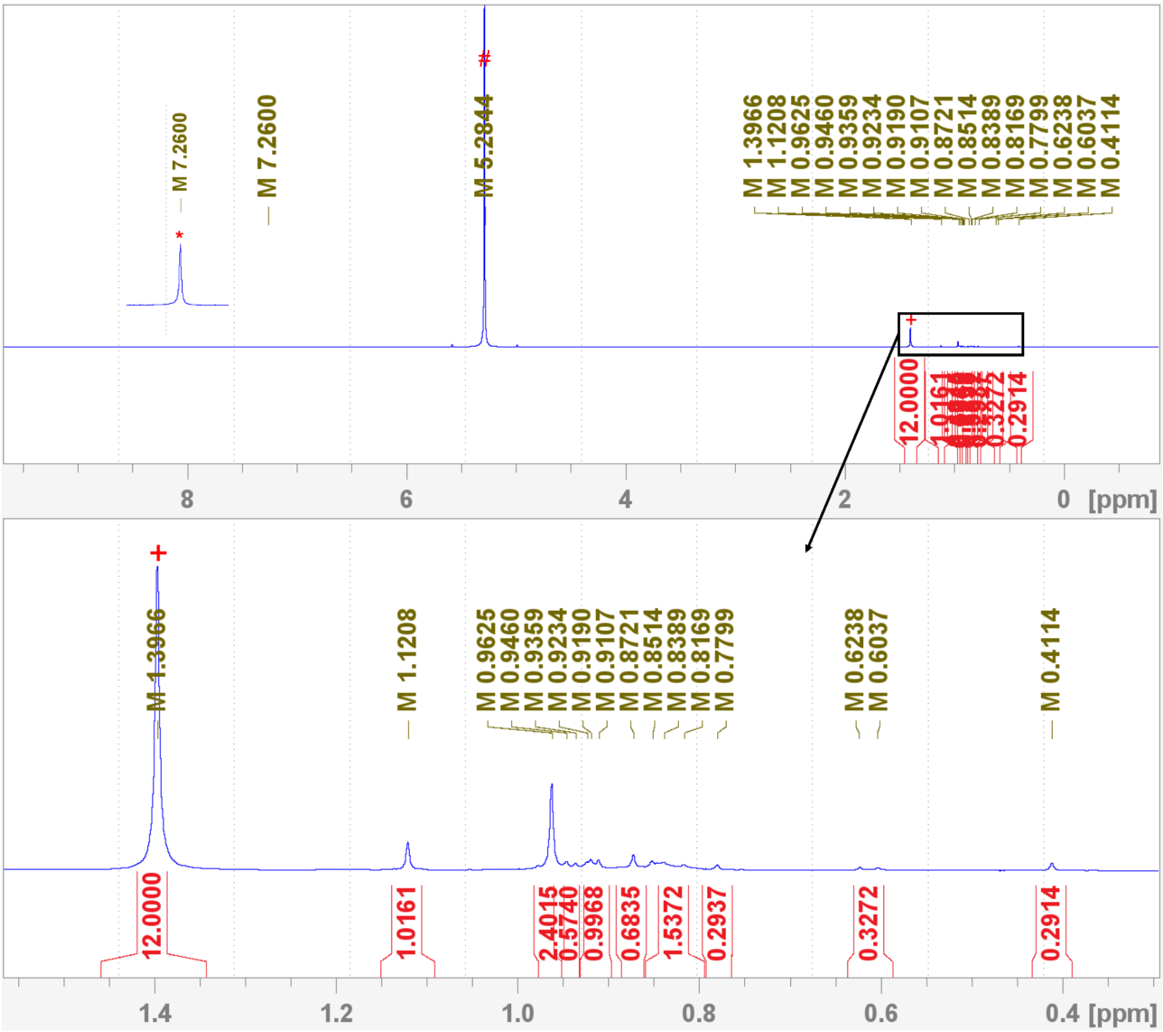

Figure S 81. ^{1}H NMR spectrum of the reaction mixture resulting from the Si resin depolymerization using Method B ($BCl_3/AlCl_3/FeCl_3$@cat./CH_2Cl_2/45C@22h). Solvent mixture: $CDCl_3$(#)/CH_2Cl_2(*) in ca. 1:1 v/v. Internal standard: 46.3 mg of cyclohexane (+) (overall integration amounts to 0.55 mmol). Spectrometer acquisition parameters: 300 MHz, 298 K.

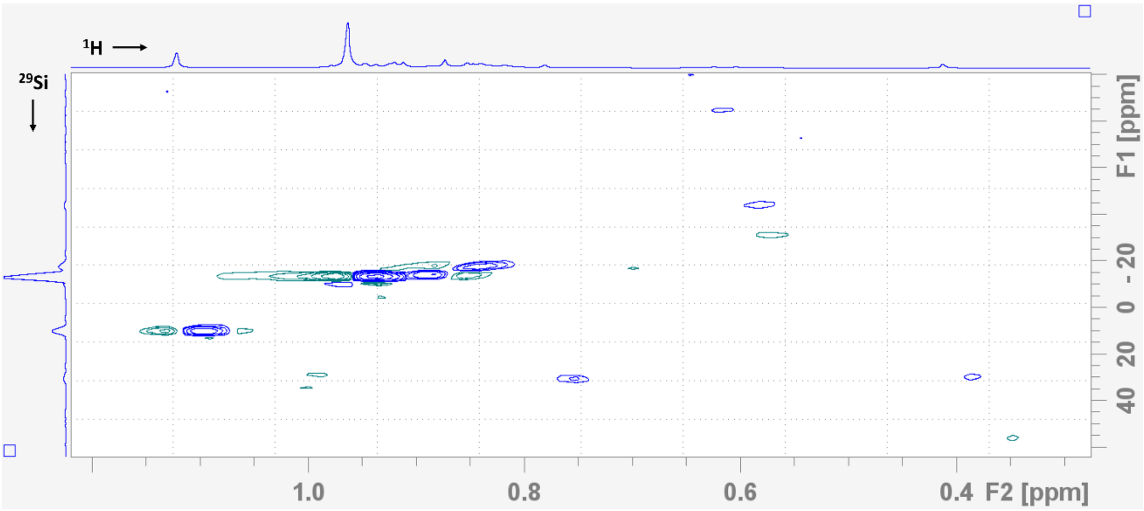

Figure S 82. ^{29}Si NMR spectrum of the reaction mixture resulting from the Si resin depolymerization using Method B ($BCl_3/AlCl_3/FeCl_3$@cat./CH_2Cl_2/45C@22h). Solvent mixture: $CDCl_3/CH_2Cl_2$ in ca. 1:1 v/v. Spectrometer acquisition parameters: 300/59.6 MHz, 298 K.

^{1}H (CDCl$_3$, 300 MHz, 298 K). $MeSiCl_3$[54]: 2.12 (s, Si-Me); $MeSiCl_2OH$[51]: 0.96 (bs, Si-Me); $(MeSiCl_2)_2O$[55]: 0.87 (bs, Si-Me); Me_2SiCl_2[49]: 0.78 (s, Me); Me_3SiCl[49]: 0.41 (s, Me). **^{1}H-^{29}Si NMR data in ppm (CDCl$_3$, 300/59.6 MHz, 298 K).** Me_2SiCl_2[49]: 31.9; Me_3SiCl[56]: 30.9; $MeSiCl_3$[49]: 11.2; $MeSiCl_2OH$[51]: –12.1; $(MeSiCl_2)_2O$[55]: –16.3; unknown (minor): –42.5.

Depolymerization of the prepared authentic sample SmallMW using method B

A modification was made to method A originally reported by Vu an coworkers[49], which led to the quasi-exhaustive depolymerization of the Si resin (see details below).

Procedure for the depolymerization of the silicone resin (method B: $BCl_3/AlCl_3/FeCl_3$). **Inside the glovebox,** in a dry head-space 20-mL vial charged with a stir bar was added 50 mg of **SmallMW** (0.37 mmol based on the repeating monomer unit assumed to be '$(CH_3)_2O_3Si_2$'; no end group unit '$(CH_3)_3SiO$'), 10.0 mg anhydrous $AlCl_3$ (0.075 mmol, 20.2 mol %), 2.0 mg anhydrous $FeCl_3$ (0.012 mmol, 3.3 mol %) and 1 mL dry CH_2Cl_2. Then BCl_3 in CH_2Cl_2 2.5 M (2.5 mL, 6.25 mmol, 5.6 equiv. per Si-O bond of monomer unit) was added portion-wise. The vial was tightly sealed to prevent the evaporation of the solvent and BCl_3. The final volume solution was ca. 3.5 mL. Stirring for ca. 22 h gave a clear brown-yellowish solution with very little white precipitate noticeable (B_2O_3 and $B_xO_yCl_z$). After this time, the vial was cooled to room temperature. For the determination of relative molar ratio by NMR, cyclohexane was directly added as internal standard to the rection mixture by mass (44.3 mg), then ca. 0.35 mL aliquot was taken for NMR analysis in $CDCl_3$ (0.3 mL added) using a J-Young tube. A very small amount of solid slowly deposited inside the tube.

NMR data confirmed the quasi-complete depolymerization of the sample **SmallMW** (as ascertained by the disappearance of the ^1H and ^{29}Si resonances at 0.13 and –55/65 ppm, respectively). The resultant mixture consisted mainly of the monomeric Si species $MeSiCl_3$, $MeSiCl_2OH$, Me_2SiCl_2 and Me_3SiCl. The dimeric/oligomeric Si species $Cl(MeSiCl_2O)_mSiMeCl_2$ previously observed with Method A were now not detected. The known Si dimer $(MeSiCl_2)_2O$ was also detected, although in very limited amount (see Table S10). The depolymerization of the mixture thus obtained for the sample **SmallMW** is very similar to the depolymerization mixture observed above for the sample of Si resin; in fact, the resultant ^1H and ^1H-^{29}Si NMR spectra are almost identical in both cases. However, there still remained some higher oligomers in the present mixture (Figures S83-S84).

NMR quantification. The molar ratio M_x/M_y (Eq. S1) between two identified analytes x and y was determined using the known concentration of cyclohexane as reference compound (internal standard). The results are provided in Table S10.

$\frac{M_x}{M_y} = \frac{I_x}{I_y} \times \frac{N_y}{N_x}$ (Eq. S1), where *I* is the integral and *N* is the number of nuclei giving rise to the signal.

Table S 10. NMR quantification using Eq. S1.

Compounds	Molar ratio
$MeSiCl_2OH:MeSiCl_3$	1:0.07
$MeSiCl_2OH:Me_2SiCl_2$	1:0.05
$MeSiCl_2OH:(MeSiCl_2)_2O$	1:0.20

NMR spectra for the depolymerization mixture of 'SmallMW' using Method B

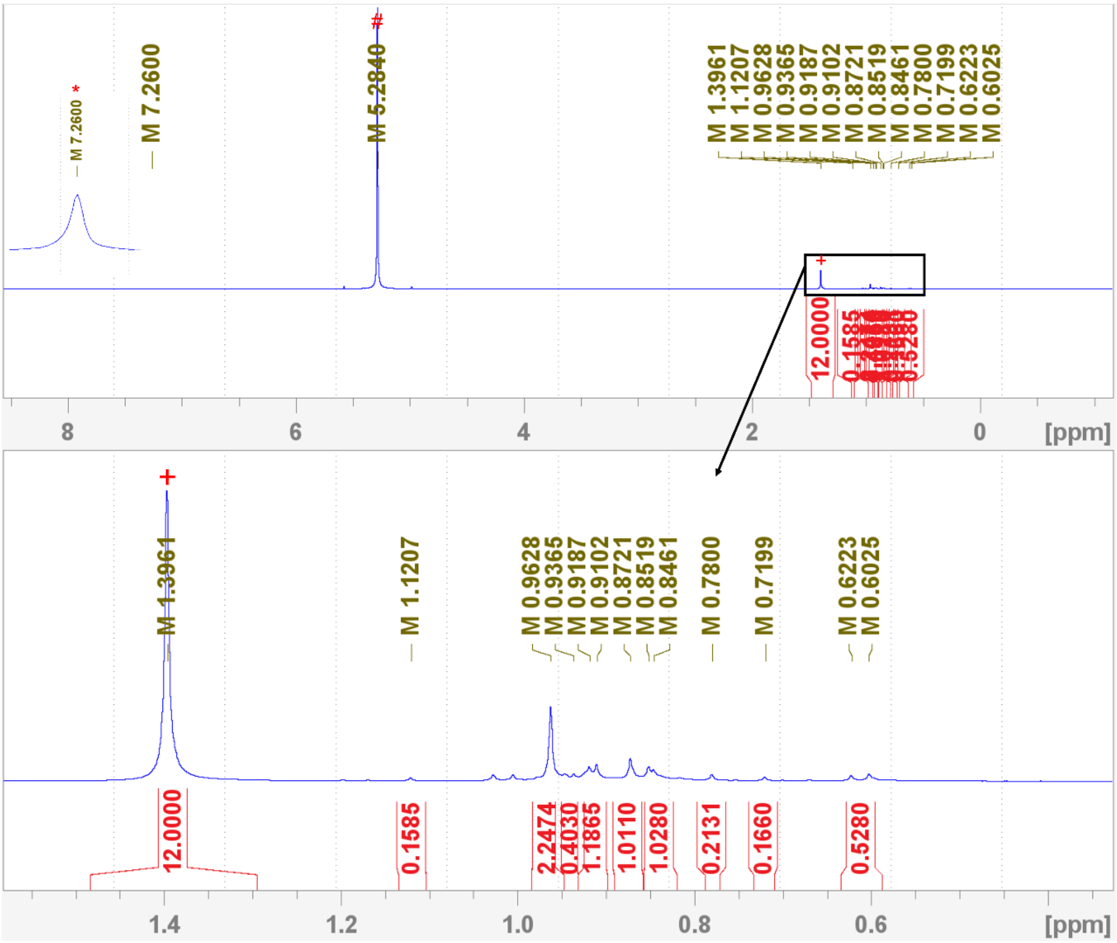

Figure S 83. ^{1}H NMR spectrum of the reaction mixture resulting from the depolymerization of **SmallMW** using Method B ($BCl_3/AlCl_3/FeCl_3$@cat./CH_2Cl_2/45C@22h). Solvent mixture: $CDCl_3$(**#**)/CH_2Cl_2(*) in ca. 1:1 v/v. Internal standard: 44.3 mg of cyclohexane (**+**) (overall integration amounts to 0.526 mmol). Spectrometer acquisition parameters: 300 MHz, 298 K.

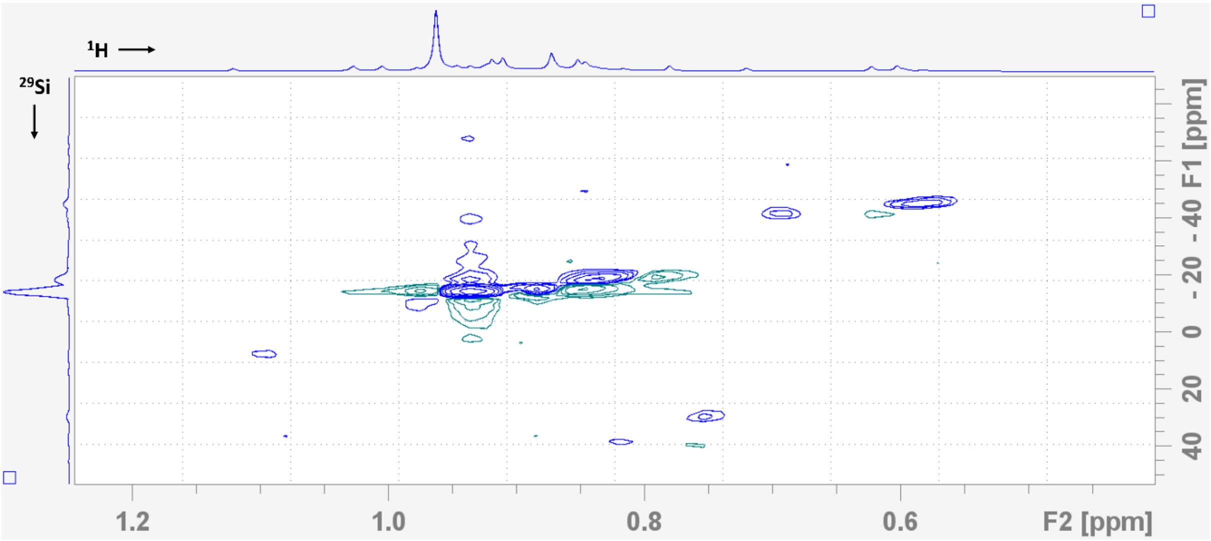

Figure S 84. ^{29}Si NMR spectrum of the reaction mixture resulting from the depolymerization of **SmallMW** using Method B ($BCl_3/AlCl_3/FeCl_3$@cat./CH_2Cl_2/45C@22h). Solvent mixture: $CDCl_3/CH_2Cl_2$ in ca. 1:1 v/v. Spectrometer acquisition parameters: 300/59.6 MHz, 298 K.

^{1}H NMR data in ppm ($CDCl_3$, 300 MHz, 298 K). $MeSiCl_3$[54]: 2.12 (s, Si-*Me*); $MeSiCl_2OH$[51]: 0.96 (bs, Si-*Me*); $(MeSiCl_2)_2O$[55]: 0.87 (bs, Si-*Me*); Me_2SiCl_2[49]: 0.78 (s, Me). **^{1}H-^{29}Si NMR data in ppm ($CDCl_3$, 300/59.6 MHz, 298 K).** Me_2SiCl_2[49]: 31.9; $MeSiCl_3$[56]: 11.2; $MeSiCl_2OH$[51]: –12.0; $(MeSiCl_2)_2O$[55]: –16.4; unknown (minor): –38.9, –42.7.

Additional notes on possible ways to improve the sustainability of the depolymerization method B:

Although a stoichiometric excess of BCl_3 was employed to ensure complete depolymerization on a laboratory scale, the high volatility of the reagents and the selective formation of monomeric chlorosilanes (e.g., $MeSiCl_3$) make this process highly amenable to standard industrial recycling and fractional distillation.

i. Excess reagents and BCl_3 recycling:

While a large excess of BCl_3 (5.6 equiv) was utilized on our laboratory scale to ensure complete kinetic conversion and overcome the heavy cross-linking of the resin, this excess is highly recoverable. BCl_3 is a low-boiling compound (b.p. 12.6 °C). In an industrial or scaled-up setting, the unreacted BCl_3 gas can be easily recovered from the reaction mixture via simple cryogenic trapping or condensation and recycled directly into the next batch, significantly mitigating the atom economy and safety concerns associated with its initial excess.

ii. The origin of $MeSiCl_3$ and $MeSiCl_2OH$:

The formation of $MeSiCl_3$ is not a side reaction, but rather the expected theoretical product of complete depolymerization. During the H_2 generation phase, the linear PMHS chain cross-links by converting Si–H bonds into Si–O–Si linkages, effectively forming a T-type silicone resin $[MeSi(O)_{3/2}]_n$. Consequently, the complete cleavage of all three Si–O bonds on each silicon atom by BCl_3 naturally yields methyltrichlorosilane ($MeSiCl_3$).

Conversely, the trace presence of $MeSiCl_2OH$ is almost certainly the result of partial adventitious hydrolysis of the highly moisture-sensitive $MeSiCl_3$ product during the reaction work-up and GC/NMR analysis phases, rather than incomplete chlorination.

iii. Selectivity and separation for silicone synthesis:

The depolymerization exhibits excellent selectivity for monomeric chlorosilanes over oligomers, successfully breaking down the rigid polymer network. Because these resulting monomers ($MeSiCl_3$, Me_2SiCl_2, etc.) are volatile liquids, they are perfectly suited for separation via standard fractional distillation. This is a massive advantage for circularity, as fractional distillation is the exact, pre-existing technology the silicone industry currently uses to separate the complex chlorosilane mixtures generated by the Müller-Rochow process.[57]

13. References

[1] C. P. Yap, H. T. Poh, W. Y. Fan, RSC Adv. 2016, 6, 5903-5906.

[2] J. M. Brunel, *Int. J. Hydrogen Energy* **2017**, *42*, 23004-23009.

[3] H. Wu, L.-L. Zhang, J. Wang, Y. Jiang, H. Li, P. Sudarsanam, S. Yang, *Green Chem.* **2021**, *23*, 7528-7533.

[4] E. A. Ison, R. A. Corbin, M. M. Abu-Omar, *J. Am. Chem. Soc.* **2005**, *127*, 11938-11939.

[5] N. Almenara, M. A. Garralda, X. Lopez, J. M. Matxain, Z. Freixa, M. A. Huertos, *Angew. Chem. Int. Ed.* **2022**, *61*.

[6] U. Prieto-Pascual, A. Rodríguez-Diéguez, Z. Freixa, M. A. Huertos, *Inorg. Chem.* **2023**, *62*, 3095-3105.

[7] W. Sattler, G. Parkin, *J. Am. Chem. Soc.* **2012**, *134*, 17462-17465.

[8] A. K. L. Teo, W. Y. Fan, *RSC Adv.* **2014**, *4*, 37645-37648.

[9] TURBOMOLE V7.9 2024, a development of University of Karlsruhe and Forschungszentrum Karlsruhe GmbH, 1989-2007, TURBOMOLE GmbH, since 2007; available from https://www.turbomole.org

[10] Electronic Structure Calculations on Workstation Computers: The Program System TURBOMOLE. R. A hlrichs, M. Bär, M. Häser, H. Horn and C. Kölmel; Chem. Phys. Lett., 162, 165 (1989).

[11] Von Arnim, M.; Ahlrichs, R. Performance of Parallel TURBOMOLE for Density Functional Calculations. J. Comput. Chem. 1998, 19, 1746–1757, doi:10.1002/(SICI)1096-987X(19981130)19:15<1746::AID-JCC7>3.0.CO;2-N.

[12] Becke, A.D. Density-Functional Exchange-Energy Approximation with Correct Asymptotic Behavior. Phys. Rev. A 1988, 38, 3098–3100, doi:10.1103/PhysRevA.38.3098.

[13] Becke, A.D. Density-functional Thermochemistry. III. The Role of Exact Exchange. J. Chem. Phys. 1993, 98, 5648–5652, doi:10.1063/1.464913.

[14] Lee, C.; Yang, W.; Parr, R.G. Development of the Colle-Salvetti Correlation-Energy Formula into a Functional of the Electron Density. Phys. Rev. B 1988, 37, 785–789, doi:10.1103/PhysRevB.37.785.

[15] Efficient Molecular Numerical Integration Schemes. O. Treutler and R. Ahlrichs; J. Chem. Phys., 102, 346 (1995)

[16] Weigend, F.; Ahlrichs, R. Balanced Basis Sets of Split Valence, Triple Zeta Valence and Quadruple Zeta Valence Quality for H to Rn: Design and Assessment of Accuracy. Phys. Chem. Chem. Phys. 2005, 7, 3297–3305, doi:10.1039/B508541A

[17] Grimme, S.; Antony, J.; Ehrlich, S.; Krieg, H. A Consistent and Accurate*Ab Initio*Parametrization of Density Functional Dispersion Correction (DFT-D) for the 94 Elements H-Pu. *J. Chem. Phys.* **2010**, *132*, doi:10.1063/1.3382344.

[18] Grimme, S.; Ehrlich, S.; Goerigk, L. Effect of the Damping Function in Dispersion Corrected Density Functional Theory. *J. Comput. Chem.* **2011**, *32*, 1456–1465, doi:10.1002/jcc.21759

[19] An efficient implementation of second analytical derivatives for density functional methods. P. Deglmann, F. Furche and R. Ahlrichs; Chem. Phys. Lett., 362, 511 (2002)

[20] Efficient characterization of stationary points on potential energy surfaces. P. Deglmann and F. Furche; J. Chem. Phys., 117, 9535 (2002).

[21] Klamt, A.; Schüürmann, G. COSMO: A New Approach to Dielectric Screening in Solvents with Explicit Expressions for the Screening Energy and Its Gradient. J. Chem. Soc. Perkin Trans. 2 1993, 799–805, doi:10.1039/P29930000799.

[22] https://doi.org/10.1021/acs.jctc.4c00052

[23] Weigend, F. Accurate Coulomb-Fitting Basis Sets for H to Rn. Phys. Chem. Chem. Phys. 2006, 8, 1057–1065, doi:10.1039/B515623H.

[24] Eichkorn, K.; Treutler, O.; Öhm, H.; Häser, M.; Ahlrichs, R. Auxiliary Basis Sets to Approximate Coulomb Potentials. *Chem. Phys. Lett.* **1995**, *240*, 283–290, doi:10.1016/0009-2614(95)00621-A.

[25] Eichkorn, K.; Treutler, O.; Öhm, H.; Häser, M.; Ahlrichs, R. Auxiliary Basis Sets to Approximate Coulomb Potentials (Chem. Phys. Letters 240 (1995) 283-290). *Chem. Phys. Lett.* **1995**, *242*, 652–660, doi:10.1016/0009-2614(95)00838-U.

[26] Eichkorn, K.; Weigend, F.; Treutler, O.; Ahlrichs, R. Auxiliary Basis Sets for Main Row Atoms and Transition Metals and Their Use to Approximate Coulomb Potentials. *Theor. Chem. Acc.* **1997**, *97*, 119–124, doi:10.1007/s002140050244.

[27] Weigend, F. A Fully Direct RI-HF Algorithm: Implementation, Optimised Auxiliary Basis Sets, Demonstration of Accuracy and Efficiency. *Phys. Chem. Chem. Phys.* **2002**, *4*, 4285–4291, doi:10.1039/B204199P.

[28] Nuclear second analytical derivative calculations using auxiliary basis set expansion. P. Deglmann, K. May, F. Furche and R. Ahlrichs; Chem. Phys. Lett., 384, 103, (2004).
[29] P. Plessow. Reaction path optimization without neb springs or interpolation algorithms. J. Chem. Theory Comput., 9(3), 1305–1310, (2013)
[30] Jmol: an open-source Java viewer for chemical structures in 3D. http://www.jmol.org/
[31] Rösch, L.; John, P.; Reitmeier, R. (2003). Organic Silicon Compounds. In Ullmann's Encyclopedia of Industrial Chemistry. Weinheim: Wiley-VCH. doi:10.1002/14356007.a24_021
[32] W. S. Matthews, J. E. Bares, J. E. Bartmess, F. G. Bordwell, F. J. Cornforth, G. E. Drucker, Z. Margolin, R. J. McCallum, G. J. McCollum, N. R. Vanier, *J. Am. Chem. Soc.* **1975**, *97*, 7006-7014.
[33] W. N. Olmstead, Z. Margolin, F. G. Bordwell, *J. Org. Chem* **1980**, *45*, 3295-3299.
[34] F. G. Bordwell, D. Algrim, *J. Org. Chem* **1976**, *41*, 2507-2508.
[35] S. T. Heller, T. P. Silverstein, *ChemTexts* **2020**, 6.
[36] Shimadzu News, can be found under https://www.shimadzu.com/an/sites/shimadzu.com.an/files/pim/pim_document_file/journal/shimadzu_journal/12576/egc108003.pd, 3/2008 (accessed: October 10, 2024)
[37] J. P. Barham, G. Coulthard, K. J. Emery, E. Doni, F. Cumine, G. Nocera, M. P. John, L. E. A. Berlouis, T. McGuire, T. Tuttle, J. A. Murphy, *J.Am.Chem.Soc.* **2016**, *138*, 7402-7410.
[38] E. G. Bagryanskaya, S. R. A. Marque, *Chem.Rev.* **2014**, *114*, 5011-5056.
[39] W. Xie, S.-W. Park, H. Jung, D. Kim, M.-H. Baik, S. Chang, *J. Am. Chem. Soc.* **2018**, 140, 9659-9668.
[40] R. Corriu, C. Guérin, B. Henner, Q. Wang, *Inorg. Chim. Acta* **1992**, *198-200*, 705-713.
[41] I. D. Jenkins, K. H. Chow, E. H. Krenske, *Angew. Chem. Int. Ed.* **2025** 64, e202517336, https://doi.org/10.1002/anie.202517336
[42] E. D. Voronova, I. E. Golub, A. Pavlov, N. V. Belkova, O. A. Filippov, L. M. Epstein, E. S. Shubina, *Inorg. Chem.* **2020**, *59*, 12240-12251.
[43] A. J. Parker, *Q. Rev., Chem. Soc.* **1962**, *16*, 163.
[44] D. Martin, A. Weise, H.-J. Niclas, *Angew. Chem. Int. Ed.* **1967**, *6*, 318-334.
[45] C. F. Bernasconi, M. Kaufmann, H. Zollinger, *Helv. Chim. Acta* **1966**, *49*, 2563-2570.
[46] R. Kalfat, F. Babonneau, N. J. Gharbi, H. D. Zarrouk, *J. Mater. Chem.* **1996**, *6*, 1673.
[47] M. Itoh, F. Oka, M. Suto, S. D. Cook, N. Auner, *International Journal of Polymer Science* **2012**, *2012*, 1-17.
[48] W.-P. Chuang, Y.-C. Sheen, S.-M. Wei, M.-Y. Yen, C.-C. M. Ma, *Eur. Polym. J.* **2013**, *49*, 646-651.
[49] N. Đ. Vũ, A. Boulegue-Mondière, N. Durand, J. Munsch, M. Boste, R. Lhermet, D. Gajan, A. Baudouin, S. Roldán-Gómez, M.-E. L. Perrin, V. Monteil, J. Raynaud, *Science* **2025**, *388*, 392-400.
[50] R. T. Larsen, M. McLaughlin, D. E. Katsoulis, *Industrial & Engineering Chemistry Research* **2022**, *61*, 9206-9217.
[51] M. Itoh, R. W. Lenz, *J. Polym. Sci., Part A: Polym. Chem.* **1991**, *29*, 1399-1406.
[52] PATENT # JP2022151955A
[53] PATENT # WO2019060479A1
[54] S. Sergani, I. Kalikhman, S. Yakubovich, D. Kost, *Organometallics* **2007**, *26*, 5799-5802.
[55] S. V. Basenko, A. A. Maylyan, A. S. Soldatenko, *Silicon* **2018**, *10*, 465-470.
[56] H. Marsmann, Springer Berlin Heidelberg, **1981**, pp. 65-235.
[57] Rösch, L.; John, P.; Reitmeier, R. (2003). Organic Silicon Compounds. In *Ullmann's Encyclopedia of Industrial Chemistry*. Weinheim: Wiley-VCH. doi:10.1002/14356007.a24_021
